# Self-Adjoint Dirac Operators on Domains in $\mathbb {R}^3$

**DOI:** 10.1007/s00023-020-00925-1

**Published:** 2020-06-20

**Authors:** Jussi Behrndt, Markus Holzmann, Albert Mas

**Affiliations:** 1 Institut für Angewandte Mathematik, Technische Universität Graz, Steyrergasse 30, 8010 Graz, Austria; 2 Departament de Matemàtiques, Universitat Politècnica de Catalunya, Campus Diagonal Besòs, Edifici A (EEBE), Av. Eduard Maristany 16, 08019 Barcelona, Spain

**Keywords:** Primary 81Q10, Secondary 35Q40

## Abstract

In this paper, the spectral and scattering properties of a family of self-adjoint Dirac operators in $L^2(\Omega ; \mathbb {C}^4)$, where $\Omega \subset \mathbb {R}^3$ is either a bounded or an unbounded domain with a compact $C^2$-smooth boundary, are studied in a systematic way. These operators can be viewed as the natural relativistic counterpart of Laplacians with boundary conditions as of Robin type. Our approach is based on abstract boundary triple techniques from extension theory of symmetric operators and a thorough study of certain classes of (boundary) integral operators, that appear in a Krein-type resolvent formula. The analysis of the perturbation term in this formula leads to a description of the spectrum and a Birman–Schwinger principle, a qualitative understanding of the scattering properties in the case that $\Omega$ is an exterior domain, and corresponding trace formulas.

## Introduction

In recent years, the mathematical study of Dirac operators acting on domains $\Omega \subset \mathbb {R}^d$ with special boundary conditions that make them self-adjoint gained a lot of attention. The motivation for this arises from several aspects: From the physical point of view, they are used in relativistic quantum mechanics to describe particles that are confined to a predefined area or box. One important model in 3D (dimension three) is the MIT bag model suggested in the 1970s by physicists in [30–32, 34, 43] to study confinement of quarks. In the 2D (dimension two) case, Dirac operators with special boundary conditions similar to the MIT bag model are used in the description of graphene; cf. [1, 25, 29, 58]. From the mathematical point of view, Dirac operators with special boundary conditions can be seen as the relativistic counterpart of Laplacians with boundary conditions as, e.g., of Robin type. Moreover, Dirac operators with boundary conditions are also closely related to Dirac operators with singular $\delta$–shell interactions supported on surfaces for special choices of the interaction strengths in the so-called confinement case, i.e., when the $\delta$-potential is impenetrable for the particle; cf. [5, 12, 16, 37].

To set the stage, let $\Omega \subset \mathbb {R}^3$ be either a bounded or unbounded domain with a compact $C^2$-smooth boundary and let $\nu$ be the unit normal vector field at $\partial \Omega$ which points outwards of $\Omega$. Choose units such that the Planck constant $\hbar$ and the speed of light are both equal to one. Moreover, assume that $\vartheta : \partial \Omega \rightarrow \mathbb {R}$ is a Hölder continuous function of order $a>\frac{1}{2}$, denoted by $\vartheta \in \mathrm {Lip}_a({\partial \Omega })$, and consider in $L^2(\Omega ; \mathbb {C}^4)$ the operator

1.1 $$\begin{aligned} A_\vartheta f&= -\,i \alpha \cdot \nabla f + m \beta f= -i \sum _{j=1}^3 \alpha _j \cdot \partial _j f + m \beta f, \nonumber \\ \mathrm {dom}\,A_\vartheta&= \big \{ f \in H^1(\Omega ; \mathbb {C}^4): \vartheta \big (I_4 + i \beta (\alpha \cdot \nu )\big ) f|_{\partial \Omega } = \big (I_4 + i \beta (\alpha \cdot \nu )\big ) \beta f|_{\partial \Omega } \big \}, \end{aligned}$$

where $\alpha = (\alpha _1, \alpha _2, \alpha _3)$ and $\beta$ are the $\mathbb {C}^{4 \times 4}$ Dirac matrices defined in (1.6) and $\alpha \cdot x = \alpha _1 x_1 + \alpha _2 x_2 + \alpha _3 x_3$ for $x = (x_1, x_2, x_3)^\top \in \mathbb {R}^3$. The time-dependent equation with the Hamiltonian given by $A_\vartheta$ models the propagation of a relativistic particle subject to the boundary conditions in $\mathrm {dom}\,A_\vartheta$ with mass $m > 0$ contained in $\Omega$.

The existing mathematical literature on such types of Dirac operators contains different approaches. In differential geometry, there are several articles dealing with self-adjoint Dirac operators on smooth manifolds, see, for instance, [7, 8, 59]. The class of boundary conditions treated in [8] contains also the physically particularly interesting MIT bag boundary conditions, which will be rigorously defined below and which yield a vanishing normal flux at the boundary of $\Omega \subset \mathbb R^3$. It was already shown in [61] that the 2D Dirac operator with so-called zigzag boundary conditions (in the massless case) is self-adjoint and that zero is an eigenvalue of infinite multiplicity, see also [39]. The zigzag boundary conditions arise from the termination of a lattice in a graphene quantum dot, when the direction of the boundary is perpendicular to the bonds [41]. Very recent related publications in the 2D case are [22, 23], where the self-adjointness of Dirac operators in bounded $C^2$-domains $\Omega \subset \mathbb {R}^2$ for a wide class of boundary conditions describing quantum dots was shown. Many considerations in [22, 23, 61] are based on complex analysis techniques, which are not available in the 3D situation. We also refer to [28, 47, 48, 57] for self-adjointness and spectral problems of 2D Dirac operators on different types of domains with special boundary conditions. In contrast to the 2D setting, $A_\vartheta$ was not directly investigated for general boundary parameters in 3D, as far as we know only the particular MIT bag operator is well studied. We emphasize the recent papers [2, 56] for the analysis of general properties of the MIT bag operator and [3, 9, 24, 54, 62], where it is shown that the MIT bag boundary conditions and their 2D analogues can be interpreted as infinite mass boundary conditions (i.e., $\Omega$ is surrounded by a medium with infinite mass). The strategy developed in [56] employing Calderón projections can also be used to study the self-adjointness of Dirac operators of the form (1.1). However, this approach does not allow directly a systematic spectral analysis of these operators. Finally, we mention that in recent years the self-adjointness and the spectral and scattering properties of the closely related Dirac operators with singular $\delta$–shell interactions were studied comprehensively in [4–6, 10, 12, 15, 16, 42, 49–51, 55, 56].

The main objective of this paper is to develop a systematic approach to the spectral analysis and scattering theory for self-adjoint Dirac operators in the 3D case. Here, we are particularly interested in boundary conditions as in (1.1), since these are the 3D analogue of the 2D boundary conditions in [22] used to describe graphene quantum dots (cf. Remark 5.2). Furthermore, Dirac operators with boundary conditions as in (1.1) can be viewed as relativistic counterparts of Schrödinger operators with Robin-type (and possibly other) boundary conditions, as it is argued for the MIT bag model in [2]. This could be made rigorous by computing the non-relativistic limit [63, Chapter 6]. Another important goal in the present paper is to allow variable parameters in the boundary conditions. To the best of our knowledge, this is a novelty in the 3D case and it requires substantial technical effort. We believe that many of our results in this regard are also of interest for studying similar problems for Dirac operators with singular $\delta$–shell interactions with varying strength.

Our mathematical treatment of the operators $A_\vartheta$ in (1.1) is based on the application of a suitable so-called quasi boundary triple. Quasi boundary triples and their Weyl functions are an abstract concept from extension and spectral theory for symmetric and self-adjoint operators which were originally introduced to investigate boundary value problems for elliptic partial differential operators in [17], but proved to be useful in many other situations, see, e.g., [11, 19, 20]. Quasi boundary triples were also applied more recently in [10, 15] to Dirac operators with singular potentials. Once a quasi boundary triple and Weyl function in the present situation are available, they allow to deduce in an efficient way the spectral properties of $A_\vartheta$ from the properties of certain (boundary) integral operators which are induced by the Green’s function of the free Dirac operator in $\mathbb {R}^3$. These operators also appeared in [4, 5, 10, 15, 56] in the study of Dirac operators with $\delta$–shell potentials, and many of their properties were derived there; see also [26, 33, 52] for earlier results related to this framework. In the present paper, in particular to handle non-constant boundary parameters $\vartheta$, additional mapping properties of these integral operators are required and, in fact, this analysis covers a great part of this paper. We would like to point out that this approach is independent of the space dimension.

One of the key features in the quasi boundary triple approach is a Krein-type resolvent formula that relates the resolvent of $A_\vartheta$ via a perturbation term to the resolvent of a reference operator, which in our model is the MIT bag operator $T_{\mathrm{MIT}}$. More precisely, making use of the quasi boundary triple in Theorem 4.1 and the properties of the corresponding $\gamma$-field $\gamma$ and Weyl function *M* in Proposition 4.2 we conclude the self-adjointness of $A_\vartheta$ in $L^2(\Omega ; \mathbb {C}^4)$ and the relation

1.2 $$\begin{aligned} (A_\vartheta -\lambda )^{-1} = \big (T_{\mathrm{MIT}} - \lambda \big )^{-1} + \gamma (\lambda ) \big ( \vartheta - M(\lambda ) \big )^{-1} \gamma (\overline{\lambda })^* \end{aligned}$$

for all $\lambda \in \rho (A_{\vartheta })\cap \rho (T_{\mathrm{MIT}})$, where $\vartheta \in \mathrm {Lip}_a({\partial \Omega })$ is any real-valued Hölder continuous function with $a>\frac{1}{2}$; cf. Theorem 5.3. Our arguments here also rely on the self-adjointness of the reference operator $T_{\mathrm{MIT}}$ proved in [8, 56] for $C^2$ -boundaries; cf. Proposition 3.3. Based on (1.2), we show several spectral and scattering properties of $A_\vartheta$, which are, of course, different for bounded and for unbounded domains $\Omega$. It turns out in Theorem 5.4 that in the case of an unbounded domain $\Omega$ with a compact $C^2$-boundary the essential spectrum of $A_\vartheta$ is given by $(-\infty , -m] \cup [m, \infty )$, and there are at most finitely many discrete eigenvalues in the gap $(-m,m)$ that can be characterized by a Birman–Schwinger principle implied by (1.2), which states that $\lambda \in \sigma _{\mathrm{p}}(A_\vartheta ) \cap (-m, m)$ if and only if $0 \in \sigma _\mathrm{p}(\vartheta - M(\lambda ))$. Furthermore, in Theorem 5.7 we provide Schatten–von Neumann estimates on the differences of resolvent powers of $A_\vartheta$ and $T_{\mathrm{MIT}}$ and, in particular, conclude in Corollary 5.8 that the resolvent power difference $(A_\vartheta - \lambda )^{-3} - ( T_{\mathrm{MIT}} - \lambda )^{-3}$ is a trace class operator for any $\lambda \in \mathbb {C} {\setminus } \mathbb {R}$, which leads to a trace formula and also implies the existence and completeness of the wave operators for the scattering pair $\{A_\vartheta ,T_{\mathrm{MIT}}\}$. If $\Omega$ is a bounded $C^2$-domain, then the spectrum of $A_\vartheta$ is purely discrete and all eigenvalues of $A_\vartheta$ can be characterized by a modified Birman–Schwinger principle formulated in Proposition 5.5, which again can be viewed as a consequence of the abstract quasi boundary triple approach.

The above-mentioned results are proved for any real-valued $\vartheta \in \mathrm {Lip}_a({\partial \Omega })$ with $a>\frac{1}{2}$ under the additional assumption $\vartheta (x)^2 \not = 1$ for all $x\in \partial \Omega$, which we refer to as the *non-critical case*. We expect that in the *critical case*$\vartheta (x)^2 = 1$ for some $x\in \partial \Omega$ the spectral properties of $A_\vartheta$ may significantly differ from the non-critical case, e.g., essential spectrum may arise also for bounded domains or in the gap $(-m,m)$. Similar difficulties and effects were observed in the 2D situation in [22, 61] and also in the analysis of Dirac operators with singular interactions in [4, 10, 15, 16, 56].

For some models, it is more convenient to consider Dirac operators $A_{[\omega ]}$ in $L^2(\Omega ; \mathbb {C}^4)$ with boundary conditions of the form

1.3 $$\begin{aligned} \big (I_4 + i \beta (\alpha \cdot \nu )\big ) f|_{\partial \Omega } = \omega \big (I_4 + i \beta (\alpha \cdot \nu )\big ) \beta f|_{\partial \Omega }, \end{aligned}$$

where again $\omega \in \mathrm {Lip}_a({\partial \Omega })$ is any real-valued Hölder continuous function with $a>\frac{1}{2}$. Comparing with the boundary conditions in (1.1), one formally has $\omega =\vartheta ^{-1}$. Note that the particularly interesting MIT bag model corresponds to $\omega \equiv 0$. Using the abstract quasi boundary triple approach, the spectral and scattering properties of $A_{[\omega ]}$ can be studied in the same way as those of $A_\vartheta$, and similar results as sketched above for $A_\vartheta$ follow; cf. Sect. 5.2.

In the last part of this paper, we briefly explain the connection of the Dirac operators $A_\vartheta$ and $A_{[\omega ]}$ on domains with Dirac operators with $\delta$–shell interactions. More precisely, operators and boundary conditions of the form (1.1) and (1.3) appear in the treatment of Dirac operators

1.4 $$\begin{aligned} B_{\eta , \tau } = -i \alpha \cdot \nabla + m \beta + (\eta I_4 + \tau \beta ) \delta _{\Sigma } \end{aligned}$$

in $\mathbb R^3$ with singular $\delta$–shell potentials supported on $\Sigma =\partial \Omega _\pm$ in the confinement (or decoupling) case, where $\Omega _+$ is a bounded $C^2$-domain and $\Omega _-=\mathbb R^3{\setminus }\overline{\Omega _+}$ is an exterior domain. Note that so far such operators $B_{\eta , \tau }$ were only studied for constant interaction strengths $\eta ,\tau \in \mathbb R$. It is known that for $\eta ^2 - \tau ^2 = -4$ the operator $B_{\eta , \tau }$ can be written as the orthogonal sum of operators acting in $L^2(\Omega _+; \mathbb {C}^4)$ and $L^2(\Omega _-; \mathbb {C}^4)$, respectively, and it turns out that these operators are exactly of the form (1.1) with a certain constant $\vartheta \in \mathbb R$. In Sect. 5.3, we allow variable real-valued coefficients $\vartheta ,\eta ,\tau \in \mathrm {Lip}_a({\partial \Omega })$ with $a >\frac{1}{2}$, and we specify in Proposition 5.14 relations between the functions $\vartheta ,\eta ,\tau$ such that

1.5 $$\begin{aligned} B_{\eta , \tau }=A_\vartheta ^{\Omega _+} \oplus A_\vartheta ^{\Omega _-} \end{aligned}$$

holds, where $A_\vartheta ^{\Omega _\pm }$ denote the self-adjoint Dirac operators defined in (1.1) acting in $L^2(\Omega _\pm ; \mathbb {C}^4)$. With the help of such identities, one can then translate results for Dirac operators $A_\vartheta ^{\Omega _\pm }$ to Dirac operators with $\delta$-interactions with variable interaction strengths $\eta ,\tau$, and vice versa; cf. Lemma 5.16 and Theorem 5.17 for a simple illustration of this idea. With the same approach, we also study the relationship between $A_{[\omega ]}^{\Omega _\pm }$ and $B_{\eta , \tau }$, and the results are also described in detail in Sect. 5.3. From a physical point of view, the orthogonal decoupling in (1.5) means that $A_\vartheta ^{\Omega _\pm }$ and $A_{[\omega ]}^{\Omega _\pm }$ can be used to describe a relativistic particle actually living in $\mathbb {R}^3$, but which is confined to $\Omega _+$ or $\Omega _-$ for all time, see [5, Section 5].

### Structure of the Paper

In Sect. 2, we review the definitions of quasi boundary triples and their associated Weyl functions. Then, in Sect. 3 we recall some knowledge on a minimal and a maximal realization of the Dirac operator in $\Omega$, the MIT bag model, and the properties of several families of integral operators associated with the resolvent of the free Dirac operator. Next, in Sect. 4 we introduce and study a quasi boundary triple which is suitable to investigate Dirac operators in $\Omega$ with boundary conditions. Section 5 contains the main results of the present paper. In Sect. 5.1, we first define $A_\vartheta$ with the help of the quasi boundary triple from Sect. 4 and then conclude its self-adjointness and the resolvent formula in Theorem 5.3. This allows to prove various spectral properties in Theorem 5.4, Proposition 5.5, Theorem 5.7, and Corollary 5.8. Section 5.2 is then devoted to the study of the operator $A_{[\omega ]}$ with the boundary conditions (1.3), while in Sect. 5.3 we discuss the above-mentioned connection of $A_\vartheta$ and $A_{[\omega ]}$ to the operator $B_{\eta , \tau }$ formally given in (1.4). Finally, in “Appendix A” we collect some material on integral operators and their mapping properties in Sobolev spaces on the boundary $\partial \Omega$, which is applied in the proofs of the main results of this paper.

### Notations

Throughout this paper, *m* is always a positive constant that stands for the mass of a particle. The Dirac matrices $\alpha _1, \alpha _2, \alpha _3, \beta \in \mathbb {C}^{4 \times 4}$ are defined by

1.6 $$\begin{aligned} \alpha _j := \begin{pmatrix} 0 &{}\quad \sigma _j \\ \sigma _j &{}\quad 0 \end{pmatrix} \quad \text {and} \quad \beta := \begin{pmatrix} I_2 &{}\quad 0 \\ 0 &{}\quad -I_2 \end{pmatrix}, \end{aligned}$$

where $I_n$ is the $n \times n$-identity matrix and $\sigma _j$, $j \in \{ 1, 2, 3 \}$, are the Pauli spin matrices

1.7 $$\begin{aligned} \sigma _1 := \begin{pmatrix} 0 &{}\quad 1 \\ 1 &{}\quad 0 \end{pmatrix}, \qquad \sigma _2 := \begin{pmatrix} 0 &{}\quad -i \\ i &{}\quad 0 \end{pmatrix}, \qquad \sigma _3 := \begin{pmatrix} 1 &{}\quad 0 \\ 0 &{}\quad -1 \end{pmatrix}. \end{aligned}$$

The Dirac matrices satisfy the anti-commutation relations

1.8 $$\begin{aligned} \alpha _j\alpha _k + \alpha _k\alpha _j = 2\delta _{jk}I_4,\quad \text {and} \quad \alpha _j \beta + \beta \alpha _j = 0, \qquad j,k\in \{1,2,3\}. \end{aligned}$$

For vectors $x = (x_1, x_2, x_3)^{\top }\in \mathbb {R}^3$, we shall often use the notation $\alpha \cdot x := \sum _{j=1}^3 \alpha _j x_j$.

The upper/lower complex half plane is denoted by $\mathbb {C}_\pm$. The square root $\sqrt{\cdot }$ is fixed by $\mathrm {Im}\sqrt{\lambda }>0$ for $\lambda \in \mathbb C{\setminus } [0,\infty )$ and $\sqrt{\lambda }\ge 0$ for $\lambda \ge 0$. The open ball of radius $r > 0$ centered at $x \in \mathbb {R}^3$ is denoted by *B*(*x*, *r*). For a $C^2$-domain $\Omega \subset \mathbb {R}^3$, we write ${\partial \Omega }$ for its boundary and $\sigma$ is the two-dimensional Hausdorff measure on ${\partial \Omega }$. We shall mostly work with the $L^2$-spaces $L^2(\Omega ; \mathbb {C}^n)$ and $L^2({\partial \Omega }; \mathbb {C}^n)$ of $\mathbb C^n$-valued square integrable functions, the corresponding inner products being denoted by $(\cdot , \cdot )_\Omega$ and $(\cdot , \cdot )_{\partial \Omega }$, respectively. We write $C_0^\infty (\Omega ; \mathbb {C}^n)$ for the space of $\mathbb C^n$-valued smooth functions with compact support in $\Omega$ and we set

$$\begin{aligned} C^\infty (\overline{\Omega }; \mathbb {C}^n) := \big \{ f \upharpoonright \Omega : f \in C_0^\infty (\mathbb {R}^3; \mathbb {C}^n) \big \}. \end{aligned}$$

We write $H^k(\mathbb R^3; \mathbb {C}^n)$ for the usual $L^2(\mathbb R^3; \mathbb {C}^n)$-based Sobolev space of *k*-times weakly differentiable functions, and similarly $H^k(\Omega ; \mathbb {C}^n)$. In addition, $H^1_0(\Omega ; \mathbb {C}^n)$ denotes the closure of $C_0^\infty (\Omega ; \mathbb {C}^n)$ in $H^1(\Omega ; \mathbb {C}^n)$. Sobolev spaces on $C^l$-surfaces ${\partial \Omega }$, $l \in \mathbb {N}$, are denoted by $H^s({\partial \Omega }; \mathbb {C}^n)$, $s \in (0, l)$, and the symbol $H^{-s}(\partial \Omega ; \mathbb {C}^n)$ is used for their duals. The corresponding norm for $s \in (0, 1)$ is

1.9 $$\begin{aligned} \Vert f\Vert _s^2:=\int _{{\partial \Omega }}|f(x)|^2 \text {d} \sigma (x) +\int _{{\partial \Omega }}\int _{{\partial \Omega }} \frac{|f(x)-f(y)|^2}{|x-y|^{2+2s}} \text {d} \sigma (y) \text {d} \sigma (x). \end{aligned}$$

The trace of a function $f \in H^1(\Omega ; \mathbb {C}^n)$, which belongs by the trace theorem to $H^{1/2}(\partial \Omega ; \mathbb {C}^n)$, is denoted by $f|_{\partial \Omega }$. Eventually, given $0<a\le 1$ we denote the Hölder continuous functions on $\partial \Omega$ of order *a* by

$$\begin{aligned} \text {Lip}_a({\partial \Omega }):= \bigl \{f:{\partial \Omega }\rightarrow \mathbb {C}:\,|f(x)-f(y)|\le C|x-y|^a\text { for all }x,\,y\in {\partial \Omega }\bigr \}. \end{aligned}$$

For two Hilbert spaces $\mathcal {G}$ and $\mathcal {H}$, the space $\mathcal B(\mathcal {G}, \mathcal {H})$ is the set of all bounded and everywhere defined operators from $\mathcal {G}$ to $\mathcal {H}$. If $\mathcal {G} = \mathcal {H}$, then we simply write $\mathcal B(\mathcal {H})$. We write $\mathfrak {S}_{p, \infty }(\mathcal {G}, \mathcal {H})$ for the weak Schatten–von Neumann ideal of order $p>0$; this is the set of all compact operators $K: \mathcal {G} \rightarrow \mathcal {H}$ for which there exists a constant $\kappa$ such that the singular values $s_k(K)$ of *K* satisfy $s_k(K) \le \kappa k^{-1/p}$ for all $k \in \mathbb {N}$, see [40] or [19, Section 2.1]. Again we use $\mathfrak {S}_{p, \infty }(\mathcal {G})$ if $\mathcal {G} = \mathcal {H}$ and sometimes we suppress the spaces and just write $\mathfrak {S}_{p, \infty }$.

For a linear operator $T:\mathcal {G}\rightarrow \mathcal {H}$, we denote the domain, range, and kernel by $\mathrm {dom}\,T$, ${{\,\mathrm{ran}\,}}T$, and $\ker T$, respectively. If *T* is a self-adjoint operator in $\mathcal {H}$, then its resolvent set, spectrum, essential spectrum, discrete, and point spectrum are denoted by $\rho (T)$, $\sigma (T)$, $\sigma _{\text {ess}}(T)$, $\sigma _{\text {disc}}(T)$, and $\sigma _\mathrm{p}(T)$, respectively. Next, for a Banach space *X* we use the notation $(\cdot , \cdot )_{X'\times X}$ for the duality product in $X' \times X$ which is linear in the first and anti-linear in the second entry. Moreover, for $T \in \mathcal {B}(X, Y)$ we denote by $T' \in \mathcal {B}(Y', X')$ the anti-dual operator, which is uniquely determined by the relation

$$\begin{aligned} (y, T x)_{Y' \times Y} = (T' y, x)_{X' \times X} \end{aligned}$$

for all $x \in X$ and $y \in Y'$.

Finally, we call a closed symmetric operator *S* in a Hilbert space $\mathcal {H}$ simple, if for any orthogonal decomposition $\mathcal {H} = \mathcal {H}_1 \oplus \mathcal {H}_2$ such that $\mathcal {H}_1$ and $\mathcal {H}_2$ are invariant under *S* and $S_1 := S \upharpoonright \mathcal {H}_1$ is self-adjoint in $\mathcal {H}_1$ it follows $\mathcal {H}_1 = \{ 0 \}$. It was observed in [46] that a closed symmetric operator *S* is simple, if and only if

1.10 $$\begin{aligned} \overline{\text {span} \big \{ \ker (S^* - \lambda ) : \lambda \in \mathbb {C} {\setminus } \mathbb {R} \big \} } = \mathcal {H}. \end{aligned}$$

## quasi boundary Triples and Their Weyl Functions

This section is devoted to a short introduction to quasi boundary triples and their Weyl functions; the presentation is chosen such that the results can be applied directly in the main part of this paper. For a more detailed exposition and proofs in a general scenario we refer to [17, 18]. Throughout this abstract section, $\mathcal {H}$ is always a complex Hilbert space with inner product $(\cdot , \cdot )_\mathcal {H}$; if no confusion arises, we skip the index in the inner product.

### Definition 2.1

Let *S* be a densely defined, closed, symmetric operator in $\mathcal {H}$ and assume that *T* is a linear operator in $\mathcal {H}$ such that $\overline{T} = S^*$. Moreover, let $\mathcal {G}$ be a complex Hilbert space and let $\Gamma _0, \Gamma _1: \mathrm {dom}\,T \rightarrow \mathcal {G}$ be linear mappings. Then, $\{ \mathcal {G}, \Gamma _0, \Gamma _1 \}$ is called a *quasi boundary triple* for $T \subset S^*$ if the following conditions are fulfilled:

(i) For all $f, g \in \mathrm {dom}\,T$ the abstract Green’s identity
  $$\begin{aligned} (T f, g)_\mathcal {H} - (f, T g)_\mathcal {H} = (\Gamma _1 f, \Gamma _0 g)_\mathcal {G} - (\Gamma _0 f, \Gamma _1 g)_\mathcal {G} \end{aligned}$$
  holds.
(ii) $(\Gamma _0, \Gamma _1)^\top : \mathrm {dom}\,T \rightarrow \mathcal {G} \times \mathcal {G}$ has dense range.
(iii) The operator $A_0 := T \upharpoonright \ker \Gamma _0$ is self-adjoint in $\mathcal {H}$.

The concept of quasi boundary triples is a generalization of ordinary and generalized boundary triples; cf. [14, 27, 35, 36]. We note that the operator *T* in the above definition is not unique if the dimension of $\mathcal {G}$ is infinite. Moreover, we remark that a quasi boundary triple exists if and only if $\dim \ker (S^* - i) = \dim \ker (S^* + i)$, that is, if and only if *S* admits self-adjoint extensions in $\mathcal {H}$.

Next, we introduce the $\gamma$-field and the Weyl function associated with a given quasi boundary triple. Let $\{ \mathcal {G}, \Gamma _0, \Gamma _1 \}$ be a quasi boundary triple for $T \subset S^*$ and let $A_0 := T \upharpoonright \ker \Gamma _0$. The definition of the $\gamma$-field and the Weyl function is based on the direct sum decomposition

2.1 $$\begin{aligned} \mathrm {dom}\,T = \mathrm {dom}\,A_0 \dot{+} \ker (T - \lambda ) = \ker \Gamma _0 \dot{+} \ker (T - \lambda ), \quad \lambda \in \rho (A_0), \end{aligned}$$

and is formally the same as in the case of ordinary boundary triples, see [14, 35]. Note that (2.1) implies, in particular, that $\Gamma _0 \upharpoonright \ker (T-\lambda )$ is injective for $\lambda \in \rho (A_0)$.

### Definition 2.2

Let *S* be a densely defined, closed, symmetric operator in $\mathcal {H}$, let *T* be a linear operator such that $\overline{T} = S^*$, and let $\{ \mathcal {G}, \Gamma _0, \Gamma _1 \}$ be a quasi boundary triple for $T \subset S^*$.

(i) The $\gamma$*-field* associated with $\{ \mathcal {G}, \Gamma _0, \Gamma _1 \}$ is the mapping
  $$\begin{aligned} \rho (A_0) \ni \lambda \mapsto \gamma (\lambda ) := \bigl (\Gamma _0 \upharpoonright \ker (T-\lambda )\bigr )^{-1}. \end{aligned}$$
(ii) The *Weyl function* associated with $\{ \mathcal {G}, \Gamma _0, \Gamma _1 \}$ is the mapping
  $$\begin{aligned} \rho (A_0) \ni \lambda \mapsto M(\lambda ) := \Gamma _1 \bigl (\Gamma _0 \upharpoonright \ker (T-\lambda )\bigr )^{-1} = \Gamma _1 \gamma (\lambda ). \end{aligned}$$

Let us now assume that $\{ \mathcal {G}, \Gamma _0, \Gamma _1 \}$ is a quasi boundary triple for $T \subset S^*$ and set $A_0 := T \upharpoonright \ker \Gamma _0$. In the following, we collect several useful properties of the associated $\gamma$-field $\gamma$ and Weyl function *M*; for the proofs, see, for instance, [17, Proposition 2.6] and [18, Propositions 6.13 and 6.14]. First, for any $\lambda \in \rho (A_0)$ the mapping $\gamma (\lambda )$ is densely defined and bounded from $\mathcal {G}$ into $\mathcal {H}$ with $\mathrm {dom}\,\gamma (\lambda ) = {{\,\mathrm{ran}\,}}\Gamma _0$. Using the abstract Green’s identity, it is not difficult to see that the adjoint $\gamma (\lambda )^*: \mathcal {H} \rightarrow \mathcal {G}$ is given by $\gamma (\lambda )^* = \Gamma _1 (A_0 - \overline{\lambda })^{-1}$. This implies, in particular, $\gamma (\lambda )^* \in \mathcal {B}(\mathcal {H}, \mathcal {G})$. In a similar manner, we have for any $\lambda \in \rho (A_0)$ that the mapping $M(\lambda )$ is densely defined in $\mathcal {G}$ with $\mathrm {dom}\,M(\lambda ) = {{\,\mathrm{ran}\,}}\Gamma _0$ and ${{\,\mathrm{ran}\,}}M(\lambda ) \subset {{\,\mathrm{ran}\,}}\Gamma _1$. By definition, we have $M(\lambda ) \Gamma _0 f_\lambda = \Gamma _1 f_\lambda$ for $\lambda \in \rho (A_0)$ and $f_\lambda \in \ker (T-\lambda )$. Next, for any $\lambda , \mu \in \rho (A_0)$ and $\varphi \in {{\,\mathrm{ran}\,}}\Gamma _0$ the identity

$$\begin{aligned} M(\lambda ) \varphi = M(\mu )^* \varphi + (\lambda - \overline{\mu }) \gamma (\mu )^* \gamma (\lambda ) \varphi \end{aligned}$$

holds. In particular, the operator $M(\lambda )$ is closable, $M(\lambda ) \subset M(\overline{\lambda })^*$, and $M(\lambda )$ is symmetric for $\lambda \in \rho (A_0) \cap \mathbb {R}$.

In the main part of this paper, we will use quasi boundary triples to introduce special extensions of a symmetric operator *S*. Let $\{ \mathcal {G}, \Gamma _0, \Gamma _1 \}$ be a quasi boundary triple for $T \subset S^*$ and let $\Theta$ be a symmetric operator in $\mathcal {G}$. Then, we define the operator $A_\Theta$ acting in $\mathcal {H}$ by

2.2 $$\begin{aligned} A_\Theta := T \upharpoonright \ker (\Gamma _1 - \Theta \Gamma _0). \end{aligned}$$

In other words, a vector $f \in \mathrm {dom}\,T$ belongs to $\mathrm {dom}\,A_\Theta$ if $\Gamma _0 f \in \mathrm {dom}\,\Theta$ and if it satisfies the abstract boundary condition $\Gamma _1 f = \Theta \Gamma _0 f$. It follows immediately from the abstract Green’s identity that $A_\Theta$ is symmetric. Of course, one is typically interested in the self-adjointness of $A_\Theta$. However, in general, for quasi boundary triples the self-adjointness of $\Theta$ in $\mathcal {G}$ does not necessarily imply that $A_\Theta$ is self-adjoint in $\mathcal {H}$. Nevertheless, the next theorem provides an explicit Krein-type resolvent formula which allows to deduce several properties of $A_\Theta$ from $\Theta$; for a proof of this result see, for instance, [17, Theorem 2.8].

### Theorem 2.3

Let *S* be a densely defined, closed, symmetric operator in $\mathcal {H}$, let $\{ \mathcal {G}, \Gamma _0, \Gamma _1 \}$ be a quasi boundary triple for $T \subset S^*$, set $A_0 := T \upharpoonright \ker \Gamma _0$, and let $\gamma$ and *M* be the associated $\gamma$-field and Weyl function, respectively. Moreover, let $\Theta$ be a symmetric operator in $\mathcal {G}$ and let the associated operator $A_\Theta$ be defined by (2.2). Then, the following statements hold for $\lambda \in \rho (A_0)$:

(i) $\lambda \in \sigma _{\mathrm{p}}(A_\Theta )$ if and only if $0 \in \sigma _{\mathrm{p}} (\Theta - M(\lambda ))$. Furthermore, one has
  $$\begin{aligned} \ker (A_\Theta - \lambda ) = \bigl \{ \gamma (\lambda ) \varphi : \varphi \in \ker (\Theta - M(\lambda )) \bigr \}. \end{aligned}$$
(ii) If $\lambda \notin \sigma _{\mathrm{p}}(A_\Theta )$ and $\gamma (\overline{\lambda })^* f \in {{\,\mathrm{ran}\,}}(\Theta - M(\lambda ))$, then $f \in {{\,\mathrm{ran}\,}}(A_\Theta - \lambda )$.
(iii) If $\lambda \notin \sigma _{\mathrm{p}}(A_\Theta )$, then
  $$\begin{aligned} (A_\Theta - \lambda )^{-1} f = (A_0 - \lambda )^{-1} f + \gamma (\lambda ) (\Theta - M(\lambda ))^{-1} \gamma (\overline{\lambda })^* f \end{aligned}$$
  for all $$f \in {{\,\mathrm{ran}\,}}(A_\Theta - \lambda )$$.

We point out that assertion (ii) in Theorem 2.3 gives an efficient tool to check the self-adjointness of $A_\Theta$. Since $A_\Theta$ is symmetric by Green’s identity, it suffices to show that ${{\,\mathrm{ran}\,}}(A_\Theta - \lambda _\pm ) = \mathcal {H}$ for some $\lambda _\pm \in \mathbb {C}_\pm$. According to Theorem 2.3 (ii) this is true if ${{\,\mathrm{ran}\,}}\gamma (\overline{\lambda _\pm })^* \subset {{\,\mathrm{ran}\,}}(\Theta - M(\lambda _\pm ))$. Furthermore, if $\lambda \in \rho (A_\Theta )\not =\emptyset$, then the resolvent formula in Theorem 2.3 (iii) holds for all $f\in \mathcal H$, that is, for $\lambda \in \rho (A_\Theta )\cap \rho (A_0)$ we have

$$\begin{aligned} (A_\Theta - \lambda )^{-1} = (A_0 - \lambda )^{-1} + \gamma (\lambda ) (\Theta - M(\lambda ))^{-1} \gamma (\overline{\lambda })^*. \end{aligned}$$

Note that if $\{ \mathcal {G}, \Gamma _0, \Gamma _1 \}$ is a quasi boundary triple for $T \subset S^*$, then Theorem 2.3 shows how the eigenvalues of self-adjoint extensions of *S*, which are contained in $\rho (A_0)$, can be characterized by the Weyl function *M*. If the symmetric operator *S* is simple, then *all* eigenvalues can be characterized with the help of *M*, in particular, also those that are embedded in $\sigma (A_0)$, compare [20, Corollary 3.4]. Note that there are also similar characterizations for the other types of the spectrum available in [20], but in our applications we restrict ourselves to find the eigenvalues.

### Proposition 2.4

Let *S* be a densely defined, closed, symmetric operator in $\mathcal {H}$, let $\{ \mathcal {G}, \Gamma _0, \Gamma _1 \}$ be a quasi boundary triple for $T \subset S^*$, and let *M* be the associated Weyl function. Moreover, let $\Theta$ be a bounded and self-adjoint operator in $\mathcal {G}$ and assume that the associated operator $A_\Theta$ defined by  (2.2) is self-adjoint. Assume, in addition, that *S* is simple. Then, ${{\,\mathrm{ran}\,}}(M(\lambda ) - \Theta )$ is independent of $\lambda \in \mathbb {C} {\setminus } \mathbb {R}$, and $\lambda \in \mathbb {R}$ is an eigenvalue of $A_\Theta$ if and only if there exists $\varphi \in {{\,\mathrm{ran}\,}}(M(\lambda +i \varepsilon ) - \Theta )$ such that

$$\begin{aligned} \lim _{\varepsilon \searrow 0} i \varepsilon \big ( M(\lambda + i \varepsilon ) - \Theta \big )^{-1} \varphi \ne 0. \end{aligned}$$

### Proof

Define the boundary mappings $\Gamma _0^\Theta , \Gamma _1^\Theta : \mathrm {dom}\,T \rightarrow \mathcal {G}$ by

$$\begin{aligned} \Gamma _0^\Theta f := \Gamma _1 f - \Theta \Gamma _0 f \quad \text {and} \quad \Gamma _1^\Theta f = - \Gamma _0 f, \quad f \in \mathrm {dom}\,T. \end{aligned}$$

We claim that $\{ \mathcal {G}, \Gamma _0^\Theta , \Gamma _1^\Theta \}$ is a quasi boundary triple for $T \subset S^*$ with the additional property $T \upharpoonright \ker \Gamma _0^\Theta = A_\Theta$. In fact, using that $\Theta$ is bounded and self-adjoint we deduce from the abstract Green’s identity for $\{ \mathcal {G}, \Gamma _0, \Gamma _1 \}$ and for $f, g \in \mathrm {dom}\,T$ that

$$\begin{aligned}&(T f, g)_{\mathcal {H}} - (f, T g)_{\mathcal {H}}\\&\quad = (\Gamma _1 f, \Gamma _0 g)_{\mathcal {G}} - (\Gamma _0 f, \Gamma _1 g)_{\mathcal {G}} - (\Theta \Gamma _0 f, \Gamma _0 g)_{\mathcal {G}} + (\Gamma _0 f, \Theta \Gamma _0 g)_{\mathcal {G}} \\&\quad = \big (-\Gamma _0 f, (\Gamma _1 - \Theta \Gamma _0) g\big )_{\mathcal {G}} - \big ((\Gamma _1 - \Theta \Gamma _0) f, -\Gamma _0 g\big )_{\mathcal {G}} \\&\quad = \big (\Gamma _1^\Theta f, \Gamma _0^\Theta g\big )_{\mathcal {G}} - \big (\Gamma _0^\Theta f, \Gamma _1^\Theta g\big )_{\mathcal {G}}, \end{aligned}$$

and hence, the abstract Green’s identity holds also for the triple $\{ \mathcal {G}, \Gamma _0^\Theta , \Gamma _1^\Theta \}$.

Next, the definition of $\Gamma _0^\Theta , \Gamma _1^\Theta$ can be written equivalently as

$$\begin{aligned} \begin{pmatrix} \Gamma _0^\Theta \\ \Gamma _1^\Theta \end{pmatrix} = B \begin{pmatrix} \Gamma _0 \\ \Gamma _1 \end{pmatrix}, \qquad B := \begin{pmatrix} -\Theta &{}\quad 1 \\ -1 &{}\quad 0 \end{pmatrix}. \end{aligned}$$

Since $\Theta$ is bounded, it follows that *B* is boundedly invertible with

$$\begin{aligned} B^{-1} = \begin{pmatrix} 0 &{}\quad -1 \\ 1 &{}\quad -\Theta \end{pmatrix}. \end{aligned}$$

Since ${{\,\mathrm{ran}\,}}(\Gamma _0, \Gamma _1)$ is dense in $\mathcal {G} \times \mathcal {G}$, also ${{\,\mathrm{ran}\,}}(\Gamma _0^\Theta , \Gamma _1^\Theta )$ is dense. Finally, the restriction $T \upharpoonright \ker (\Gamma _0^\Theta ) = T \upharpoonright \ker (\Gamma _1 - \Theta \Gamma _0) = A_\Theta$ is self-adjoint by assumption. Therefore, $\{ \mathcal {G}, \Gamma _0^\Theta , \Gamma _1^\Theta \}$ is a quasi boundary triple for $S^*$.

Next, we compute on $\mathbb {C} {\setminus } \mathbb {R}$ the Weyl function $M_\Theta$ corresponding to the triple $\{ \mathcal {G}, \Gamma _0^\Theta , \Gamma _1^\Theta \}$. For a fixed $\lambda \in \mathbb {C} {\setminus } \mathbb {R}$, this is the mapping which is determined uniquely by the relation $M_\Theta (\lambda ) \Gamma _0^\Theta f_\lambda = \Gamma _1^\Theta f_\lambda$ for $f_\lambda \in \ker (T - \lambda )$. For such an $f_\lambda$, we compute

$$\begin{aligned} \Gamma _0^\Theta f_\lambda = ( \Gamma _1 - \Theta \Gamma _0 ) f_\lambda = \big ( M(\lambda ) - \Theta \big ) \Gamma _0 f_\lambda = - \big ( M(\lambda ) - \Theta \big ) \Gamma _1^\Theta f_\lambda . \end{aligned}$$

Note that $M(\lambda ) - \Theta$ is invertible by Theorem 2.3, as otherwise the self-adjoint operator $A_\Theta$ would have the non-real eigenvalue $\lambda$. Thus, we conclude

$$\begin{aligned} M_\Theta (\lambda ) = -(M(\lambda ) - \Theta )^{-1}. \end{aligned}$$

In particular, this implies that $\mathrm {dom}\,M_\Theta (\lambda ) = {{\,\mathrm{ran}\,}}(M(\lambda ) - \Theta ) = {{\,\mathrm{ran}\,}}\Gamma _0^\Theta$ is independent of $\lambda \in \rho (A_\Theta )$.

After all these preparations, the claim of the proposition follows from [20, Corollary 3.4] applied to the quasi boundary triple $\{ \mathcal {G}, \Gamma _0^\Theta , \Gamma _1^\Theta \}$, as *S* is simple.

$$\square $$

Let $\{ \mathcal {G}, \Gamma _0, \Gamma _1 \}$ be a quasi boundary triple for $T \subset S^*$ and let *B* be a symmetric operator in $\mathcal {G}$. In some applications, it is more convenient to consider

2.3 $$\begin{aligned} A_{[B]} := T \upharpoonright \ker (B \Gamma _1 - \Gamma _0) \end{aligned}$$

instead of (2.2). Similarly as above, a symmetric operator *B* leads to a symmetric extension $A_{[B]}$ of *S*, but *B* being self-adjoint does not imply that also $A_{[B]}$ is self-adjoint. But we have the following counterpart of Theorem 2.3, which gives in item (ii) similarly as above an efficient tool to check the self-adjointness of $A_{[B]}$; cf. [19, Theorem 2.6].

### Theorem 2.5

Let *S* be a densely defined, closed, symmetric operator in $\mathcal {H}$, let $\{ \mathcal {G}, \Gamma _0, \Gamma _1 \}$ be a quasi boundary triple for $T \subset S^*$, set $A_0 := T \upharpoonright \ker \Gamma _0$, and let $\gamma$ and *M* be the associated $\gamma$-field and Weyl function, respectively. Moreover, let *B* be a symmetric operator in $\mathcal {G}$ and let the associated operator $A_{[B]}$ be defined by (2.3). Then, the following statements hold for $\lambda \in \rho (A_0)$:

(i) $\lambda \in \sigma _{\mathrm{p}}(A_{[B]})$ if and only if $1 \in \sigma _{\mathrm{p}} (B M(\lambda ))$. Furthermore, one has
  $$\begin{aligned} \ker (A_{[B]} - \lambda ) = \bigl \{ \gamma (\lambda ) \varphi : \varphi \in \ker (I - B M(\lambda )) \bigr \}. \end{aligned}$$
(ii) If $\lambda \notin \sigma _{\mathrm{p}}(A_{[B]})$ and $B \gamma (\overline{\lambda })^* f \in {{\,\mathrm{ran}\,}}( I - B M(\lambda ))$, then $f \in {{\,\mathrm{ran}\,}}(A_{[B]} - \lambda )$.
(iii) If $\lambda \notin \sigma _{\mathrm{p}}(A_{[B]})$, then
  $$\begin{aligned} (A_{[B]} - \lambda )^{-1} f = (A_0 - \lambda )^{-1} f + \gamma (\lambda ) (I - B M(\lambda ))^{-1} B \gamma (\overline{\lambda })^* f \end{aligned}$$
  for all $$f \in {{\,\mathrm{ran}\,}}(A_{[B]} - \lambda )$$.

Note that for $\lambda \in \rho (A_{[B]})\cap \rho (A_0)$ the resolvent formula in Theorem 2.5 (iii) reads as

$$\begin{aligned} (A_{[B]} - \lambda )^{-1} = (A_0 - \lambda )^{-1} + \gamma (\lambda ) (I - B M(\lambda ))^{-1} B \gamma (\overline{\lambda })^*. \end{aligned}$$

Finally, we state the counterpart of Proposition 2.4 for extensions $A_{[B]}$ given by (2.3) to detect all eigenvalues of $A_{[B]}$.

### Proposition 2.6

Let *S* be a densely defined, closed, symmetric operator in $\mathcal {H}$, let $\{ \mathcal {G}, \Gamma _0, \Gamma _1 \}$ be a quasi boundary triple for $T \subset S^*$, and let *M* be the associated Weyl function. Moreover, let *B* be a bounded and self-adjoint operator in $\mathcal {G}$ and assume that the associated operator $A_{[B]}$ defined by  (2.3) is self-adjoint. Assume, in addition, that *S* is simple. Then, ${{\,\mathrm{ran}\,}}(I - B M(\lambda ))$ is independent of $\lambda \in \mathbb {C} {\setminus } \mathbb {R}$, and $\lambda \in \mathbb {R}$ is an eigenvalue of $A_{[B]}$ if and only if there exists $\varphi \in {{\,\mathrm{ran}\,}}(I - B M(\lambda + i \varepsilon ))$ such that

$$\begin{aligned} \lim _{\varepsilon \searrow 0} i \varepsilon M(\lambda + i \varepsilon ) \big ( I - B M(\lambda + i \varepsilon ) \big )^{-1} \varphi \ne 0. \end{aligned}$$

### Proof

The proof is very similar as the one of Proposition 2.4, so we only sketch the main differences here. Define the mappings $\Gamma _0^{[B]}, \Gamma _1^{[B]}: \mathrm {dom}\,T \rightarrow \mathcal {G}$ by

$$\begin{aligned} \Gamma _0^{[B]} f := \Gamma _0 f - B \Gamma _1 f \quad \text {and} \quad \Gamma _1^{[B]} f = \Gamma _1 f, \quad f \in \mathrm {dom}\,T. \end{aligned}$$

Then, one verifies with the same arguments as in the proof of Proposition 2.4 that $\{ \mathcal {G}, \Gamma _0^{[B]}, \Gamma _1^{[B]} \}$ is a quasi boundary triple for $T \subset S^*$ with $T \upharpoonright \Gamma _0^{[B]} = A_{[B]}$.

If we are able to compute the Weyl function $M_{[B]}(\lambda )$ corresponding to the triple $\{ \mathcal {G}, \Gamma _0^{[B]}, \Gamma _1^{[B]} \}$ for $\lambda \in \mathbb {C} {\setminus } \mathbb {R}$, then we can apply again [20, Corollary 3.4] to characterize all eigenvalues of $A_{[B]}$. Let $\lambda \in \mathbb {C} {\setminus } \mathbb {R}$ and $f_\lambda \in \ker (T - \lambda )$ be fixed. Note that $M(\lambda )$ is invertible, as otherwise the symmetric operator $T \upharpoonright \ker \Gamma _1$ would have the non-real eigenvalue $\lambda$, cf. Theorem 2.3 (i). Hence, $M(\lambda ) \Gamma _0 f_\lambda = \Gamma _1 f_\lambda$ implies $\Gamma _0 f_\lambda = M(\lambda )^{-1} \Gamma _1 f_\lambda$, which yields

$$\begin{aligned} \Gamma _0^{[B]} f_\lambda = ( \Gamma _0 - B \Gamma _1 ) f_\lambda = ( I - B M(\lambda ) ) M(\lambda )^{-1} \Gamma _1^{[B]} f_\lambda . \end{aligned}$$

Note that $I - B M(\lambda )$ is invertible by Theorem 2.5, as otherwise the self-adjoint operator $A_{[B]}$ would have the non-real eigenvalue $\lambda$. Thus, we conclude

$$\begin{aligned} M_{[B]}(\lambda ) = M(\lambda ) (I - B M(\lambda ) )^{-1}. \end{aligned}$$

This implies, in particular, that $\mathrm {dom}\,M_{[B]}(\lambda ) = {{\,\mathrm{ran}\,}}(I - B M(\lambda )) = {{\,\mathrm{ran}\,}}\Gamma _0^{[B]}$ is independent of $\lambda \in \rho (A_{[B]})$.

After all these preparations, the claim of the proposition follows from [20, Corollary 3.4] applied to the quasi boundary triple $\{ \mathcal {G}, \Gamma _0^{[B]}, \Gamma _1^{[B]} \}$, as *S* is simple. $\square$

## The Minimal, the Maximal, the MIT Bag, and Some Associated Integral Operators

In this section, we provide some facts on Dirac operators and associated integral operators. First, we collect some properties of the minimal and the maximal realization of the Dirac operator on a domain $\Omega \subset \mathbb {R}^3$. Then, we introduce and discuss the MIT bag operator, which is a distinguished self-adjoint realization of the Dirac operator in $\Omega$, and which serves as a reference operator later. Finally, we introduce several families of integral operators which will play a crucial role in Sects. 4 and  5 in the proofs of the main results of this paper. Throughout this section, let $\Omega$ be a $C^2$-domain in $\mathbb {R}^3$ with compact boundary, that is, $\Omega$ is either a bounded $C^2$-domain or the complement of the closure of such a set. The unit normal vector field at $\partial \Omega$ pointing outwards $\Omega$ is denoted by $\nu$.

### The Minimal and the Maximal Dirac Operator

We are going to study the following two operators acting in $L^2(\Omega ; \mathbb {C}^4)$: The *maximal Dirac operator*

3.1 $$\begin{aligned} T_{\mathrm{max}} f&= -\,i \alpha \cdot \nabla f + m \beta f, \nonumber \\ \quad \mathrm {dom}\,T_{\mathrm{max}}&= \big \{ f \in L^2(\Omega ; \mathbb {C}^4): \alpha \cdot \nabla f \in L^2(\Omega ; \mathbb {C}^4) \big \}, \end{aligned}$$

where the derivatives are understood in the distributional sense, and the *minimal Dirac operator*$T_{\mathrm{min}} = T_{\mathrm{max}} \upharpoonright H^1_0(\Omega ; \mathbb {C}^4)$, which is given in a more explicit form by

3.2 $$\begin{aligned} T_{\mathrm{min}} f = -i \alpha \cdot \nabla f + m \beta f, \quad \mathrm {dom}\,T_{\mathrm{min}} = H^1_0(\Omega ; \mathbb {R}^3). \end{aligned}$$

Some basic and well-known properties of $T_{\mathrm{min}}$ and $T_{\mathrm{max}}$ are collected in the following lemma; cf. [15, Proposition 3.1], [22, Lemma 2.1], and [56, Propositions 2.10 and 2.12].

#### Lemma 3.1

Let $\Omega \subset \mathbb {R}^3$ be a $C^2$-domain with compact boundary and let $T_{\mathrm{max}}$ and $T_{\mathrm{min}}$ be defined by (3.1) and (3.2), respectively. Then, $T_{\mathrm{min}}$ is a densely defined, closed, symmetric operator in $L^2(\Omega ; \mathbb {C}^4)$ and we have

$$\begin{aligned} T_{\mathrm{min}}^* = T_{\mathrm{max}}\qquad \text {and}\qquad T_{\mathrm{min}} = T_{\mathrm{max}}^*. \end{aligned}$$

Moreover, $C^\infty (\overline{\Omega }; \mathbb {C}^4)$ is dense in $\mathrm {dom}\,T_{\mathrm{max}}$ with respect to the graph norm.

Observe that for $f,g\in H^1(\Omega ; \mathbb {C}^4)$, the integration by parts formula

$$\begin{aligned} \big ( \alpha \cdot \nabla f, g \big )_{\Omega } + \big ( f, \alpha \cdot \nabla g \big )_{\Omega } = \big ( (\alpha \cdot \nu ) f|_{\partial \Omega }, g|_{\partial \Omega }\big )_{\partial \Omega } \end{aligned}$$

implies the identity

3.3 $$\begin{aligned} \big ( (-i \alpha \cdot \nabla + m \beta ) f, g \big )_\Omega - \big ( f, (-i \alpha \cdot \nabla + m \beta ) g \big )_\Omega = \big ( - i (\alpha \cdot \nu ) f|_{\partial \Omega }, g|_{\partial \Omega }\big )_{\partial \Omega }.\nonumber \\ \end{aligned}$$

In the next proposition, we verify that $T_\mathrm{min}$ is a simple symmetric operator, that is, there exists no non-trivial invariant subspace for $T_\mathrm{min}$ in $L^2(\Omega ; \mathbb {C}^4)$ on which $T_\mathrm{min}$ reduces to a self-adjoint operator. The simplicity of $T_\mathrm{min}$ is essential in Propositions 5.5 and 5.11 for the characterization of eigenvalues of self-adjoint extensions of $T_\mathrm{min}$ which are embedded in the spectrum of the MIT bag operator.

#### Proposition 3.2

The operator $T_\mathrm{min}$ in (3.2) is simple.

#### Proof

Assume that $T_{\mathrm{min}} = T_1 \oplus T_2$, where $T_j$ acts in an invariant subspace $\mathcal {H}_j \subset L^2(\Omega ; \mathbb {C}^4)$ for $T_{\mathrm{min}}$, $j \in \{ 1, 2\}$, and that $T_1 = T_1^*$. We prove that $\mathcal {H}_1=\{0\}$. For that, note $(T_{\mathrm{min}})^2 = T_1^2 \oplus T_2^2$ and $T_1^2 = (T_1^2)^*$ in $\mathcal {H}_1$ by the spectral theorem. Since $T_1^2$ is closed, we have $\overline{(T_{\mathrm{min}})^2} = T_1^2 \oplus \overline{T_2^2}$. Let us show that $\overline{(T_{\mathrm{min}})^2}$ is simple. We define the operator

$$\begin{aligned} A^\Omega f = (-i \alpha \cdot \nabla + m \beta )^2 f = (-\Delta + m^2) f, \quad \mathrm {dom}\,A^\Omega = H^2(\Omega ; \mathbb {C}^4), \end{aligned}$$

and we claim that $A^\Omega \subset ( (T_{\mathrm{min}})^2 )^*$. In fact, consider arbitrary $f \in \mathrm {dom}\,A^\Omega$ and let $g \in \mathrm {dom}\,(T_{\mathrm{min}})^2$. Then, $g, (-i \alpha \cdot \nabla + m \beta ) g \in H^1_0(\Omega ; \mathbb {C}^4)$ and the identity (3.3) shows

$$\begin{aligned} \big ( A^\Omega f, g \big )_\Omega&= \big ( (-i \alpha \cdot \nabla + m \beta )^2 f, g \big )_\Omega \\&= \big ( (-i \alpha \cdot \nabla + m \beta ) f, (-i \alpha \cdot \nabla + m \beta ) g \big )_\Omega \\&= \big ( f, (-i \alpha \cdot \nabla + m \beta )^2 g \big )_\Omega \\&= \big ( f, (T_{\mathrm{min}})^2 g \big )_\Omega , \end{aligned}$$

which implies $A^\Omega \subset ( (T_{\mathrm{min}})^2 )^*$. Now we use that

$$\begin{aligned} L^2(\Omega ; \mathbb {C}^4)=\overline{\text {span} \big \{ \ker (A^\Omega - \lambda ) : \lambda \in \mathbb {C} {\setminus } \mathbb {R} \big \} }; \end{aligned}$$

if $\Omega$ is bounded, this is essentially a consequence of unique continuation (for details see [14, Section 8.3]) and if $\Omega$ is unbounded this fact can be found in [21, Proposition 2.2]. As $A^\Omega \subset ( (T_{\mathrm{min}})^2 )^*$ and $( (T_{\mathrm{min}})^2 )^*=(\overline{(T_{\mathrm{min}})^2})^*$, we have

$$\begin{aligned} \ker (A^\Omega - \lambda ) \subset \ker \bigl (\bigl (\,\overline{(T_{\mathrm{min}})^2}\,\bigr )^*-\lambda \bigr ),\quad \lambda \in \mathbb {C} {\setminus } \mathbb {R}, \end{aligned}$$

and we conclude

$$\begin{aligned} L^2(\Omega ; \mathbb {C}^4)=\overline{\text {span} \big \{ \ker \bigl (\bigl (\,\overline{(T_{\mathrm{min}})^2}\,\bigr )^* - \lambda \bigr ) : \lambda \in \mathbb {C} {\setminus } \mathbb {R} \big \} }. \end{aligned}$$

This implies that $\overline{(T_{\mathrm{min}})^2}$ is simple (see (1.10)). Therefore, $\mathcal {H}_1=\{0\}$ and hence $T_{\mathrm{min}}$ is simple. $\square$

### The MIT Bag Operator

In this subsection, we discuss the MIT bag Dirac operator in $\Omega$ which will often play the role of a self-adjoint reference operator in this paper. The MIT bag operator is the partial differential operator in $L^2(\Omega ; \mathbb {C}^4)$ defined by

3.4 $$\begin{aligned} T_{\mathrm{MIT}} f&= (-i \alpha \cdot \nabla + m \beta ) f, \nonumber \\ \mathrm {dom}\,T_{\mathrm{MIT}}&= \bigl \{ f \in H^1(\Omega ; \mathbb {C}^4): f|_{\partial \Omega } = -i \beta (\alpha \cdot \nu ) f|_{\partial \Omega } \bigr \}. \end{aligned}$$

In the following proposition, we summarize the basic properties of $T_{\mathrm{MIT}}$. For some further results on $T_\text {MIT}$, as, e.g., symmetry relations of the spectrum or asymptotics of eigenvalues for large masses *m* we refer to [2]. Moreover, we note that the orthogonal sum of the MIT bag operator in $\Omega$ and $\mathbb R^3{\setminus }\overline{\Omega }$ is a Dirac operator with a Lorentz scalar $\delta$–shell interaction, see Proposition 5.15. Using this, one can show even some further properties of $T_\text {MIT}$; cf. (5.33).

#### Proposition 3.3

The operator $T_{\mathrm{MIT}}$ defined by (3.4) is self-adjoint in $L^2(\Omega ; \mathbb {C}^4)$ and the following statements hold:

(i) $$(-m, m) \subset \rho (T_{\mathrm{MIT}})$$.
(ii) If $\Omega$ is bounded, then $\sigma (T_{\mathrm{MIT}}) = \sigma _{\mathrm{disc}}(T_{\mathrm{MIT}})=\sigma _\mathrm{p}(T_{\mathrm{MIT}})$.
(iii) If $\Omega$ is unbounded, then $\sigma (T_{\mathrm{MIT}}) = \sigma _{\mathrm{ess}}(T_{\mathrm{MIT}}) = (-\infty , - m] \cup [m, \infty )$.

#### Proof

First, the self-adjointness of $T_{\mathrm{MIT}}$ is shown in [56, Theorem 3.2]. The proof of assertion (i) follows similar considerations in [2, Theorem 1.5] for $C^3$-domains, but the arguments are basically independent of the smoothness of $\partial \Omega$. Indeed, one can show for $f \in \mathrm {dom}\,T_{\mathrm{MIT}}$ with the help of (3.3) and (1.8) that

$$\begin{aligned} \Vert T_{\mathrm{MIT}} f \Vert ^2_\Omega&= \big ( (-i \alpha \cdot \nabla + m \beta ) f, (-i \alpha \cdot \nabla + m \beta ) f \big )_\Omega \\&= \Vert \alpha \cdot \nabla f \Vert ^2_\Omega + m^2 \Vert f \Vert _\Omega ^2 + ( -i \alpha \cdot \nabla f, m \beta f )_\Omega + (m \beta f, -i \alpha \cdot \nabla f)_\Omega \\&= \Vert \alpha \cdot \nabla f \Vert ^2_\Omega + m^2 \Vert f \Vert _\Omega ^2 + m \big ( -i \beta (\alpha \cdot \nu ) f|_{\partial \Omega }, f|_{\partial \Omega } \big )_{\partial \Omega } \\&=\Vert \alpha \cdot \nabla f \Vert _\Omega ^2 + m^2 \Vert f \Vert _\Omega ^2 + m \Vert f |_{\partial \Omega } \Vert _{\partial \Omega }^2 \end{aligned}$$

holds, where the boundary condition for $f \in \mathrm {dom}\,T_\text {MIT}$ was used in the last step. Hence, we have $\Vert T_{\mathrm{MIT}} f \Vert _\Omega \ge m \Vert f \Vert _\Omega$ for all $f \in \mathrm {dom}\,T_{\mathrm{MIT}}$, which shows that $\sigma (T_{\mathrm{MIT}}) \cap (-m, m) = \emptyset$.

To verify item (ii), we note that $\mathrm {dom}\,T_{\mathrm{MIT}} \subset H^1(\Omega ; \mathbb {C}^4)$ is compactly embedded in $L^2(\Omega ; \mathbb {C}^4)$, as $\Omega$ is a bounded $C^2$-domain. Hence, $\sigma (T_{\mathrm{MIT}})$ is purely discrete.

It remains to show point (iii). By (i), we have $\sigma (T_{\mathrm{MIT}}) \subset (-\infty , -m] \cup [m, \infty )$. To prove the other inclusion, fix some $\lambda \in (-\infty , -m] \cup [m, \infty )$, a number $R>0$ such that $\mathbb {R}^3 {\setminus } B(0, R) \subset \Omega$, a vector $\zeta \in \mathbb {C}^4$ such that $\left( \sqrt{\lambda ^2 - m^2} \alpha _1 + m \beta + \lambda I_4 \right) \zeta \ne 0$, a cutoff-function $\chi \in C^\infty _0(\mathbb {R})$ with $\chi (r) = 1$ for $r < \frac{1}{2}$ and $\chi (r) = 0$ for $r > 1$ and set $x_n := (R + n^2, 0, 0)^\top$, $n \in \mathbb {N}$. Then, we define the function $\psi _n^\lambda$ by

$$\begin{aligned} \psi _n^\lambda (x) := \frac{1}{n^{3/2}} \chi \left( \frac{1}{n} |x - x_n| \right) e^{i \sqrt{\lambda ^2 - m^2} x\cdot e_1} \left( \sqrt{\lambda ^2 - m^2} \alpha _1 + m \beta + \lambda I_4 \right) \zeta . \end{aligned}$$

Then, one verifies in the same way as in [15, Theorem 5.7] that $\psi _n^\lambda \in \mathrm {dom}\,T_{\mathrm{MIT}}$, that $\psi _n^\lambda$ converge weakly to zero, that

$$\begin{aligned} \Vert \psi _n^\lambda \Vert _{\Omega } = \text {const.} > 0 \quad \text {and} \quad (T_{\mathrm{MIT}}^{\Omega } - \lambda ) \psi _n^\lambda \rightarrow 0, \quad \text {as } n \rightarrow \infty . \end{aligned}$$

Thus, $(\psi _n^\lambda )_n$ is a singular sequence for $T_{\mathrm{MIT}}$ and $\lambda$, which shows $\lambda \in \sigma _{\mathrm{ess}}(T_{\mathrm{MIT}})$. This finishes the proof of this proposition. $\square$

In a similar fashion as for the MIT bag model, we also state some basic properties of another distinguished self-adjoint realization of the Dirac operator on $\Omega$. This operator has similar boundary conditions as $T_{\mathrm{MIT}}$, but with opposite sign, and is given by

3.5 $$\begin{aligned} T_{-\mathrm {MIT}} f&= (-i \alpha \cdot \nabla + m \beta ) f, \nonumber \\ {\mathrm {dom}}\,T_{-\mathrm {MIT}}&= \bigl \{ f \in H^1(\Omega ; {\mathbb {C}}^4): f|_{\partial \Omega } = i \beta (\alpha \cdot \nu ) f|_{\partial \Omega } \bigr \}. \end{aligned}$$

The next proposition is the counterpart of Proposition 3.3 for $T_{-\mathrm{MIT}}$. However, in contrast to Proposition 3.3 (i) the interval $(-m, m)$ may also contain spectrum; cf. Theorem 5.4 and Proposition 5.5.

#### Proposition 3.4

The operator $T_{-\mathrm{MIT}}$ defined by (3.5) is self-adjoint in $L^2(\Omega ; \mathbb {C}^4)$, and the following statements hold:

(i) If $\Omega$ is bounded, then $\sigma (T_{-\mathrm {MIT}}) = \sigma _{\mathrm {disc}}(T_{-{\mathrm {MIT}}})=\sigma _{\mathrm {p}}(T_{-{\mathrm {MIT}}})$.
(ii) If $\Omega$ is unbounded, then $(-\infty , - m] \cup [m, \infty ) \subset \sigma _{\mathrm {ess}}(T_{-\mathrm {MIT}})$.

#### Remark 3.5

We will show later in Theorem 5.4 (i) that the inclusion in item (ii) of the above proposition is in fact an equality, i.e.,

$$\begin{aligned} \sigma _{\mathrm {ess}}(T_{-{\mathrm {MIT}}}) = (-\infty , - m] \cup [m, \infty ). \end{aligned}$$

This holds, as the operator $T_{-\mathrm{MIT}}$ corresponds to $T \upharpoonright \ker \Gamma _1$ defined as in (5.2) with the parameter $\vartheta = 0$. But for our next considerations, the above inclusion is sufficient.

#### Proof of Proposition 3.4

Define the auxiliary operator

$$\begin{aligned} \widetilde{T} f = (-i \alpha \cdot \nabla - m \beta ) f, \quad \mathrm {dom}\,\widetilde{T} = \bigl \{ f \in H^1(\Omega ; \mathbb {C}^4): f|_{\partial \Omega } = i \beta (\alpha \cdot \nu ) f|_{\partial \Omega } \bigr \}, \end{aligned}$$

and consider the unitary and self-adjoint matrix

$$\begin{aligned} \gamma _5 = \begin{pmatrix} 0 &{}\quad I_2 \\ I_2 &{}\quad 0 \end{pmatrix}. \end{aligned}$$

We claim that

3.6 $$\begin{aligned} T_\text {MIT} = \gamma _5 \widetilde{T} \gamma _5. \end{aligned}$$

To see this, we note that $\gamma _5 \beta = -\beta \gamma _5$ and $(\alpha \cdot x) \gamma _5 = \gamma _5 (\alpha \cdot x)$ for all $x \in \mathbb {R}^3$. This implies

$$\begin{aligned} (-i \alpha \cdot \nabla + m \beta ) f = \gamma _5 (-i \alpha \cdot \nabla - m \beta )\gamma _5 f,\quad f \in H^1(\Omega ; \mathbb {C}^4). \end{aligned}$$

Furthermore, we have $f \in \mathrm {dom}\,T_\text {MIT}$ if and only if $\gamma _5 f \in H^1(\Omega ; \mathbb {C}^4)$ and

$$\begin{aligned} i \beta (\alpha \cdot \nu ) \gamma _5 f|_{\partial \Omega } = -\gamma _5 i \beta (\alpha \cdot \nu ) f|_{\partial \Omega } = \gamma _5 f|_{\partial \Omega }, \end{aligned}$$

so that $f \in \mathrm {dom}\,T_\text {MIT}$ if and only if $\gamma _5 f\in \mathrm {dom}\,\widetilde{T}$. Hence, we have shown (3.6). In particular, this implies together with Proposition 3.3 that $\widetilde{T}$ is self-adjoint. Since multiplication by $m \beta$ is a bounded and self-adjoint operator in $L^2(\Omega ; \mathbb {C}^4)$, we conclude that also $T_{-\text {MIT}} = \widetilde{T} + 2 m \beta$ is self-adjoint.

Eventually, assertions (i) and (ii) can be shown in exactly the same way as Proposition 3.3 (ii) and (iii); in particular, for the proof of item (ii) the same singular sequence as in Proposition 3.3 (iii) can be used. $\square$

### Integral Operators

In this section, we introduce several families of integral operators that will play an important role in the analysis of Dirac operators on domains. We also summarize some of their well-known properties. Define for $\lambda \in \mathbb {C} {\setminus } ( (-\infty , -m] \cup [m, \infty ) )$ the function

3.7 $$\begin{aligned} G_\lambda (x) = \left( \lambda I_4 + m \beta + \left( 1 - i \sqrt{\lambda ^2 - m^2} |x| \right) \frac{i}{|x|^2} (\alpha \cdot x) \right) \cdot \frac{e^{i \sqrt{\lambda ^2 - m^2} |x|}}{4 \pi |x|}.\nonumber \\ \end{aligned}$$

Recall that $G_\lambda$ is the integral kernel of the resolvent of the free Dirac operator in $\mathbb {R}^3$, see [63, Section 1.E]. We introduce the families of integral operators $\Phi _\lambda : L^2({\partial \Omega }; \mathbb {C}^4) \rightarrow L^2(\Omega ; \mathbb {C}^4)$,

3.8 $$\begin{aligned} \Phi _\lambda \varphi (x) = \int _{{\partial \Omega }} G_\lambda (x - y) \varphi (y) \mathrm {d} \sigma (y), \quad x \in \Omega ,\, \varphi \in L^2({\partial \Omega }; \mathbb {C}^4), \end{aligned}$$

and $$\mathcal {C}_\lambda : L^2({\partial \Omega }; \mathbb {C}^4) \rightarrow L^2({\partial \Omega }; \mathbb {C}^4),$$

3.9 $$\begin{aligned} \mathcal {C}_\lambda \varphi (x) = \lim _{\varepsilon \searrow 0 }\int _{{\partial \Omega } {\setminus } B(x, \varepsilon )} G_\lambda (x - y) \varphi (y) \mathrm {d} \sigma (y), \quad x \in {\partial \Omega },\, \varphi \in L^2({\partial \Omega }; \mathbb {C}^4). \end{aligned}$$

It is well known that $\Phi _\lambda$ and $\mathcal {C}_\lambda$ are bounded and everywhere defined and that

3.10 $$\begin{aligned} \mathcal {C}_\lambda ^* = \mathcal {C}_{\overline{\lambda }} \end{aligned}$$

holds; cf. [4, Lemmas 2.1 and 3.3] or [10, Proposition 3.4]. Furthermore, the adjoint of $\Phi _\lambda$ is given by $\Phi _\lambda ^*: L^2(\Omega ; \mathbb {C}^4) \rightarrow L^2({\partial \Omega }; \mathbb {C}^4),$

3.11 $$\begin{aligned} \Phi _\lambda ^* f(x) = \int _{\Omega } G_{\overline{\lambda }}(x-y) f(y) \mathrm {d} y, \quad x \in {\partial \Omega },\, f \in L^2(\Omega ; \mathbb {C}^4), \end{aligned}$$

and this operator is also bounded when viewed as an operator from $L^2(\Omega ; \mathbb {C}^4)$ to $H^{1/2}(\partial \Omega ; \mathbb {C}^4)$; cf. [12, equation (2.12) and the discussion below]. Hence, we can define the anti-dual of $\Phi _\lambda ^*$ by

3.12 $$\begin{aligned} \Phi _{\lambda , -1/2} := (\Phi _\lambda ^*)': H^{-1/2}(\partial \Omega ; \mathbb {C}^4) \rightarrow L^2(\Omega ; \mathbb {C}^4). \end{aligned}$$

Since we have for $\varphi \in L^2(\partial \Omega ; \mathbb {C}^4)$ and $f \in L^2(\Omega ; \mathbb {C}^4)$

$$\begin{aligned} (\Phi _{\lambda , -1/2} \varphi , f)_\Omega&= (\varphi , \Phi _\lambda ^* f)_{H^{-1/2}(\partial \Omega ; \mathbb {C}^4) \times H^{1/2}(\partial \Omega ; \mathbb {C}^4)} \\&= (\varphi , \Phi _\lambda ^* f)_{\partial \Omega } = (\Phi _{\lambda } \varphi , f)_\Omega , \end{aligned}$$

$\Phi _{\lambda , -1/2}$ is an extension of $\Phi _\lambda$. Some further properties of $\Phi _\lambda$ are summarized in the following proposition.

#### Proposition 3.6

Let $\lambda \in \mathbb {C} {\setminus } ( (-\infty , -m] \cup [m, \infty ))$ and let $\Phi _\lambda$ be the operator in (3.8). Then, the following statements hold:

(i) For any $s \in [-\frac{1}{2}, \frac{1}{2} ]$ the operator $\Phi _\lambda$ gives rise to a bounded and everywhere defined operator $\Phi _{\lambda , s}: H^s(\partial \Omega ; \mathbb {C}^4) \rightarrow H^{s+1/2}(\Omega ; \mathbb {C}^4)$.
(ii) $${{\,\mathrm{ran}\,}}\Phi _{\lambda , -1/2} = \ker (T_\mathrm{max} - \lambda )$$.

#### Remark 3.7

For $s \in (-\frac{1}{2}, \frac{1}{2}]$ the map $\Phi _{\lambda , s}$ is the restriction of $\Phi _{\lambda , -1/2}$ onto $H^s(\partial \Omega ; \mathbb {C}^4)$, i.e., $\Phi _{\lambda , s} \varphi$ is for $\varphi \in H^s(\partial \Omega ; \mathbb {C}^4)$ the uniquely determined $L^2$-function $\Phi _{\lambda , -1/2} \varphi$.

#### Proof of Proposition 3.6

The claim of statement (i) for $s=-\frac{1}{2}$ follows from the definition of $\Phi _{\lambda , -1/2}$ in (3.12), and it is contained for $s = \frac{1}{2}$ and $\Omega = \mathbb {R}^3 {\setminus } \Sigma$ for a $C^2$-surface $\Sigma$ in [15, Proposition 4.2] (see also Remark 3.7); for that, one has to note that the map $\gamma (\lambda )$ in [15] coincides with $\Phi _{\lambda , 1/2}$. The claim for $\Omega$ follows from this by restriction and for intermediate $s \in ( -\frac{1}{2}, \frac{1}{2})$ by an interpolation argument. Assertion (ii) follows immediately from [15, Propositions 4.4 and 2.6] by noting that $\Phi _{\lambda , -1/2}$ coincides with $\widetilde{\gamma }(\lambda )$ in [15].

$$\square $$

In the next proposition we collect some additional properties of $\mathcal {C}_\lambda$ that will be useful in the sequel.

#### Proposition 3.8

Let $\lambda \in \mathbb {C} {\setminus } ( (-\infty , -m] \cup [m, \infty ) )$ and let $\mathcal {C}_\lambda$ be the operator in (3.9). Then, the following statements hold:

(i) For any $s \in [-\frac{1}{2}, \frac{1}{2} ]$ the operator $\mathcal {C}_\lambda$ gives rise to a bounded and everywhere defined operator $\mathcal {C}_{\lambda , s}: H^s(\partial \Omega ; \mathbb {C}^4) \rightarrow H^s(\partial \Omega ; \mathbb {C}^4)$ and for the anti-dual of $\mathcal {C}_{\lambda , s}$ one has $\mathcal {C}_{\lambda , s}' = \mathcal {C}_{\overline{\lambda }, -s}$.
(ii) If $\lambda \in (-m, m)$ and $s \in [-\frac{1}{2}, \frac{1}{2} ]$, then the operator $\mathcal {C}_{\lambda , s}$ is invertible and
  3.13 $$\begin{aligned} -4 \bigl (\mathcal {C}_{\lambda , s} (\alpha \cdot \nu )\bigr )^2 = -4 \bigl ((\alpha \cdot \nu ) \mathcal {C}_{\lambda , s}\bigr )^2 = I_4. \end{aligned}$$

#### Proof

First, by [15, Proposition 4.2] the restriction $\mathcal {C}_{\lambda ,1/2} := \mathcal {C}_\lambda \upharpoonright H^{1/2}(\partial \Omega ; \mathbb {C}^4)$ is bounded in $H^{1/2}(\partial \Omega ; \mathbb {C}^4)$. Moreover, by [15, Proposition 4.4] the mapping $\mathcal {C}_{\lambda , 1/2}$ can be extended by continuity to a bounded operator $\mathcal {C}_{\lambda , -1/2}$ in $H^{-1/2}(\partial \Omega ; \mathbb {C}^4)$ and it is also shown there that

$$\begin{aligned} (\mathcal {C}_{\lambda , 1/2} \varphi , \psi )_{H^{1/2}(\partial \Omega ; \mathbb {C}^4) \times H^{-1/2}(\partial \Omega ; \mathbb {C}^4)} = (\varphi , \mathcal {C}_{\overline{\lambda }, -1/2} \psi )_{H^{1/2}(\partial \Omega ; \mathbb {C}^4) \times H^{-1/2}(\partial \Omega ; \mathbb {C}^4)} \end{aligned}$$

holds for all $\varphi \in H^{1/2}(\partial \Omega ; \mathbb {C}^4)$ and $\psi \in H^{-1/2}(\partial \Omega ; \mathbb {C}^4)$; for this, one just has to note that the operators $M(\lambda )$ and $\widetilde{M}(\lambda )$ in [15] coincide with $\mathcal {C}_{\lambda , 1/2}$ and $\mathcal {C}_{\lambda , -1/2}$, respectively. Hence, assertion (i) holds for $s=\pm \frac{1}{2}$. Now the continuity claim in item (i) for the restriction $\mathcal {C}_{\lambda , s} := \mathcal {C}_{\lambda , -1/2} \upharpoonright H^s(\partial \Omega ; \mathbb {C}^4)$ for intermediate $s \in ( -\frac{1}{2}, \frac{1}{2} )$ follows via interpolation. Moreover, for $s \in (-\frac{1}{2}, 0)$ the map $\mathcal {C}_{\lambda , s}$ is the anti-dual of $\mathcal {C}_{\overline{\lambda }, -s}$. To see the last claim, we note that for $\varphi \in L^2(\partial \Omega ; \mathbb {C}^4)$ and $\psi \in H^{-s}(\partial \Omega ; \mathbb {C}^4)$, one has

$$\begin{aligned} (\mathcal {C}_{\lambda , s} \varphi , \psi )_{H^{s}(\partial \Omega ; \mathbb {C}^4) \times H^{-s}(\partial \Omega ; \mathbb {C}^4)}&= (\mathcal {C}_{\lambda } \varphi , \psi )_{\partial \Omega } = (\varphi , \mathcal {C}_{\overline{\lambda }} \psi )_{\partial \Omega }\\&= (\varphi , \mathcal {C}_{\overline{\lambda }, -s} \psi )_{H^{s}(\partial \Omega ; \mathbb {C}^4) \times H^{-s}(\partial \Omega ; \mathbb {C}^4)}, \end{aligned}$$

where (3.10) was used. By density, this can be extended for all $\varphi \in H^s(\partial \Omega ; \mathbb {C}^4)$, which implies that indeed $\mathcal {C}_{\lambda , s}' = \mathcal {C}_{\overline{\lambda }, -s}$.

Identity (3.13) in (ii) is shown for $\lambda = s = 0$ in [4, Lemma 3.3] and can be shown for $\lambda \ne 0$ and $s=0$ in a similar way. Clearly, this also implies the invertibility of $\mathcal {C}_{\lambda }$. For $s \in (0, \frac{1}{2}]$, the claim follows from $\mathcal {C}_{\lambda , s} = \mathcal {C}_\lambda \upharpoonright H^s(\partial \Omega ; \mathbb {C}^4)$. This and $\mathcal {C}_{\lambda , s}' = \mathcal {C}_{\overline{\lambda }, -s}$ imply (3.13) also for $s \in [-\frac{1}{2}, 0)$. $\square$

For $\varphi \in H^{1/2}({\partial \Omega }; \mathbb {C}^4)$, the trace of $\Phi _{\lambda ,1/2} \varphi$ and the function $\mathcal {C}_{\lambda ,1/2} \varphi$ are closely related. The formula in the next lemma will be useful in the next sections.

#### Lemma 3.9

Let $\lambda \in \mathbb {C} {\setminus } ( (-\infty , -m] \cup [m, \infty ))$ and let $\Phi _{\lambda ,1/2}$ and $\mathcal {C}_{\lambda ,1/2}$ be the operators in Proposition 3.6 and Proposition 3.8, respectively. Then, one has

$$\begin{aligned} (\Phi _{\lambda ,1/2} \varphi )\vert _{\partial \Omega } = \mathcal {C}_{\lambda ,1/2} \varphi - \frac{i}{2} (\alpha \cdot \nu ) \varphi , \qquad \varphi \in H^{1/2}({\partial \Omega }; \mathbb {C}^4). \end{aligned}$$

#### Proof

The formula is shown for $\lambda = 0$ in [4, Lemma 3.3] in terms of the non-tangential limit of $\Phi _{\lambda ,1/2} \varphi \in H^{1}(\Omega ; \mathbb {C}^4)$. Since the non-tangential limit and the trace coincide (see, e.g., [13, Lemma 3.1]), the claim follows for $\lambda = 0$; the claim for general $\lambda$ can be deduced in the same way. $\square$

In the following proposition, we show that the commutator of the singular integral operator $\mathcal {C}_{\lambda }$ and a Hölder continuous function of order $a> 0$ is bounded from $L^2(\partial \Omega ; \mathbb {C}^4)$ to $H^s(\partial \Omega ; \mathbb {C}^4)$, $s \in [0, a)$, and hence compact in $H^s(\partial \Omega ; \mathbb {C}^4)$ for $a>s\ge 0$. This has important consequences for the analysis of self-adjoint Dirac operators on domains and will be used in the proofs of many of the main results in this paper. The proof of the next result relies on the properties of integral operators established in Appendix A.

#### Proposition 3.10

Let $\lambda \in \mathbb {C} {\setminus } ( (-\infty , - m] \cup [m, \infty ) )$, let $\mathcal {C}_\lambda$ be the operator in (3.9), and assume that $\vartheta \in \mathrm {Lip}_a({\partial \Omega })$ for some $a\in (0,1]$. Then, the commutator

$$\begin{aligned} \varphi \mapsto \mathcal {C}_{\lambda }(\vartheta \varphi ) -\vartheta \,\mathcal {C}_{\lambda }\varphi \end{aligned}$$

is bounded from $L^2(\partial \Omega ; \mathbb {C}^4)$ to $H^{s}(\partial \Omega ; \mathbb {C}^4)$ for any $s \in [0, a)$. In particular, the restriction $\mathcal {C}_{\lambda , s} \vartheta -\vartheta \,\mathcal {C}_{\lambda , s}$ is compact in $H^{s}(\partial \Omega ; \mathbb {C}^4)$ for every $s \in [0, a)$.

#### Proof

To prove the claimed mapping properties we show that each component $k_{j l}$, $j, l\in \{1,\dots ,4\}$, of the matrix-valued integral kernel

$$\begin{aligned} K(x, y) := G_\lambda (x-y) (\vartheta (y) - \vartheta (x)) \end{aligned}$$

of $\mathcal {C}_{\lambda } \vartheta - \vartheta \mathcal {C}_{\lambda }$ satisfies the estimates in (A.4). As $a>s$ the claim of this proposition follows from Theorem A.3 applied to the scalar integral operators with integral kernels $k_{j l}$, $j, l\in \{1,\dots ,4\}$. Since the embedding $\iota _s: H^s(\partial \Omega ; \mathbb {C}^4) \rightarrow L^2(\partial \Omega ; \mathbb {C}^4)$ is compact for $s\in (0,a)$, this implies then also the compactness of $\mathcal {C}_{\lambda , s} \vartheta - \vartheta \mathcal {C}_{\lambda , s}$ in $H^s(\partial \Omega ; \mathbb {C}^4)$. Indeed, for $s > 0$ it follows that $\mathcal {C}_{\lambda , s} \vartheta - \vartheta \mathcal {C}_{\lambda , s} = (\mathcal {C}_{\lambda } \vartheta - \vartheta \mathcal {C}_{\lambda }) \iota _s$ is compact in $H^s(\partial \Omega ; \mathbb {C}^4)$, and for $s=0$, one can choose $r \in (0,a)$ and sees with the result of this proposition that $\mathcal {C}_{\lambda } \vartheta - \vartheta \mathcal {C}_{\lambda }: L^2(\partial \Omega ; \mathbb {C}^4) \rightarrow H^r(\partial \Omega ; \mathbb {C}^4)$ is bounded, and hence $\mathcal {C}_{\lambda } \vartheta - \vartheta \mathcal {C}_{\lambda } = \iota _r (\mathcal {C}_{\lambda } \vartheta - \vartheta \mathcal {C}_{\lambda })$ is compact in $L^2(\partial \Omega ; \mathbb {C}^4)$.

In the sequel, we use the matrix norm

$$\begin{aligned} |M| := \max _{1 \le k, l \le 4} |m_{k l}|,\qquad M=(m_{kl})_{k,l=1}^4 \in \mathbb {C}^{4 \times 4}. \end{aligned}$$

Throughout the proof of this proposition, *C* is a generic constant with different values on different places. First, due to the Hölder continuity of $\vartheta$ we conclude immediately from the definition of $G_\lambda$ in (3.7) that

$$\begin{aligned} |K(x, y)| = |G_\lambda (x-y)| \cdot |\vartheta (y) - \vartheta (x)| \le C \frac{|y-x|^a }{|x - y|^2} = \frac{C}{|x - y|^{2-a}} \end{aligned}$$

holds for all $x,y\in \partial \Omega$, $x \ne y$. Hence, the first estimate in (A.4) is satisfied. To show the second one, we take $x, y, z \in \partial \Omega$ with $|x - y| \le \frac{1}{4} |x - z|$, write

3.14 $$\begin{aligned}&K(x, \,z) - K(y, z) \nonumber \\&\quad = G_\lambda (x - z) (\vartheta (y) - \vartheta (x)) + \big ( G_\lambda (x-z) - G_\lambda (y-z) \big ) (\vartheta (z) - \vartheta (y)), \end{aligned}$$

and show that each of the two terms on the right-hand side of (3.14) fulfills this growth condition. Clearly, using the Hölder continuity of $\vartheta$ and the definition of $G_\lambda$ from (3.7) we find first that

3.15 $$\begin{aligned} \big | G_\lambda (x - z) (\vartheta (y) - \vartheta (x)) \big | \le C \frac{|x - y|^a}{|x - z|^2}. \end{aligned}$$

To get an estimate for the second term in (3.14), we note first that the Hölder continuity of $\vartheta$, the triangle inequality, and $|x - y| \le \frac{1}{4} |x - z|$ yield

3.16 $$\begin{aligned} |\vartheta (z) - \vartheta (y)| \le C |y - z|^a \le C \big ( |x - y| + |x -z| \big )^a \le C |x - z|^a. \end{aligned}$$

In the next calculation, we use for $\xi , \zeta \in \mathbb {R}^3 {\setminus } \{ 0 \}$ the notation

$$\begin{aligned} \nabla G_\lambda (\xi ) \cdot \zeta := \partial _1 G_\lambda (\xi ) \cdot \zeta _1 + \partial _2 G_\lambda (\xi ) \cdot \zeta _2 + \partial _3 G_\lambda (\xi ) \cdot \zeta _3. \end{aligned}$$

Then, we deduce with the main theorem of calculus applied to each entry of the matrix $G_\lambda (x-z) - G_\lambda (y-z)$

3.17 $$\begin{aligned} \big | G_\lambda (x-z) - G_\lambda (y-z) \big |&= \left| \int _0^1 \frac{\text {d}}{\text {d} t} G_\lambda (t (x - y) + y - z) \text {d} t \right| \nonumber \\&\le \int _0^1 \big | \nabla G_\lambda (t (x - y) + y - z) \cdot (x - y) \big | \text {d} t. \end{aligned}$$

Using (3.7), we find

$$\begin{aligned}&\partial _j G_\lambda (x)\\ {}&\quad = \Bigg [\left( \lambda I_4 + m \beta + \left( 1 - i \sqrt{\lambda ^2 - m^2} |x| \right) \frac{i(\alpha \cdot x )}{|x|^2} \right) \left( i \sqrt{\lambda ^2 - m^2} - \frac{1}{|x|} \right) \partial _j |x| \\ {}&\quad \qquad +\,\left( 1 - i \sqrt{\lambda ^2 - m^2} |x| \right) \frac{i}{|x|^2} \left( \alpha _j - \frac{2 \alpha \cdot x}{|x|} \partial _j |x| \right) \\ {}&\quad \qquad +\, \sqrt{\lambda ^2 - m^2} \, \frac{\alpha \cdot x}{|x|^2}\, \partial _j |x| \Bigg ] \frac{e^{i \sqrt{\lambda ^2 - m^2} |x|}}{4 \pi |x|}, \end{aligned}$$

which implies $|\partial _j G_\lambda (x)| \le C |x|^{-3}$. Substituting this in (3.17) yields

3.18 $$\begin{aligned} \big | G_\lambda (x-z) - G_\lambda (y-z) \big | \le C \int _0^1 |t (x - y) + y - z|^{-3} \text {d} t \cdot |x - y|. \end{aligned}$$

Since $|x - y| \le \frac{1}{4} |x - z|$, we can estimate for $t \in [0, 1]$

$$\begin{aligned} |t (x - y) + y - z| \ge |y - z| - t |x - y| \ge |x - z| - (1 + t) |x - y| \ge \frac{1}{2} |x - z| \end{aligned}$$

and

$$\begin{aligned} |x - y| = |x - y|^a \cdot |x - y|^{1-a} \le C |x - y|^a \cdot |x - z|^{1-a}. \end{aligned}$$

Using these two observations in (3.18), we conclude

$$\begin{aligned} \big | G_\lambda (x-z) - G_\lambda (y-z) \big | \le C \frac{|x - y|^a \cdot |x - z|^{1-a}}{|x - z|^3} = C \frac{|x - y|^a}{|x - z|^{2 + a}}. \end{aligned}$$

Together with (3.16), this leads to

$$\begin{aligned} \big | \big (G_\lambda (x-z) - G_\lambda (y-z) \big ) (\vartheta (z) - \vartheta (y)) \big | \le C \frac{|x - y|^a}{|x - z|^2}. \end{aligned}$$

It follows now easily from (3.14), the triangle inequality, (3.15), and the last estimate that the components of *K* also satisfy the second condition in (A.4). This completes the proof of this proposition. $\square$

We now provide some useful anti-commutator properties of $\mathcal {C}_\lambda$ and the Dirac matrices. These facts are also important ingredients to prove the self-adjointness of Dirac operators on domains later.

#### Proposition 3.11

Let $\lambda \in \mathbb {C} {\setminus } ( (-\infty , - m] \cup [m, \infty ) )$ and let $\mathcal {C}_\lambda$ be the operator in (3.9). Then, the following statements hold:

(i) The mapping $\mathcal {A}_\lambda := \mathcal {C}_\lambda ^2 - \frac{1}{4} I_4$ can be extended to a bounded operator
  $$\begin{aligned} \widetilde{\mathcal {A}}_\lambda : H^{-1/2}({\partial \Omega }; \mathbb {C}^4) \rightarrow H^{1/2}({\partial \Omega }; \mathbb {C}^4). \end{aligned}$$
(ii) The anti-commutator $\mathcal {B}_\lambda := \mathcal {C}_\lambda \beta + \beta \mathcal {C}_\lambda$ can be extended to a bounded operator
  $$\begin{aligned} \widetilde{\mathcal {B}}_\lambda : H^{-1/2}({\partial \Omega }; \mathbb {C}^4) \rightarrow H^{1/2}({\partial \Omega }; \mathbb {C}^4). \end{aligned}$$

In particular, the restrictions

$$\begin{aligned} \mathcal {A}_{\lambda ,1/2}=\mathcal {C}_{\lambda ,1/2}^2 - \frac{1}{4} I_4\quad \text {and}\quad \mathcal {B}_{\lambda ,1/2}=\mathcal {C}_{\lambda ,1/2} \beta + \beta \mathcal {C}_{\lambda ,1/2} \end{aligned}$$

onto $H^{1/2}(\partial \Omega ; \mathbb {C}^4)$ are both compact operators in $H^{1/2}(\partial \Omega ; \mathbb {C}^4)$.

#### Proof

The proof of item (i) can be found in [15, Proposition 4.4] and [56, Proposition 2.8]. It remains to show statement (ii). Using the anti-commutation relation (1.8), we see that $\mathcal {B}_\lambda$ is an integral operator with kernel

$$\begin{aligned} b_\lambda (x-y) = 2 \left( \lambda \beta + m I_4 \right) \frac{e^{i\sqrt{\lambda ^2 - m^2}|x-y|}}{4 \pi |x-y|} \end{aligned}$$

and thus $\mathcal {B}_\lambda = 2 \big ( \lambda \beta + m I_4 \big ) \mathrm {SL}_{\lambda ^2 - m^2}$, where $\mathrm {SL}_\mu$ denotes the single-layer boundary integral operator for $-\Delta -\mu$, see, e.g., [53, equation (9.15)]. It is well known that $\mathrm {SL}_{\lambda ^2 - m^2}$ gives rise to a bounded operator from $H^{-1/2}({\partial \Omega }; \mathbb {C})$ to $H^{1/2}({\partial \Omega }; \mathbb {C})$, see, for instance, [53, Theorem 6.11]. This yields assertion (ii).

$$\square $$

Next, we state a result on the invertibility of $\pm \frac{1}{2} \beta + \mathcal {C}_{\lambda , s}$ which will be used in the construction of the $\gamma$-field and the Weyl function associated with a quasi boundary triple for a Dirac operator. To formulate the result, we recall the definitions of $T_\text {MIT}$ and $T_{-\text {MIT}}$ from (3.4) and (3.5), respectively, and denote by $T_\text {MIT}^{\Omega ^c}$ and $T_{-\mathrm{MIT}}^{\Omega ^c}$ the Dirac operator in $L^2(\mathbb R^3{\setminus } \overline{\Omega }; \mathbb {C}^4)$ with the same boundary conditions on $\partial \Omega$ as $T_\text {MIT}$ and $T_{-\text {MIT}}$, respectively.

#### Proposition 3.12

Let $s \in [-\frac{1}{2}, \frac{1}{2} ]$ and let $\mathcal {C}_{\lambda ,s}$ be the operator in Proposition 3.8. Then, the following statements hold:

(i) For all $\lambda \not \in (-\infty , -m] \cup [m, \infty )$, the operator $\frac{1}{2} \beta + \mathcal {C}_{\lambda , s}$ admits a bounded and everywhere defined inverse in $H^s(\partial \Omega ; \mathbb {C}^4)$.
(ii) For all $\lambda \notin (-\infty , -m] \cup [m, \infty ) \cup \sigma _{\mathrm {p}}(T_{-{\mathrm {MIT}}}) \cup \sigma _{\mathrm {p}}(T_{-{\mathrm {MIT}}}^{\Omega ^c})$, the operator $-\frac{1}{2} \beta + {\mathcal {C}}_{\lambda , s}$ admits a bounded and everywhere defined inverse in $H^s(\partial \Omega ; \mathbb {C}^4)$.

#### Proof

(i) We prove the claim for $s=\frac{1}{2}$ and $\lambda \in \mathbb {C} {\setminus } ( (-\infty , -m] \cup [m, \infty ))$. For this it suffices to verify that $\frac{1}{2} \beta + \mathcal {C}_{\lambda , 1/2}$ is bijective in $H^{1/2}(\partial \Omega ; \mathbb {C}^4)$. Then, the claim for $s=-\frac{1}{2}$ follows by duality, as $\mathcal {C}_{\overline{\lambda }, -1/2} = \mathcal {C}_{\lambda , 1/2}'$, and for $s \in ( -\frac{1}{2}, \frac{1}{2})$ by interpolation.

Let us verify that $\frac{1}{2} \beta + \mathcal {C}_{\lambda , 1/2}$ is injective. Indeed, assume that we have $\big (\frac{1}{2} \beta + \mathcal {C}_{\lambda , 1/2}\big ) \varphi = 0$ for some $\varphi \in H^{1/2}(\partial \Omega ; \mathbb {C}^4)$, $\varphi \not =0$. We claim that then $\lambda$ is an eigenvalue of the MIT bag operator in $\Omega$ or in $\Omega ^c$, which is not possible by Proposition 3.3 (i). Define the functions

3.19 $$\begin{aligned} f_\Omega (x) := \Phi _{\lambda , 1/2} \varphi (x) = \int _{\partial \Omega } G_\lambda (x-y) \varphi (y) \hbox {d} \sigma (y), \qquad x \in \Omega , \end{aligned}$$

and

3.20 $$\begin{aligned} f_{\Omega ^c}(x) := \int _{\partial \Omega } G_\lambda (x-y) \varphi (y) \hbox {d} \sigma (y), \qquad x \in \mathbb R^3{\setminus }\overline{\Omega }. \end{aligned}$$

We claim first that $f_\Omega \in \mathrm {dom}\,T_\mathrm{MIT}$ and $f_{\Omega ^c} \in \mathrm {dom}\,T_\mathrm{MIT}^{\Omega ^c}$. This is shown for $f_\Omega$, the proof for $f_{\Omega ^c}$ is similar using that the unit normal vector field for $\Omega ^c$ is $-\nu$. First, since $\varphi \in H^{1/2}(\partial \Omega ; \mathbb {C}^4)$, one has $f_\Omega \in H^1(\Omega ; \mathbb {C}^4)$ by Proposition 3.6 (i). Moreover, according to Lemma 3.9 the trace of $f_\Omega$ is

$$\begin{aligned} f_\Omega |_{\partial \Omega } = -\frac{i}{2} (\alpha \cdot \nu ) \varphi + \mathcal {C}_{\lambda , 1/2} \varphi , \end{aligned}$$

and, because of $(\alpha \cdot \nu )^2 = I_4$ (which follows from (1.8)), it satisfies

$$\begin{aligned} \big (I_4 + i \beta (\alpha \cdot \nu )\big ) f_\Omega |_{\partial \Omega }&= \big (I_4 + i \beta (\alpha \cdot \nu )\big ) \left( -\frac{i}{2} (\alpha \cdot \nu ) \varphi + \mathcal {C}_{\lambda , 1/2} \varphi \right) \\&= \left( \frac{1}{2} \beta + \mathcal {C}_{\lambda , 1/2} \right) \varphi + i \beta (\alpha \cdot \nu ) \left( \frac{1}{2} \beta + \mathcal {C}_{\lambda , 1/2} \right) \varphi \\&= 0, \end{aligned}$$

i.e., $f_\Omega \in \mathrm {dom}\,T_\mathrm{MIT}$. Since the unit normal vector field of $\Omega ^c$ is $-\nu$, one concludes from Proposition 3.6 (iv) that the trace of $f_{\Omega ^c}$ is

$$\begin{aligned} f_{\Omega ^c}|_{\partial \Omega } = \frac{i}{2} (\alpha \cdot \nu ) \varphi + \mathcal {C}_{\lambda , 1/2} \varphi , \end{aligned}$$

and hence,

$$\begin{aligned} f_\Omega |_{\partial \Omega } - f_{\Omega ^c}|_{\partial \Omega } = -i (\alpha \cdot \nu ) \varphi \ne 0, \end{aligned}$$

which shows that at least one of the functions $f_\Omega$ and $f_{\Omega ^c}$ is not trivial. From Proposition 3.6 (ii), we get $(T_\mathrm{MIT} - \lambda ) f_\Omega = 0$ and in the same way one concludes $(T_\mathrm{MIT}^{\Omega ^c} - \lambda ) f_{\Omega ^c} = 0$, i.e. $\lambda \in \mathbb {C} {\setminus } ( (-\infty , -m] \cup [m, \infty ) )$ is an eigenvalue of $T_\mathrm{MIT}$ or $T_\mathrm{MIT}^{\Omega ^c}$, which leads to a contradiction. Therefore, $\frac{1}{2} \beta + \mathcal {C}_{\lambda , 1/2}$ is injective.

It remains to prove that $\frac{1}{2} \beta + \mathcal {C}_{\lambda , 1/2}$ is surjective. For that, we note first that

$$\begin{aligned} {{\,\mathrm{ran}\,}}\big ( \tfrac{1}{2} \beta + \mathcal {C}_{\lambda , 1/2} \big ) \supset {{\,\mathrm{ran}\,}}\big ( \tfrac{1}{2} \beta + \mathcal {C}_{\lambda , 1/2} \big )^2, \end{aligned}$$

and from Proposition 3.11, we obtain

$$\begin{aligned} \left( \frac{1}{2} \beta + \mathcal {C}_{\lambda , 1/2} \right) ^2&= \frac{1}{2} I_4 + \frac{1}{2} \big ( \mathcal {C}_{\lambda , 1/2} \beta + \beta \mathcal {C}_{\lambda , 1/2} \big ) + (\mathcal {C}_{\lambda , 1/2})^2-\frac{1}{4} I_4\\&=\frac{1}{2} I_4 + \frac{1}{2}\mathcal {B}_{\lambda ,1/2}+ \mathcal {A}_{\lambda ,1/2}, \end{aligned}$$

where $\frac{1}{2}\mathcal {B}_{\lambda ,1/2}+ \mathcal {A}_{\lambda ,1/2}$ is a compact operator in $H^{1/2}(\partial \Omega ; \mathbb {C}^4)$. Therefore, as the operator $\big ( \tfrac{1}{2} \beta + \mathcal {C}_{\lambda , 1/2} \big )^2$ is injective (since $\tfrac{1}{2} \beta + \mathcal {C}_{\lambda , 1/2}$ is injective), Fredholm’s alternative shows that $\big ( \tfrac{1}{2} \beta + \mathcal {C}_{\lambda , 1/2} \big )^2$, and hence also $\tfrac{1}{2} \beta + \mathcal {C}_{\lambda , 1/2}$, is surjective. This finishes the proof of item (i).

The proof of item (ii) follows the lines of assertion (i); one just has to note that for $\varphi \in \ker \big ( -\frac{1}{2} \beta + \mathcal {C}_{\lambda , 1/2} \big )$, the functions $f_\Omega$ and $f_{\Omega ^c}$ defined by (3.19) and (3.20) belong to $\mathrm {dom}\,T_{-\mathrm{MIT}}$ and $\mathrm {dom}\,T_{-\mathrm{MIT}}^{\Omega ^c}$, respectively. $\square$

We now discuss that the derivatives of the integral operators $\Phi _\lambda$ and $\mathcal {C}_\lambda$ belong to certain (weak) Schatten–von Neumann ideals. For that, we use the following result on operators with range in the Sobolev space $H^s({\partial \Omega }; \mathbb {C})$. Its proof follows word-by-word the one of [11, Proposition 2.4]; hence, we omit it here.

#### Proposition 3.13

Let $l \in \mathbb {N}$, let ${\partial \Omega } \subset \mathbb {R}^3$ be the boundary of a compact $C^l$-smooth domain, and let $k \in \{ 1, \dots , 2 l - 1\}$. Let $\mathcal {H}$ be a separable Hilbert space and assume that $A: \mathcal {H} \rightarrow L^2({\partial \Omega }; \mathbb {C}^4)$ is continuous with ${{\,\mathrm{ran}\,}}A \subset H^{k/2}({\partial \Omega }; \mathbb {C}^4)$. Then, $A \in \mathfrak {S}_{4/k, \infty }\big (\mathcal {H}, L^2({\partial \Omega }; \mathbb {C}^4) \big )$.

With the help of Proposition 3.13, one can show in exactly the same way as in [10, Lemma 4.5] the following result; note that the operators $\gamma (\lambda )$ and $M(\lambda )$ in [10] coincide with $\Phi _\lambda$ and $\mathcal {C}_\lambda$, respectively.

#### Lemma 3.14

Let $l \in \mathbb {N}$ with $l \ge 2$ and let ${\partial \Omega } \subset \mathbb {R}^3$ be the boundary of a compact $C^l$-smooth domain. Moreover, let $\lambda \in \mathbb {C} {\setminus } ( (-\infty , -m] \cup [m, \infty ))$ and let $\Phi _\lambda$ and $\mathcal {C}_\lambda$ be the operators in (3.8) and (3.9), respectively. Then, the following statements hold:

(i) The operator-valued functions $\lambda \mapsto \Phi _\lambda$ and $\lambda \mapsto \Phi _{\overline{\lambda }}^*$ are holomorphic and, for any $k \in \{ 0, 1, \dots , l-1 \}$,
  $$\begin{aligned} \frac{\hbox {d}^k}{\hbox {d} \lambda ^k} \Phi _\lambda \in \mathfrak {S}_{4/(2k+1), \infty }\big ( L^2(\partial \Omega ; \mathbb {C}^4), L^2(\Omega ; \mathbb {C}^4) \big ) \end{aligned}$$
  and
  $$\begin{aligned} \frac{\hbox {d}^k}{\hbox {d} \lambda ^k} \Phi _{\overline{\lambda }}^* \in \mathfrak {S}_{4/(2k+1), \infty }\big ( L^2(\Omega ; \mathbb {C}^4), L^2(\partial \Omega ; \mathbb {C}^4) \big ). \end{aligned}$$
  In particular, $\Phi _\lambda$ and $\Phi _\lambda ^*$ are compact.
(ii) The mapping $\lambda \mapsto \mathcal {C}_\lambda$ is holomorphic, $\frac{\hbox {d}}{\hbox {d} \lambda } \mathcal {C}_\lambda = \Phi _{\overline{\lambda }}^* \Phi _\lambda$ and, for any integer $k \in \{ 1, \dots , l\}$,
  $$\begin{aligned} \frac{\hbox {d}^k}{\hbox {d} \lambda ^k} \mathcal {C}_\lambda \in \mathfrak {S}_{2/k, \infty }\big ( L^2(\partial \Omega ; \mathbb {C}^4) \big ). \end{aligned}$$

## A Quasi Boundary Triple for Dirac Operators on Domains

In this section, we introduce a quasi boundary triple which is useful to define self-adjoint Dirac operators on domains via suitable boundary conditions on $\partial \Omega$. Throughout this section, let $\Omega$ be a bounded or unbounded domain in $\mathbb {R}^3$ with a compact $C^2$-smooth boundary. As before, we denote the normal vector field at $\partial \Omega$ pointing outwards of $\Omega$ by $\nu$. In the following, the operators

4.1 $$\begin{aligned} P_\pm : L^2(\partial \Omega ; \mathbb {C}^4)\rightarrow L^2(\partial \Omega ; \mathbb {C}^4),\quad \varphi \mapsto \frac{1}{2} \big ( I_4 \pm i \beta (\alpha \cdot \nu )\big )\varphi , \end{aligned}$$

will play an important role. The relation $P_- = I - P_+$ is clear. Furthermore, using the anti-commutation relation (1.8) it is easy to see that $P_\pm ^2=P_\pm$ and $P_+ P_- = P_- P_+ = 0$, that is, $P_\pm$ are orthogonal projections in $L^2(\partial \Omega ; \mathbb {C}^4)$. We also note that (1.8) implies

4.2 $$\begin{aligned} P_+\beta =\beta P_-. \end{aligned}$$

We shall make use of the spaces

4.3 $$\begin{aligned} \mathcal {G}_\Omega ^s := P_+\bigl (H^s(\partial \Omega ; \mathbb {C}^4)\bigr ),\qquad s \in \bigl [0, \tfrac{1}{2} \bigr ]. \end{aligned}$$

For convenience of notation, we simply write $\mathcal {G}_\Omega := \mathcal {G}_\Omega ^0$. As $P_+$ is an orthogonal projection in $L^2(\partial \Omega ; \mathbb {C}^4)$ the space $\mathcal {G}_\Omega$ is a Hilbert space. Moreover, since $\partial \Omega$ is $C^2$-smooth, the normal vector field $\nu$ is Lipschitz continuous and hence it follows from Lemma A.2 that $\mathcal {G}_\Omega ^s \subset H^s(\partial \Omega ; \mathbb {C}^4)$ for $s\in [0, \frac{1}{2}]$. Furthermore, it is not difficult to check that $\mathcal {G}_\Omega ^s$ is a closed subspace of $H^s(\partial \Omega ; \mathbb {C}^4)$ for $s\in [0, \frac{1}{2}]$. In the sequel the spaces $\mathcal {G}_\Omega ^s$ are always equipped with the norm of $H^s(\partial \Omega ; \mathbb {C}^4)$.

Next, we define the operator *T* in $L^2(\Omega ; \mathbb {C}^4)$ by

4.4 $$\begin{aligned} T f = (-i \alpha \cdot \nabla + m \beta ) f, \qquad \mathrm {dom}\,T = H^1(\Omega ; \mathbb {C}^4), \end{aligned}$$

and the mappings $\Gamma _0, \Gamma _1: \mathrm {dom}\,T \rightarrow \mathcal {G}_\Omega$ acting as

4.5 $$\begin{aligned} \Gamma _0 f = P_+ f|_{\partial \Omega } \quad \text {and} \quad \Gamma _1 f = P_+ \beta f|_{\partial \Omega }, \quad f \in \mathrm {dom}\,T. \end{aligned}$$

In the following theorem, we show that $\{ \mathcal {G}_\Omega , \Gamma _0, \Gamma _1 \}$ is a quasi boundary triple and that $\overline{T}$ coincides with the maximal Dirac operator $T_{\mathrm{max}}$ from (3.1). Moreover, it turns out that the reference operator $T \upharpoonright \ker \Gamma _0$ is the MIT bag operator studied in Sect. 3.2.

### Theorem 4.1

Let $T_{\mathrm{min}}$ be the closed symmetric operator from (3.2), let $\mathcal {G}_\Omega$ be given by (4.3), and let $T,\, \Gamma _0$, and $\Gamma _1$ be as in (4.4) and (4.5), respectively. Then, $\overline{T} = T_{\mathrm{min}}^*= T_{\mathrm{max}}$ and $\{ \mathcal {G}_\Omega , \Gamma _0, \Gamma _1 \}$ is a quasi boundary triple for $T \subset T_{\mathrm{max}}$ such that

4.6 $$\begin{aligned} T_{\mathrm{MIT}}=T \upharpoonright \ker \Gamma _0\quad \text {and}\quad T_{-\mathrm{MIT}}=T \upharpoonright \ker \Gamma _1. \end{aligned}$$

Moreover,

4.7 $$\begin{aligned} {{\,\mathrm{ran}\,}}(\Gamma _0 \upharpoonright \ker \Gamma _1) = {{\,\mathrm{ran}\,}}(\Gamma _1 \upharpoonright \ker \Gamma _0) = \mathcal {G}_\Omega ^{1/2}, \end{aligned}$$

and hence, in particular, ${{\,\mathrm{ran}\,}}(\Gamma _0, \Gamma _1)^\top = \mathcal {G}_\Omega ^{1/2} \times \mathcal {G}_\Omega ^{1/2}$.

### Proof

First, we have $T_{\mathrm{min}}^* = T_{\mathrm{max}}$ by Lemma 3.1. Furthermore, Lemma 3.1 also implies that the closure of *T* coincides with $T_{\mathrm{max}}$ , as $C^\infty (\overline{\Omega };\mathbb {C}^4) \subset \mathrm {dom}\,T$ is dense in $\mathrm {dom}\,T_{\mathrm{max}}$ equipped with the graph norm.

Now we verify that the abstract Green’s identity is valid. For this, consider $f, g \in \mathrm {dom}\,T = H^1(\Omega ; \mathbb {C}^4)$. Then, (3.3) and the self-adjointness of $\alpha \cdot \nu$ yield

$$\begin{aligned} ( T f, g )_{\Omega } - (f, T g)_\Omega&= \big ( (-i \alpha \cdot \nabla + m \beta ) f, g \big )_{\Omega } - \big ( f, (-i \alpha \cdot \nabla + m \beta ) g \big )_{\Omega } \\&= \big ( - i (\alpha \cdot \nu ) f|_{\partial \Omega }, g|_{\partial \Omega }\big )_{\partial \Omega } \\&= \frac{1}{2} \big ( -i (\alpha \cdot \nu ) f|_{\partial \Omega }, g|_{\partial \Omega }\big )_{\partial \Omega } - \frac{1}{2} \big ( f|_{\partial \Omega }, -i (\alpha \cdot \nu ) g|_{\partial \Omega }\big )_{\partial \Omega }. \end{aligned}$$

Using that $\beta$ is unitary and self-adjoint, we see that the last expression is equal to

$$\begin{aligned} \frac{1}{2} \big (&-i \beta (\alpha \cdot \nu ) f|_{\partial \Omega }, \beta g|_{\partial \Omega }\big )_{\partial \Omega } - \frac{1}{2} \big ( \beta f|_{\partial \Omega }, -i \beta (\alpha \cdot \nu ) g|_{\partial \Omega }\big )_{\partial \Omega } \\&= \frac{1}{2} \big ( \beta f|_{\partial \Omega }, g|_{\partial \Omega } + i \beta (\alpha \cdot \nu ) g|_{\partial \Omega }\big )_{\partial \Omega } - \frac{1}{2} \big ( f|_{\partial \Omega } + i \beta (\alpha \cdot \nu ) f|_{\partial \Omega }, \beta g|_{\partial \Omega }\big )_{\partial \Omega } \\&= ( \beta f|_{\partial \Omega }, P_+ g|_{\partial \Omega } )_{\partial \Omega } - ( P_+ f|_{\partial \Omega }, \beta g|_{\partial \Omega })_{\partial \Omega }. \end{aligned}$$

Since $P_+$ is an orthogonal projection in $L^2(\partial \Omega ; \mathbb {C}^4)$, we conclude

$$\begin{aligned} (T f, g)_\Omega - (f, T g)_\Omega&= (P_+ \beta f|_{\partial \Omega }, P_+ g|_{\partial \Omega } )_{\partial \Omega } - (P_+ f|_{\partial \Omega }, P_+ \beta g|_{\partial \Omega })_{\partial \Omega } \\&= ( \Gamma _1 f, \Gamma _0 g )_{\partial \Omega } - ( \Gamma _0 f, \Gamma _1 g)_{\partial \Omega }, \end{aligned}$$

which is the abstract Green’s identity.

Next, we check the range property (4.7). Clearly, by the definition of $\Gamma _0$ and $\Gamma _1$, $\mathrm {dom}\,\Gamma _0 = \mathrm {dom}\,\Gamma _1 = H^1(\Omega ; \mathbb {C}^4)$, and by standard properties of the trace map and Lemma A.2 one has ${{\,\mathrm{ran}\,}}\Gamma _0\subset \mathcal {G}_\Omega ^{1/2}$ and ${{\,\mathrm{ran}\,}}\Gamma _1 \subset \mathcal {G}_\Omega ^{1/2}$, and hence also

4.8 $$\begin{aligned} {{\,\mathrm{ran}\,}}(\Gamma _0 \upharpoonright \ker \Gamma _1)\subset \mathcal {G}_\Omega ^{1/2}\quad \text {and}\quad {{\,\mathrm{ran}\,}}(\Gamma _1 \upharpoonright \ker \Gamma _0) \subset \mathcal {G}_\Omega ^{1/2}. \end{aligned}$$

To prove $\mathcal {G}_\Omega ^{1/2} \subset {{\,\mathrm{ran}\,}}(\Gamma _0 \upharpoonright \ker \Gamma _1)$, let $\varphi \in \mathcal {G}_\Omega ^{1/2}$ and choose $f \in H^1(\Omega ; \mathbb {C}^4)$ such that $f|_{\partial \Omega } = \varphi$. Since $\varphi \in \mathcal {G}_\Omega$, we have $P_+ \varphi =\varphi$ and $P_-\varphi =0$. Hence,

$$\begin{aligned} \Gamma _0 f = P_+ f|_{\partial \Omega } = P_+ \varphi = \varphi , \end{aligned}$$

and using (4.2), we obtain

$$\begin{aligned} \Gamma _1 f = P_+ \beta f|_{\partial \Omega } = P_+ \beta \varphi = \beta P_- \varphi = 0, \end{aligned}$$

that is, $\varphi \in {{\,\mathrm{ran}\,}}(\Gamma _0 \upharpoonright \ker \Gamma _1)$. To prove $\mathcal {G}_\Omega ^{1/2} \subset {{\,\mathrm{ran}\,}}(\Gamma _1 \upharpoonright \ker \Gamma _0)$, let $\psi \in \mathcal {G}_\Omega ^{1/2}$ and choose $g \in H^1(\Omega ; \mathbb {C}^4)$ such that $g|_{\partial \Omega } = \beta \psi$. Since $\psi \in \mathcal {G}_\Omega$, we have $P_+ \psi =\psi$ and $P_- \psi =0$. Hence, using (4.2) we obtain

$$\begin{aligned} \Gamma _0 g = P_+ g|_{\partial \Omega } = P_+ \beta \psi = \beta P_- \psi = 0 \end{aligned}$$

and

$$\begin{aligned} \Gamma _1 g = P_+ \beta g|_{\partial \Omega } = P_+ \beta ^2 \psi = \psi , \end{aligned}$$

that is, $\psi \in {{\,\mathrm{ran}\,}}(\Gamma _1 \upharpoonright \ker \Gamma _0)$. Together with (4.8), we conclude (4.7).

Finally, observe that

$$\begin{aligned} \ker \Gamma _0&= \{ f \in H^1(\Omega ; \mathbb {C}^4): f|_{\partial \Omega } = -i \beta (\alpha \cdot \nu ) f|_{\partial \Omega } \} = \mathrm {dom}\,T_{\mathrm {MIT}},\\ \ker \Gamma _1&= \{ f \in H^1(\Omega ; \mathbb {C}^4): f|_{\partial \Omega } = i \beta (\alpha \cdot \nu ) f|_{\partial \Omega } \} = \mathrm {dom}\,T_{-\mathrm {MIT}}. \end{aligned}$$

Therefore, $T \upharpoonright \ker \Gamma _0$ coincides with the MIT bag Dirac operator $T_{\mathrm{MIT}}$ and $T \upharpoonright \ker \Gamma _1$ coincides with $T_{-\mathrm{MIT}}$, which shows (4.6). Note that both operators are self-adjoint; cf. Propositions 3.3 and 3.4. Thus, $\{ \mathcal {G}_\Omega , \Gamma _0, \Gamma _1 \}$ is a quasi boundary triple for $T \subset T_{\mathrm{max}}$. $\square$

Now we compute the $\gamma$-field and the Weyl function associated with the quasi boundary triple in Theorem 4.1. It turns out that these operators are closely related to the integral operators $\Phi _\lambda$ and $\mathcal {C}_\lambda$ defined in Sect. 3.3. For the next proposition, recall that $\frac{1}{2} \beta + \mathcal {C}_{\lambda , 1/2}$ admits a bounded and everywhere defined inverse in $H^{1/2}({\partial \Omega }; \mathbb {C}^4)$ for $\lambda \in \mathbb {C} {\setminus } ( (-\infty , -m] \cup [m, \infty ))$, see Proposition 3.12.

### Proposition 4.2

Let $\{ \mathcal {G}_\Omega , \Gamma _0, \Gamma _1 \}$ be the quasi boundary triple from Theorem 4.1, let $\lambda \in \mathbb {C} {\setminus } ( (-\infty , -m] \cup [m, \infty ) ) \subset \rho (T_{\mathrm{MIT}})$, and let $\Phi _{\lambda , 1/2}$ and $\mathcal {C}_{\lambda , 1/2}$ be the operators in Propositions 3.6 and 3.8, respectively. Then, the following statements hold:

(i) The value of the $\gamma$-field corresponding to $\{ \mathcal {G}_\Omega , \Gamma _0, \Gamma _1 \}$ is given by
  4.9 $$\begin{aligned} \gamma (\lambda ) = \Phi _{\lambda , 1/2} \left( \frac{1}{2} \beta + \mathcal {C}_{\lambda , 1/2} \right) ^{-1},\quad \mathrm {dom}\,\gamma (\lambda ) = \mathcal {G}_\Omega ^{1/2}. \end{aligned}$$
  Each $\gamma (\lambda )$ is a densely defined bounded operator from $\mathcal {G}_\Omega$ to $L^2(\Omega ; \mathbb {C}^4)$, and a bounded and everywhere defined operator from $\mathcal {G}_\Omega ^{1/2}$ to $H^1(\Omega ; \mathbb {C}^4)$.
(ii) The adjoint $\gamma (\lambda )^*$ of the $\gamma$-field corresponding to $\{ \mathcal {G}_\Omega , \Gamma _0, \Gamma _1 \}$ is given by
  $$\begin{aligned} \gamma (\lambda )^* = P_+ \left( \frac{1}{2} \beta + \mathcal {C}_{\overline{\lambda }, 1/2} \right) ^{-1} \Phi _{\lambda }^*. \end{aligned}$$
  Each $\gamma (\lambda )^*$ is bounded from $L^2(\Omega ; \mathbb {C}^4)$ to $\mathcal {G}_\Omega ^{1/2}$, and, in particular, compact from $L^2(\Omega ; \mathbb {C}^4)$ to $\mathcal {G}_\Omega$.
(iii) The value of the Weyl function corresponding to $\{ \mathcal {G}_\Omega , \Gamma _0, \Gamma _1 \}$ is given by
  4.10 $$\begin{aligned} M(\lambda ) = - P_+ \left( \frac{1}{2} \beta + \mathcal {C}_{\lambda , 1/2} \right) ^{-1} P_+,\quad \mathrm {dom}\,M(\lambda ) = \mathcal {G}_\Omega ^{1/2}. \end{aligned}$$
  Each $M(\lambda )$ is a densely defined and bounded operator in $\mathcal {G}_\Omega$, and a bounded and everywhere defined operator in $\mathcal {G}_\Omega ^{1/2}$.

### Proof

In the following, let $\lambda \in \mathbb {C} {\setminus } ( (-\infty , -m] \cup [m, \infty ))$ be fixed. From (4.7) and the definition of the $\gamma$-field and Weyl function, it follows that

$$\begin{aligned} \mathrm {dom}\,\gamma (\lambda ) = \mathrm {dom}\,M(\lambda ) = {{\,\mathrm{ran}\,}}\Gamma _0 = \mathcal {G}_\Omega ^{1/2}. \end{aligned}$$

For the proof of item (i), consider $\varphi \in {{\,\mathrm{ran}\,}}\Gamma _0 = \mathcal {G}_\Omega ^{1/2}$ and recall that $\gamma (\lambda ) \varphi$ is the unique solution of the boundary value problem

4.11 $$\begin{aligned} (T - \lambda ) f = 0 \quad \text {and} \quad \Gamma _0 f = \varphi . \end{aligned}$$

We set

$$\begin{aligned} f_\lambda = \Phi _{\lambda , 1/2} \left( \frac{1}{2} \beta + \mathcal {C}_{\lambda , 1/2} \right) ^{-1} \varphi . \end{aligned}$$

Then, due to the mapping properties of $\Phi _{\lambda , 1/2}$ and $\big ( \frac{1}{2} \beta + \mathcal {C}_{\lambda , 1/2} \big )^{-1}$, see Propositions 3.6 and 3.12, we have $f_\lambda \in H^1(\Omega ; \mathbb {C}^4) = \mathrm {dom}\,T$. For (4.9), it suffices to check that $f_\lambda$ solves the boundary value problem (4.11). In fact, by Proposition 3.6 (ii) we have $(T - \lambda ) f_\lambda = 0$ and using Lemma 3.9 we get

$$\begin{aligned} \Gamma _0 f_\lambda&= P_+ f_\lambda |_{\partial \Omega } = P_+ \left( -\frac{i}{2} (\alpha \cdot \nu ) + \mathcal {C}_{\lambda ,1/2} \right) \left( \frac{1}{2} \beta + \mathcal {C}_{\lambda , 1/2} \right) ^{-1} \varphi \\&= P_+ \left( -\frac{i}{2} (\alpha \cdot \nu ) - \frac{1}{2} \beta + \frac{1}{2} \beta + \mathcal {C}_{\lambda ,1/2} \right) \left( \frac{1}{2} \beta + \mathcal {C}_{\lambda , 1/2} \right) ^{-1} \varphi \\&= P_+ \left( -\frac{i}{2} (\alpha \cdot \nu ) \beta - \frac{1}{2} I_4 \right) \beta \left( \frac{1}{2} \beta + \mathcal {C}_{\lambda , 1/2} \right) ^{-1} \varphi + P_+ \varphi . \end{aligned}$$

Using that $\varphi \in \mathcal {G}_\Omega$, (1.8), and $P_+P_-=0$, we obtain then

$$\begin{aligned} \Gamma _0 f_\lambda&= -\, P_+ \frac{1}{2}\bigl ( I_4 - i \beta (\alpha \cdot \nu ) \bigr ) \beta \left( \frac{1}{2} \beta + \mathcal {C}_{\lambda , 1/2} \right) ^{-1} \varphi +\varphi \\&= -\, P_+ P_- \beta \left( \frac{1}{2} \beta + \mathcal {C}_{\lambda , 1/2} \right) ^{-1} \varphi + \varphi = \varphi . \end{aligned}$$

Hence, $f_\lambda$ is the unique solution of the boundary value problem (4.11). This implies $\gamma (\lambda ) \varphi = f_\lambda$ and leads to representation (4.9).

It remains to check the mapping properties of $\gamma (\lambda )$ in (i). From the definition of the $\gamma$-field, it is clear that $\gamma (\lambda )$ is a densely defined bounded operator from $\mathcal {G}_\Omega$ to $L^2(\Omega ; \mathbb {C}^4)$. Moreover, from Propositions 3.6 (i) and 3.12 (i) it also follows that $\gamma (\lambda )$ is a bounded and everywhere defined operator from $\mathcal {G}_\Omega ^{1/2}$ to $H^1(\Omega ; \mathbb {C}^4)$.

Next we prove item (ii). Let $f \in L^2(\Omega ; \mathbb {C}^4)$ and $\varphi \in \mathcal {G}_\Omega ^{1/2} = \mathrm {dom}\,\gamma (\lambda )$ be fixed. Then, using (3.10) we find that

$$\begin{aligned} \big (\varphi , \gamma (\lambda )^* f \big )_{\partial \Omega }&= \big (\gamma (\lambda ) \varphi , f \big )_{\Omega } = \left( \Phi _{\lambda , 1/2} \left( \frac{1}{2} \beta + \mathcal {C}_{\lambda , 1/2} \right) ^{-1} \varphi , f \right) _{\Omega } \\&= \left( \left( \frac{1}{2} \beta + \mathcal {C}_{\lambda } \right) ^{-1} \varphi , \Phi _\lambda ^* f \right) _{\partial \Omega } = \left( \varphi , \left( \frac{1}{2} \beta + \mathcal {C}_{\overline{\lambda }} \right) ^{-1} \Phi _\lambda ^* f \right) _{\partial \Omega }. \end{aligned}$$

Since this holds for all $\varphi \in \mathcal {G}_\Omega ^{1/2}$ and $\Phi _\lambda ^*: L^2(\Omega ; \mathbb {C}^4) \rightarrow H^{1/2}(\partial \Omega ; \mathbb {C}^4)$ by (3.11), we find the claimed representation for $\gamma (\lambda )^*$. Moreover, since $(\frac{1}{2} \beta + \mathcal {C}_{\overline{\lambda }, 1/2})^{-1}$ is bounded in $H^{1/2}(\partial \Omega ; \mathbb {C}^4)$ by Proposition 3.12, we get that $\gamma (\lambda )^*$ is bounded from $L^2(\Omega ; \mathbb {C}^4)$ to $\mathcal {G}_\Omega ^{1/2}$ and compact from $L^2(\Omega ; \mathbb {C}^4)$ to $\mathcal {G}_\Omega$, since $\mathcal {G}_\Omega ^{1/2} = P_+ (H^{1/2}(\partial \Omega ; \mathbb {C}^4))$ is compactly embedded in $\mathcal {G}_\Omega = P_+ (L^2(\partial \Omega ; \mathbb {C}^4))$.

Finally, we show assertion (iii). For this, we use (4.9), Lemma 3.9, and compute for $\varphi \in \mathcal {G}_\Omega ^{1/2}$

$$\begin{aligned} M(\lambda ) \varphi&= \Gamma _1 \gamma (\lambda ) \varphi = P_+ \beta \left( \Phi _{\lambda , 1/2} \left( \frac{1}{2} \beta + \mathcal {C}_{\lambda , 1/2} \right) ^{-1} \varphi \right) \Bigg |_{\partial \Omega } \\&= P_+ \beta \left( -\frac{i}{2} (\alpha \cdot \nu ) - \frac{1}{2} \beta + \frac{1}{2} \beta + \mathcal {C}_{\lambda ,1/2} \right) \left( \frac{1}{2} \beta + \mathcal {C}_{\lambda , 1/2} \right) ^{-1} \varphi \\&= P_+ \left( -\frac{i}{2} \beta (\alpha \cdot \nu ) - \frac{1}{2} I_4 \right) \left( \frac{1}{2} \beta + \mathcal {C}_{\lambda , 1/2} \right) ^{-1} \varphi + P_+ \beta \varphi \\&= -\, P_+^2 \left( \frac{1}{2} \beta + \mathcal {C}_{\lambda , 1/2} \right) ^{-1} \varphi + P_+ \beta \varphi . \end{aligned}$$

Since $P_+^2 = P_+$ and $P_+ \beta \varphi =\beta P_-\varphi =0$ (see (4.2)) for $\varphi \in \mathcal {G}_\Omega ^{1/2}$, the representation (4.10) for the Weyl function follows.

It is a consequence of mapping properties of $( \frac{1}{2} \beta + \mathcal {C}_{\lambda , 1/2})^{-1}$ from Proposition 3.12 (i) that each $M(\lambda )$ is a densely defined and bounded operator in $\mathcal {G}_\Omega$, and a bounded and everywhere defined operator in $\mathcal {G}_\Omega ^{1/2}$. $\square$

In the next proposition, we derive a useful formula for the inverse of $M(\lambda )$. Let $T_{-\text {MIT}}$ be given by (3.5) and let $T_{-\text {MIT}}^{\Omega ^c}$ be the Dirac operator acting in $L^2(\mathbb R^3{\setminus }\overline{\Omega }; \mathbb {C}^4)$ with the same boundary conditions as $T_{-\text {MIT}}$. Recall that by Proposition 3.4, we have

$$\begin{aligned} \bigl (\rho (T_{-\mathrm {MIT}}) \cap \rho (T_{-\mathrm {MIT}}^{\Omega ^c})\bigr ) \subset \mathbb {C} {\setminus } \big ( (-\infty , -m] \cup [m, \infty ) \big ), \end{aligned}$$

that the latter set is contained in $\rho (T_{\mathrm{MIT}}) \cap \rho (T_{\mathrm{MIT}}^{\Omega ^c})$, and that by Proposition 3.12 (ii) the operator $-\frac{1}{2} \beta + \mathcal {C}_{\lambda , 1/2}$ is boundedly invertible in $H^{1/2}(\partial \Omega ; \mathbb {C}^4)$ for any number $\lambda \in \rho (T_{-\mathrm {MIT}}) \cap \rho (T_{-\mathrm {MIT}}^{\Omega ^c})$.

### Proposition 4.3

Let *M* be the Weyl function corresponding to the quasi boundary triple $\{ \mathcal {G}_\Omega , \Gamma _0, \Gamma _1 \}$, assume that $\lambda \in \rho (T_{-\mathrm{MIT}}) \cap \rho (T_{-\mathrm{MIT}}^{\Omega ^c})$, and let $\mathcal {C}_{\lambda , 1/2}$ be the operator from Proposition 3.8. Then, $M(\lambda )$ admits a bounded and everywhere defined inverse in $\mathcal {G}_\Omega ^{1/2}$ which is given by

4.12 $$\begin{aligned} M(\lambda )^{-1} = P_+ \beta \left( -\frac{1}{2} \beta + \mathcal {C}_{\lambda , 1/2} \right) ^{-1} \beta P_+. \end{aligned}$$

### Proof

Observe first that $\{ \mathcal {G}_\Omega , \widehat{\Gamma }_0, \widehat{\Gamma }_1 \}$, where

4.13 $$\begin{aligned} \widehat{\Gamma }_0 = \Gamma _1 \quad \text {and} \quad \widehat{\Gamma }_1 = -\Gamma _0, \end{aligned}$$

is a quasi boundary triple for $T\subset T_{\mathrm{max}}$ such that $T \upharpoonright \ker \widehat{\Gamma }_0 = T_{-\mathrm{MIT}}$. In fact, using that $\{ \mathcal {G}_\Omega , \Gamma _0, \Gamma _1 \}$ is quasi boundary triple it follows that the abstract Green identity is satisfied by the boundary mappings in (4.13) and that the range of $(\widehat{\Gamma }_0,\widehat{\Gamma }_1)^\top$ is dense. Moreover, $T_{-\mathrm{MIT}}= T \upharpoonright \ker \widehat{\Gamma }_0$ is a self-adjoint operator by Proposition 3.4. Note that Weyl function $\widehat{M}$ corresponding to the quasi boundary triple $\{ \mathcal {G}_\Omega , \widehat{\Gamma }_0, \widehat{\Gamma }_1 \}$ is given for our choice of $\lambda \in \rho (T_{-\mathrm{MIT}}) \cap \rho (T_{-\mathrm{MIT}}^{\Omega ^c}) \subset \rho (T_{\mathrm{MIT}}) \cap \rho (T_{\mathrm{MIT}}^{{\Omega ^c}})$ by

$$\begin{aligned} \widehat{M}(\lambda ) = \widehat{\Gamma }_1 \big ( \widehat{\Gamma }_0 \upharpoonright \ker (T - \lambda ) \big )^{-1} = -(M(\lambda ))^{-1}. \end{aligned}$$

Thus, it remains to compute the value of the Weyl function $\widehat{M}(\lambda )$. We first show the explicit formula

4.14 $$\begin{aligned} \widehat{\gamma }(\lambda ) = \Phi _{\lambda , 1/2} \left( -\frac{1}{2} \beta + \mathcal {C}_{\lambda , 1/2} \right) ^{-1}\beta ,\quad \mathrm {dom}\,\widehat{\gamma }(\lambda ) = \mathcal {G}_\Omega ^{1/2}, \end{aligned}$$

for the $\gamma$-field corresponding to the quasi boundary triple $\{ \mathcal {G}_\Omega , \widehat{\Gamma }_0, \widehat{\Gamma }_1 \}$ using a similar argument as in the proof of Proposition 4.2. In fact, it is clear that $\mathrm {dom}\,\widehat{\gamma }(\lambda ) ={{\,\mathrm{ran}\,}}\widehat{\Gamma }_0= \mathcal {G}_\Omega ^{1/2}$. Next, consider $\varphi \in \mathrm {dom}\,\widehat{\gamma }(\lambda )$ and recall that $\widehat{\gamma }(\lambda ) \varphi$ is the unique solution of the boundary value problem

$$\begin{aligned} (T - \lambda ) = 0 \quad \text {and} \quad \widehat{\Gamma }_0 f_\lambda = \varphi . \end{aligned}$$

We set

$$\begin{aligned} f_\lambda = \Phi _{\lambda , 1/2} \left( -\frac{1}{2} \beta + \mathcal {C}_{\lambda , 1/2} \right) ^{-1} \beta \varphi . \end{aligned}$$

Then, we have $(T-\lambda )f_\lambda =0$, and using $P_+P_-=0$ and $\varphi \in \mathcal {G}_\Omega$, we obtain in a similar way as in the proof of Proposition 4.2 that

$$\begin{aligned} \widehat{\Gamma }_0 f_\lambda&= \Gamma _1 f_\lambda = P_+ \beta \left( \Phi _{\lambda , 1/2} \left( -\frac{1}{2} \beta + \mathcal {C}_{\lambda , 1/2} \right) ^{-1} \beta \varphi \right) \Bigg |_{\partial \Omega } \\&= P_+ \beta \left( -\frac{i}{2} (\alpha \cdot \nu ) + \frac{1}{2} \beta - \frac{1}{2} \beta + \mathcal {C}_{\lambda ,1/2} \right) \left( -\frac{1}{2} \beta + \mathcal {C}_{\lambda , 1/2} \right) ^{-1} \beta \varphi \\&= P_+ P_- \left( -\frac{1}{2} \beta + \mathcal {C}_{\lambda , 1/2} \right) ^{-1} \beta \varphi + P_+ \beta ^2 \varphi \\&=\varphi , \end{aligned}$$

which leads to (4.14).

Let us now compute the Weyl function $\widehat{M}$. Using (4.14) and Lemma 3.9, we find

$$\begin{aligned} \widehat{M}(\lambda ) \varphi&= \widehat{\Gamma }_1 \widehat{\gamma }(\lambda ) \varphi = - \Gamma _0 \widehat{\gamma }(\lambda ) \varphi \\&= -\,P_+ \left( \Phi _{\lambda , 1/2} \left( -\frac{1}{2} \beta + \mathcal {C}_{\lambda , 1/2} \right) ^{-1} \beta \varphi \right) \Bigg |_{\partial \Omega } \\&= -\,P_+ \left( -\frac{i}{2} (\alpha \cdot \nu ) + \frac{1}{2} \beta - \frac{1}{2} \beta + \mathcal {C}_{\lambda ,1/2} \right) \left( -\frac{1}{2} \beta + \mathcal {C}_{\lambda , 1/2} \right) ^{-1} \beta \varphi \\&= -\, P_+ \beta P_- \left( -\frac{1}{2} \beta + \mathcal {C}_{\lambda , 1/2} \right) ^{-1} \beta \varphi - P_+ \beta \varphi . \end{aligned}$$

Thanks to (4.2), $P_+^2=P_+$, and that $\varphi \in \mathcal {G}_\Omega$, we finally obtain

$$\begin{aligned} \widehat{M}(\lambda ) \varphi&= - P_+ \beta \left( -\frac{1}{2} \beta + \mathcal {C}_{\lambda , 1/2} \right) ^{-1} \beta P_+ \varphi - \beta P_-\varphi \\&= -\, P_+ \beta \left( -\frac{1}{2} \beta + \mathcal {C}_{\lambda , 1/2} \right) ^{-1} \beta P_+ \varphi , \end{aligned}$$

which gives (4.12). $$\square $$

Finally, we state a lemma on the invertibility of $\vartheta - M(\lambda )$ for a Hölder continuous function $\vartheta$. This result will be needed in the proofs of several results of this paper. Recall that the closure $\overline{M(\lambda )}$ of the Weyl function corresponding to the triple $\{ \mathcal {G}_\Omega , \Gamma _0, \Gamma _1 \}$ is bounded in $\mathcal {G}_\Omega$, see Proposition 4.2.

### Lemma 4.4

Let *M* be the Weyl function corresponding to the quasi boundary triple $\{ \mathcal {G}_\Omega , \Gamma _0, \Gamma _1 \}$ and let $\vartheta \in {\mathrm {Lip}}_a({\partial \Omega })$ for some $a \in (\frac{1}{2}, 1]$ be a real-valued function such that $|\vartheta (x)| \ne 1$ for all $x \in \partial \Omega$. Then, the following statements hold:

(i) For all $\lambda \in \mathbb {C} {\setminus } \mathbb {R}$, the operator $\vartheta - M(\lambda )$ has a bounded and everywhere defined inverse in $\mathcal {G}_\Omega ^{1/2}$.
(ii) For all $\lambda \in \mathbb {C} {\setminus } \mathbb {R}$, the operator $\vartheta - \overline{M(\lambda )}$ has a bounded and everywhere defined inverse in $\mathcal {G}_\Omega$.

### Proof

To prove (i) and (ii), some preparations are needed. First, due to the explicit form of the operators $M(\lambda )$ and $M(\lambda )^{-1}$ from Propositions 4.2 and 4.3 it is easy to see with the help of Proposition 3.12 that $M(\lambda )$ and $M(\lambda )^{-1}$ have bounded extensions onto $\mathcal {G}_\Omega$ given by

$$\begin{aligned} \overline{M(\lambda )} = - P_+ \left( \frac{1}{2} \beta + \mathcal {C}_{\lambda } \right) ^{-1} P_+ \end{aligned}$$

and

$$\begin{aligned} \overline{M(\lambda )^{-1}} = P_+ \beta \left( -\frac{1}{2} \beta + \mathcal {C}_{\lambda } \right) ^{-1} \beta P_+= P_+ \left( -\frac{1}{2} \beta + \beta \mathcal {C}_{\lambda }\beta \right) ^{-1} P_+, \end{aligned}$$

respectively. We claim that the operator

4.15 $$\begin{aligned} \mathcal {K}_\lambda = \vartheta \overline{M(\lambda )^{-1}} - \overline{M(\lambda )} \vartheta : \mathcal {G}_\Omega \rightarrow \mathcal {G}_\Omega ^{1/2} \end{aligned}$$

is bounded. In particular, since $\mathcal {G}_\Omega ^{1/2} = P_+(H^{1/2}(\partial \Omega ; \mathbb {C}^4))$ is compactly embedded in $\mathcal {G}_\Omega = P_+( L^2(\partial \Omega ; \mathbb {C}^4))$, this implies that $\mathcal {K}_\lambda$ is a compact operator in $\mathcal {G}_\Omega$ and that $\mathcal {K}_{\lambda , 1/2} = \mathcal {K}_\lambda \upharpoonright \mathcal {G}_\Omega ^{1/2}$ is a compact operator in $\mathcal {G}_\Omega ^{1/2}$.

To verify the boundedness of $\mathcal {K}_\lambda$ in (4.15), we note first that

4.16 $$\begin{aligned}&\vartheta \overline{M(\lambda )} - \overline{M(\lambda )} \vartheta = -P_+ \vartheta \left( \frac{1}{2} \beta + \mathcal {C}_{\lambda } \right) ^{-1} P_+ + P_+ \left( \frac{1}{2} \beta + \mathcal {C}_{\lambda } \right) ^{-1} \vartheta P_+ \nonumber \\&\quad = -\,P_+ \left( \frac{1}{2} \beta + \mathcal {C}_{\lambda , 1/2} \right) ^{-1} \left( \mathcal {C}_{\lambda } \vartheta - \vartheta \mathcal {C}_{\lambda } \right) \left( \frac{1}{2} \beta + \mathcal {C}_{\lambda } \right) ^{-1} P_+, \end{aligned}$$

which, by Propositions 3.10 and 3.12, is a bounded operator from $\mathcal {G}_\Omega$ to $\mathcal {G}_\Omega ^{1/2}$. Next, we have

4.17 $$\begin{aligned}&\overline{M(\lambda )} - \overline{M(\lambda )^{-1}} = -P_+ \left( \frac{1}{2} \beta + \mathcal {C}_{\lambda } \right) ^{-1} P_+ - P_+ \left( -\frac{1}{2} \beta + \beta \mathcal {C}_{\lambda } \beta \right) ^{-1} P_+\nonumber \\&\quad = -\,P_+ \left( -\frac{1}{2} \beta + \beta \mathcal {C}_{\lambda , 1/2} \beta \right) ^{-1} \left( \mathcal {C}_{\lambda } \beta + \beta \mathcal {C}_{\lambda } \right) \beta \left( \frac{1}{2} \beta + \mathcal {C}_{\lambda } \right) ^{-1} P_+, \end{aligned}$$

which, by Propositions 3.11 and 3.12, is also a bounded operator from $\mathcal {G}_\Omega$ to $\mathcal {G}_\Omega ^{1/2}$, as $L^2(\partial \Omega ; \mathbb {C}^4)$ is continuously embedded in $H^{-1/2}(\partial \Omega ; \mathbb {C}^4)$. Combining (4.16) with (4.17) and Lemma A.2, we conclude that the operator

$$\begin{aligned} \mathcal {K}_\lambda = \vartheta \overline{M(\lambda )^{-1}} - \overline{M(\lambda )} \vartheta = \vartheta \big ( \overline{M(\lambda )^{-1}} - \overline{M(\lambda )} \big ) + \vartheta \overline{M(\lambda )} - \overline{M(\lambda )} \vartheta \end{aligned}$$

in (4.15) is bounded.

For what follows, it is important to note that the assumptions $|\vartheta (x)| \ne 1$ for all $x\in \partial \Omega$ and $a > \frac{1}{2}$ ensure that the functions $(\vartheta ^2 - 1), (\vartheta ^2 - 1)^{-1} \in \text {Lip}_a(\partial \Omega )$ give rise to bounded and boundedly invertible multiplication operators in $\mathcal {G}_\Omega$ and $\mathcal {G}_\Omega ^{1/2}$, see Lemma A.2.

Let us now prove $\hbox {(i)}$, i.e., that $\vartheta - M(\lambda )$ has a bounded inverse in $\mathcal {G}_\Omega ^{1/2}$. Since $\vartheta - M(\lambda )$ is bounded in $\mathcal {G}_\Omega ^{1/2}$ by Lemma A.2 and Proposition 4.2, it suffices to show that this operator is bijective in $\mathcal {G}_\Omega ^{1/2}$. Note that the operator $T \upharpoonright \ker (\Gamma _1 - \vartheta \Gamma _0)$ is symmetric since $\vartheta$ is a real-valued function. (This is an immediate consequence of the abstract Green’s identity.) Hence, $\vartheta - M(\lambda )$ is injective as otherwise the symmetric operator $T \upharpoonright \ker (\Gamma _1 - \vartheta \Gamma _0)$ would have the non-real eigenvalue $\lambda$ by Theorem 2.3. Moreover, we have

4.18 $$\begin{aligned} {{\,\mathrm{ran}\,}}(\vartheta - M(\lambda ))&\supset {{\,\mathrm{ran}\,}}\big [ (\vartheta - M(\lambda )) \big ( \vartheta + M(\lambda )^{-1} \big ) \big ] \nonumber \\&= {{\,\mathrm{ran}\,}}\big [ \vartheta ^2 - 1 + \vartheta M(\lambda )^{-1} - M(\lambda ) \vartheta \big ]. \end{aligned}$$

The operators $\vartheta - M(\lambda )$ and $\vartheta + M(\lambda )^{-1} = (I_4 + \vartheta M(\lambda )) M(\lambda )^{-1}$ are both injective as otherwise one of the symmetric operators $T \upharpoonright \ker (\Gamma _1 - \vartheta \Gamma _0)$ and $T \upharpoonright \ker (\Gamma _0 + \vartheta \Gamma _1)$ would have the non-real eigenvalue $\lambda$ by Theorems 2.3 and 2.5, respectively. Therefore,

4.19 $$\begin{aligned} (\vartheta - M(\lambda )) \big ( \vartheta + M(\lambda )^{-1} \big ) = (\vartheta ^2 - 1) \left[ 1 + \frac{1}{\vartheta ^2 - 1} \mathcal {K}_{\lambda , 1/2} \right] \end{aligned}$$

is injective, and since $\mathcal {K}_{\lambda , 1/2} = \mathcal {K}_\lambda \upharpoonright \mathcal {G}_\Omega ^{1/2}$ is a compact operator in $\mathcal {G}_\Omega ^{1/2}$, we conclude from Fredholm’s alternative and the bijectivity of $\vartheta ^2 - 1$ in $\mathcal {G}_\Omega ^{1/2}$ that the operator (4.19) is bijective in $\mathcal {G}_\Omega ^{1/2}$. From (4.18), we conclude $\mathcal {G}_\Omega ^{1/2} \subset {{\,\mathrm{ran}\,}}(\vartheta - M(\lambda ))$ and hence we have shown that $\vartheta - M(\lambda )$ is bijective in $\mathcal {G}_\Omega ^{1/2}$.

Let us now focus on $\hbox {(ii)}$. The proof that $\vartheta - \overline{M(\lambda )}$ has a bounded inverse in $\mathcal {G}_\Omega$ follows the same lines as above. The only difference is in the argument that $\vartheta - \overline{M(\lambda )}$ and $\vartheta + \overline{M(\lambda )^{-1}}$ are injective. To see this for, e.g., $\vartheta - \overline{M(\lambda )}$, assume that $\varphi \in \mathcal {G}_\Omega$ is such that $(\vartheta - \overline{M(\lambda )}) \varphi = 0$. Then,

$$\begin{aligned} 0 = \big ( \vartheta + \overline{M(\lambda )^{-1}} \big )\big (\vartheta - \overline{M(\lambda )}\big ) \varphi = (\vartheta ^2 - 1) \left[ 1 + \frac{1}{\vartheta ^2 - 1} \widetilde{\mathcal {K}}_\lambda \right] \varphi \end{aligned}$$

with

$$\begin{aligned} \widetilde{\mathcal {K}}_\lambda := \overline{M(\lambda )^{-1}} \vartheta - \vartheta \overline{M(\lambda )} = \big (\overline{M(\lambda )^{-1}} - \overline{M(\lambda )} \big ) \vartheta + \overline{M(\lambda )} \vartheta - \vartheta \overline{M(\lambda )}. \end{aligned}$$

From the second equality in the last line, we conclude in the same way as in the proof of (4.15) from (4.16) and (4.17) that $\widetilde{\mathcal {K}}_\lambda$ maps $\mathcal {G}_\Omega$ into $\mathcal {G}_{\Omega }^{1/2}$. Using this and the bijectivity of $\vartheta ^2 - 1$ in $\mathcal {G}_\Omega ^{1/2}$, we get

$$\begin{aligned} \varphi = -\frac{1}{\vartheta ^2 - 1} \widetilde{\mathcal {K}}_\lambda \varphi \in \mathcal {G}_\Omega ^{1/2}, \end{aligned}$$

that is, $\varphi \in \ker (\vartheta - M(\lambda ))$. By the above considerations this implies $\varphi = 0$, i.e., $\vartheta - \overline{M(\lambda )}$ is injective. Similarly, one shows that also $\vartheta + \overline{M(\lambda )^{-1}}$ is injective. To show that $\vartheta - \overline{M(\lambda )}$ is surjective, one can use a similar argument as in (4.18) with $M(\lambda )$ and $M(\lambda )^{-1}$ replaced by $\overline{M(\lambda )}$ and $\overline{M(\lambda )^{-1}}$, respectively. The details are left to the reader. $\square$

## Dirac Operators on Domains

This section contains the main results of this paper. First, in Sect. 5.1 we introduce Dirac operators $A_\vartheta$ on $\Omega$ with boundary conditions of the form

5.1 $$\begin{aligned} \vartheta P_+ f|_{\partial \Omega } = P_+ \beta f|_{\partial \Omega } \end{aligned}$$

for a real-valued Hölder continuous function $\vartheta : \partial \Omega \rightarrow \mathbb {R}$ of order $a > \frac{1}{2}$ and $P_+$ given by (4.1). Using the quasi boundary triple $\{ \mathcal {G}_\Omega , \Gamma _0, \Gamma _1 \}$ from Theorem 4.1 we show that $A_\vartheta$ is self-adjoint if $\vert \vartheta (x)\vert \ne 1$ for all $x\in \partial \Omega$. We also obtain a Krein-type resolvent formula and some qualitative spectral properties of $A_\vartheta$. In Sect. 5.2, we sketch how Dirac operators $A_{[\omega ]}$ with boundary conditions of the form

$$\begin{aligned} P_+ f|_{\partial \Omega } = \omega P_+ \beta f|_{\partial \Omega } \end{aligned}$$

for a real-valued Hölder continuous function $\omega : \partial \Omega \rightarrow \mathbb {R}$ of order $a > \frac{1}{2}$ can be handled with similar arguments. Finally, in Sect. 5.3 we relate the operators $A_\vartheta$ to Dirac operators $B_{\eta , \tau }$ with singular $\delta$–shell potentials of the form (1.4). This relation allows to translate results for $B_{\eta , \tau }$ to $A_\vartheta$, and vice versa.

Throughout this section, let $\Omega$ be a bounded or unbounded domain in $\mathbb {R}^3$ with a compact $C^2$-smooth boundary, and denote by $\nu$ the normal vector field at $\partial \Omega$ pointing outwards of $\Omega$.

### Self-Adjointness and Spectral Properties of $A_\vartheta$

We start with the rigorous mathematical definition of the Dirac operator $A_\vartheta$ with boundary conditions (5.1). We shall use the quasi boundary triple $\{ \mathcal {G}_\Omega , \Gamma _0, \Gamma _1 \}$ from Theorem 4.1 in the next definition.

#### Definition 5.1

Let $a \in (\frac{1}{2}, 1 ]$ and let $\vartheta \in \mathrm{Lip}_a({\partial \Omega })$ be real-valued. We define $A_\vartheta = T \upharpoonright \ker (\Gamma _1 - \vartheta \Gamma _0)$, which in a more explicit form is given by

5.2 $$\begin{aligned} A_\vartheta f&= (-i \alpha \cdot \nabla + m \beta ) f, \nonumber \\ \mathrm {dom}\,A_\vartheta&= \bigl \{ f \in H^1(\Omega ; \mathbb {C}^4): \vartheta P_+ f|_{\partial \Omega } = P_+ \beta f|_{\partial \Omega } \bigr \}. \end{aligned}$$

#### Remark 5.2

The boundary conditions in (5.1) are the 3D analogue of the boundary conditions used in [22]. In fact, let $\Omega \subset \mathbb {R}^2$ be a bounded $C^2$-domain. In [22], the boundary conditions

5.3 $$\begin{aligned} \big [ I_2 + i \sigma _3 (\sigma \cdot \nu ) \cos \eta - \sin \eta \sigma _3 \big ] u|_{\partial \Omega } = 0 \end{aligned}$$

for $C^1$-functions $\eta : \partial \Omega \rightarrow \mathbb {R}$ with $\cos [\eta (x)] \notin \{ 0, 1 \}$ for all $x \in \partial \Omega$ are treated. Here, $\sigma = (\sigma _1, \sigma _2)$ and $\sigma _3$ are the Pauli spin matrices in (1.7), $\nu = (\nu _1, \nu _2)$ is the normal vector field at $\partial \Omega$, and $\sigma \cdot \nu = \sigma _1 \nu _1 + \sigma _2 \nu _2$. To see that (5.3) is equivalent to the boundary conditions in [22], one has to note that $\sigma \cdot \mathbf {t} = -i \sigma _3 (\sigma \cdot \nu )$, where $\mathbf {t} = (-\nu _2, \nu _1)$ is the tangential vector at $\partial \Omega$. We use the splitting

$$\begin{aligned} u|_{\partial \Omega } = Q_+ u|_{\partial \Omega } + Q_- u|_{\partial \Omega },\qquad Q_\pm =\frac{1}{2} (I_2 \pm i \sigma _3 (\sigma \cdot \nu )) , \end{aligned}$$

and remark that $Q_\pm$ is the 2D-analogue of $P_\pm$ from (4.1). Hence, we can rewrite (5.3) as

$$\begin{aligned} \big [ I_2 + i \sigma _3 (\sigma \cdot \nu ) \cos \eta - \sin \eta \sigma _3 \big ] Q_+ u|_{\partial \Omega } = -\big [ I_2 + i \sigma _3 (\sigma \cdot \nu ) \cos \eta - \sin \eta \sigma _3 \big ] Q_- u|_{\partial \Omega }. \end{aligned}$$

With the help of the relations $i \sigma _3 (\sigma \cdot \nu ) Q_\pm = \pm Q_\pm$ and $Q_- = \sigma _3 Q_+ \sigma _3$, we find that (5.3) is equivalent to

$$\begin{aligned} \big [ I_2 + \cos \eta I_2 - \sin \eta \sigma _3 \big ] Q_+ u|_{\partial \Omega }&= \big [ I_2 + i \sigma _3 (\sigma \cdot \nu ) \cos \eta - \sin \eta \sigma _3 \big ] Q_+ u|_{\partial \Omega } \\&= -\,\big [ I_2 + i \sigma _3 (\sigma \cdot \nu ) \cos \eta - \sin \eta \sigma _3 \big ] Q_- u|_{\partial \Omega } \\&= -\,\big [ I_2 - \cos \eta I_2 - \sin \eta \sigma _3 \big ] Q_- u|_{\partial \Omega } \\&= -\,\big [ (1-\cos \eta ) \sigma _3 - \sin \eta I_2 \big ] Q_+ \sigma _3 u|_{\partial \Omega }. \end{aligned}$$

By multiplying this equation with

$$\begin{aligned} -\big [ (1-\cos \eta ) \sigma _3 - \sin \eta I_2 \big ]^{-1} = \frac{1}{2\cos \eta (1-\cos \eta )} \big [ (1-\cos \eta ) \sigma _3 + \sin \eta I_2 \big ], \end{aligned}$$

which exists since $\cos [\eta (x)] \notin \{ 0, 1 \}$ is assumed, we see that (5.3) is equivalent to

$$\begin{aligned} \frac{\sin (2 \eta )}{2 \cos \eta (1 - \cos \eta )} Q_+ u|_{\partial \Omega } = Q_+ \sigma _3 u|_{\partial \Omega }, \end{aligned}$$

which is the 2D analogue of the boundary conditions in (5.2) for the parameter $\vartheta = \frac{\sin (2 \eta )}{2 \cos \eta (1 - \cos \eta )}$.

It follows immediately from the abstract Green’s identity that $A_\vartheta$ is symmetric for any real-valued function $\vartheta$. In order to prove self-adjointness, we shall use Theorem 2.3, which also leads to a resolvent formula in terms of the resolvent of the MIT bag operator $T_\text {MIT}$ in (3.4) and the $\gamma$-field and Weyl function. We note that in (5.33) an explicit formula for $(T_\text {MIT} - \lambda )^{-1}$ is shown.

#### Theorem 5.3

Let $a \in (\frac{1}{2}, 1 ]$ and let $\vartheta \in {\mathrm {Lip}}_a({\partial \Omega })$ be a real-valued function such that $|\vartheta (x)| \ne 1$ for all $x \in \partial \Omega$. Moreover, let $\gamma$ and *M* be as in Proposition 4.2. Then, the operator $A_{\vartheta }$ in (5.2) is self-adjoint in $L^2(\Omega ; \mathbb {C}^4)$ and the resolvent formula

$$\begin{aligned} (A_\vartheta -\lambda )^{-1} = \big (T_{\mathrm{MIT}} - \lambda \big )^{-1} + \gamma (\lambda ) \big ( \vartheta - M(\lambda ) \big )^{-1} \gamma (\overline{\lambda })^* \end{aligned}$$

holds for all $\lambda \in \rho (A_{\vartheta })\cap \rho (T_{\mathrm{MIT}})$.

#### Proof

As mentioned above, it follows from the abstract Green’s identity that the operator $A_\vartheta$ is symmetric. Thus, for the self-adjointness it suffices to check that ${{\,\mathrm{ran}\,}}(A_\vartheta - \lambda ) = L^2(\Omega ; \mathbb {C}^4)$ holds for some, and hence for all $\lambda \in \mathbb {C_\pm }$.

Let $f \in L^2(\Omega ; \mathbb {C}^4)$ and $\lambda \in \mathbb {C} {\setminus } \mathbb {R}$. According to Theorem 2.3 (ii), we would have $f \in {{\,\mathrm{ran}\,}}(A_\vartheta - \lambda )$ if we can show that $\gamma (\overline{\lambda })^* f \in {{\,\mathrm{ran}\,}}(\vartheta - M(\lambda ))$ holds. In fact, from $\gamma (\overline{\lambda })^* = \Gamma _1 \big (T_{\mathrm{MIT}} - \lambda \big )^{-1}$ and $\mathrm {dom}\,T_{\mathrm{MIT}} \subset H^1(\Omega ; \mathbb {C}^4)$ we obtain $\gamma (\overline{\lambda })^* f \in \mathcal {G}_\Omega ^{1/2}$. Furthermore, by Lemma 4.4 (i) the operator $\vartheta - M(\lambda )$ is bijective in $\mathcal {G}_\Omega ^{1/2}$ and hence $\gamma (\overline{\lambda })^* f \in {{\,\mathrm{ran}\,}}(\vartheta - M(\lambda ))$, that is, $f \in {{\,\mathrm{ran}\,}}(A_\vartheta - \lambda )$. As *f* was arbitrary we get ${{\,\mathrm{ran}\,}}(A_\vartheta - \lambda ) = L^2(\Omega ; \mathbb {C}^4)$ for $\lambda \in \mathbb {C} {\setminus } \mathbb {R}$, so that $A_\vartheta$ is self-adjoint in $L^2(\Omega ; \mathbb {C}^4)$. Finally, the formula for the resolvent of $A_\vartheta$ follows from Theorem 2.3 and (4.6). $\square$

Next, we discuss the basic spectral properties of the operator $A_\vartheta$. Since these are of a very different nature whether $\Omega$ is bounded or unbounded, the two cases are treated separately. Assume first that $\Omega$ is an unbounded $C^2$-domain with compact boundary. The proof of (ii) is based on the same argument as the proof of [16, Proposition 3.8].

#### Theorem 5.4

Let $\Omega$ be the complement of a bounded $C^2$-domain, let $a \in (\frac{1}{2}, 1]$, let $\vartheta \in {\mathrm {Lip}}_a({\partial \Omega })$ be a real-valued function such that $|\vartheta (x)| \ne 1$ for all $x \in \partial \Omega$, and let $A_\vartheta$ be defined by (5.2). Then, the following statements hold:

(i) $$\sigma _{\mathrm{ess}}(A_\vartheta ) = (-\infty , - m] \cup [m, \infty )$$.
(ii) The number of discrete eigenvalues of $A_\vartheta$ is finite.
(iii) $\lambda \in \sigma _{\mathrm{p}}(A_\vartheta ) \cap (-m, m)$ if and only if $0 \in \sigma _\mathrm{p}(\vartheta - M(\lambda ))$.

#### Proof

We first deal with (i). Let $\gamma$ and *M* be the $\gamma$-field and the Weyl function corresponding to the quasi boundary triple $\{ \mathcal {G}_\Omega , \Gamma _0, \Gamma _1 \}$, respectively, from Proposition 4.2, and let $\overline{\gamma (\lambda )} \in \mathcal {B}(\mathcal {G}_\Omega , L^2(\Omega ; \mathbb {C}^4))$ and $\overline{M(\lambda )} \in \mathcal {B}(\mathcal {G}_\Omega )$ be the closures of $\gamma (\lambda )$ and $M(\lambda )$, $\lambda \in \rho (T_{\mathrm{MIT}})$. For $\lambda \in \rho (A_{\vartheta })\cap \rho (T_{\mathrm{MIT}})$ the resolvent formula in Theorem 5.3 can be written in the form

5.4 $$\begin{aligned} (A_\vartheta -\lambda )^{-1} - \big (T_{\mathrm{MIT}} - \lambda \big )^{-1} = \overline{\gamma (\lambda )} \big ( \vartheta - \overline{M(\lambda )} \big )^{-1} \gamma (\overline{\lambda })^*. \end{aligned}$$

By Proposition 4.2 (ii), the operator $\gamma (\overline{\lambda })^*$ is compact from $L^2(\Omega ; \mathbb {C}^4)$ to $\mathcal {G}_\Omega$. Furthermore, by Lemma 4.4 the inverse $( \vartheta - \overline{M(\lambda )})^{-1}$ is bounded in $\mathcal {G}_\Omega$. Since $\overline{\gamma (\lambda )}: \mathcal {G}_\Omega \rightarrow L^2(\Omega ; \mathbb {C}^4)$ is bounded, we deduce that the right-hand side in (5.4) is compact in $L^2(\Omega ; \mathbb {C}^4)$ and hence the same holds for the left-hand side. Together with Proposition 3.3 (iii) this implies

$$\begin{aligned} \sigma _{\mathrm{ess}}(A_\vartheta ) = \sigma _{\mathrm{ess}}(T_{\mathrm{MIT}}) = (-\infty , -m] \cup [m, \infty ). \end{aligned}$$

To verify assertion (ii), consider the quadratic form

$$\begin{aligned} \mathfrak {a}[f] = \Vert A_\vartheta f \Vert _\Omega ^2, \qquad \mathrm {dom}\,\mathfrak {a} = \mathrm {dom}\,A_\vartheta . \end{aligned}$$

Since $A_\vartheta$ is a self-adjoint operator, it follows that $\mathfrak {a}$ is a closed, nonnegative form and by [44, Theorem VI 2.1] the unique self-adjoint operator representing this form is $A_\vartheta ^2$. Note that the number of eigenvalues (counted with multiplicities) of $A_\vartheta$ in the gap of the essential spectrum $(-m, m)$ is equal to the number of eigenvalues of $A_\vartheta ^2$ below $m^2$ (counted with multiplicities).

To estimate the number of eigenvalues of $A_\vartheta ^2$ with the help of the quadratic form $\mathfrak {a}$, let $0< r < R$ such that ${\partial \Omega } \subset B(0, r)$ and choose real-valued functions $g_1, g_2 \in C^\infty (\overline{\Omega }; \mathbb {C})$ with the properties

$$\begin{aligned} 0 \le g_1, g_2 \le 1,\,\, g_1 \upharpoonright (B(0, r) \cap \Omega ) \equiv 1,\,\, g_2 \upharpoonright B(0, R)^c \equiv 1, \quad \text {and} \quad g_1^2 + g_2^2 \equiv 1. \end{aligned}$$

Note that the properties of $g_1$ and $g_2$ imply that the mapping

$$\begin{aligned} U: L^2(\Omega ; \mathbb {C}^4) \rightarrow L^2\big ( \Omega \cap B(0, R); \mathbb {C}^4 \big ) \oplus L^2(\mathbb {R}^3 {\setminus } \overline{B(0, r)}; \mathbb {C}^4), \quad U f = g_1 f \oplus g_2 f, \end{aligned}$$

is an isometry. Our next goal is to rewrite the form $\mathfrak {a}$ as a sesquilinear form in $L^2( \Omega \cap B(0, R); \mathbb {C}^4) \oplus L^2(\mathbb {R}^3 {\setminus } \overline{B(0, r)}; \mathbb {C}^4)$. For that, we will often identify functions defined in $\Omega$ with their restrictions onto $\Omega \cap B(0,R)$ or onto $\mathbb {R}^3 {\setminus } \overline{B(0,r)}$ and we also identify functions on $\Omega \cap B(0,R)$ or $\mathbb {R}^3 {\setminus } \overline{B(0,r)}$ with their extensions by zero onto $\Omega$. In both cases, we will use the same letters for the restrictions and the extended functions.

Let $f \in \mathrm {dom}\,\mathfrak {a} = \mathrm {dom}\,A_\vartheta$ be fixed. Then, also $g_1 f, g_2 f \in \mathrm {dom}\,\mathfrak {a}$. Using the relation

$$\begin{aligned} A_\vartheta (g_j f) = g_j A_\vartheta f - i (\alpha \cdot \nabla g_j) f,\quad j=1,2, \end{aligned}$$

we find that

$$\begin{aligned} \mathfrak {a}[g_j f]&= \big ( g_j A_\vartheta f -i (\alpha \cdot \nabla g_j) f, g_j A_\vartheta f -i (\alpha \cdot \nabla g_j) f \big )_\Omega \\&= \big ( g_j^2 A_\vartheta f, A_\vartheta f \big )_\Omega + \big \Vert (\alpha \cdot \nabla g_j) f \big \Vert _\Omega ^2 + \text {Re}\, \big ( A_\vartheta f, (-i \alpha \cdot \nabla (g_j^2)) f \big )_\Omega . \end{aligned}$$

Note that (1.8) implies $(\alpha \cdot \nabla g_j)^2 = |\nabla g_j|^2 I_4$, which gives

$$\begin{aligned} \big \Vert (\alpha \cdot \nabla g_j) f \big \Vert _\Omega ^2 = \big ( (\alpha \cdot \nabla g_j)^2 f, f \big )_\Omega = \big ( |\nabla g_j|^2 f,f \big )_\Omega . \end{aligned}$$

Moreover, since $g_1^2 + g_2^2 \equiv 1$, we have

$$\begin{aligned} \big ( A_\vartheta f, (\alpha \cdot \nabla (g_1^2)) f \big )_\Omega + \big ( A_\vartheta f, (\alpha \cdot \nabla (g_2^2)) f \big )_\Omega = \big ( A_\vartheta f, (\alpha \cdot \nabla (g_1^2 + g_2^2)) f \big )_\Omega = 0. \end{aligned}$$

We set $V=|\nabla g_1|^2 + |\nabla g_2|^2$ and conclude

5.5 $$\begin{aligned} \mathfrak {a}[f]&= \big ( (g_1^2 + g_2^2) A_\vartheta f, A_\vartheta f \big )_\Omega \nonumber \\&= \mathfrak {a}[g_1 f] - \big ( |\nabla g_1|^2 f,f \big )_\Omega + \mathfrak {a}[g_2 f] - \big ( |\nabla g_2|^2 f,f \big )_\Omega \nonumber \\&\qquad - \text {Re}\, \big ( A_\vartheta f, (\alpha \cdot \nabla (g_1^2 + g_2^2)) f \big )_\Omega \nonumber \\&= \mathfrak {a}[g_1 f] - \big ( V (g_1^2+g_2^2) f, f \big )_\Omega + \mathfrak {a}[g_2 f] \nonumber \\&= \mathfrak {a}[g_1 f] - \big ( V g_1 f, g_1f \big )_\Omega + \mathfrak {a}[g_2 f] - \big ( V g_2 f,g_2 f \big )_\Omega \nonumber \\&=: \mathfrak {b}_1[g_1 f] + \mathfrak {b}_2[g_2 f], \end{aligned}$$

where $\mathfrak {b}_1$ and $\mathfrak {b}_2$ are the semibounded sesquilinear forms in $L^2(\Omega \cap B(0, R); \mathbb {C}^4)$ and $L^2(\mathbb {R}^3 {\setminus } \overline{B(0, r)}; \mathbb {C}^4)$ given by

$$\begin{aligned} \mathfrak {b}_1[h] = \mathfrak {a}[h] - \big ( V h, h \big )_{\Omega \cap B(0, R)}, \quad \mathrm {dom}\,\mathfrak {b}_1 = \{ h \in \mathrm {dom}\,A_\vartheta : \text {supp}\, h \subset \overline{B(0, R)} \}, \end{aligned}$$

and

$$\begin{aligned} \mathfrak {b}_2[h] = \mathfrak {a}[h] - \big ( V h, h \big )_{\mathbb {R}^3 {\setminus } \overline{B(0,r)}}, \quad \mathrm {dom}\,\mathfrak {b}_2 = H^1_0(\mathbb {R}^3 {\setminus } \overline{B(0, r)}; \mathbb {C}^4), \end{aligned}$$

respectively.

In the following, let us have a closer look at $\mathfrak {b}_1$ and $\mathfrak {b}_2$. First, we note that with the aid of (3.3) and (1.8) one has for $h \in C^\infty _0(\mathbb {R}^3 {\setminus } \overline{B(0, r)}; \mathbb {C}^4)$ that

$$\begin{aligned} \mathfrak {b}_2[h]&= \Vert (-i \alpha \cdot \nabla + m \beta ) h \Vert _{\mathbb {R}^3 {\setminus } \overline{B(0, r)}}^2 - ( V h, h )_{\mathbb {R}^3 {\setminus } \overline{B(0,r)}}\\&= \big ( (-i \alpha \cdot \nabla + m \beta )^2 h, h \big )_{\mathbb {R}^3 {\setminus } \overline{B(0, r)}} - ( V h, h )_{\mathbb {R}^3 {\setminus } \overline{B(0,r)}}\\&= \big ( (-\Delta + m^2) h, h \big )_{\mathbb {R}^3 {\setminus } \overline{B(0, r)}} - ( V h, h )_{\mathbb {R}^3 {\setminus } \overline{B(0,r)}}\\&= \Vert \nabla h \Vert _{\mathbb {R}^3 {\setminus } \overline{B(0, r)}} + m^2 \Vert h\Vert _{\mathbb {R}^3 {\setminus } \overline{B(0, r)}}^2 - ( V h, h)_{\mathbb {R}^3 {\setminus } \overline{B(0,r)}}. \\ \end{aligned}$$

By density, this extends to

$$\begin{aligned} \mathfrak {b}_2[h] = \Vert \nabla h \Vert _{\mathbb {R}^3 {\setminus } \overline{B(0, r)}} + m^2 \Vert h\Vert _{\mathbb {R}^3 {\setminus } \overline{B(0, r)}}^2 - ( V h, h)_{\mathbb {R}^3 {\setminus } \overline{B(0,r)}} \end{aligned}$$

for all $h \in H^1_0(\mathbb {R}^3 {\setminus } \overline{B(0, r)}; \mathbb {C}^4) = \mathrm {dom}\,\mathfrak {b}_2$, i.e., $\mathfrak {b}_2$ is the closed semibounded form associated with the self-adjoint operator $B_2 := -\Delta ^D + m^2 - V$, where $-\Delta ^D$ is the self-adjoint Dirichlet Laplacian in $\mathbb {R}^3 {\setminus } \overline{B(0, r)}$ and $V = |\nabla g_1|^2 + |\nabla g_2|^2$ is compactly supported in $\overline{B(0, R)} {\setminus } B(0, r)$ due to the construction of $g_1$ and $g_2$. Thus, $B_2$ has only finitely many eigenvalues below $m^2$; for a proof see, e.g., [16, Proof of Proposition 3.8].

Next, we claim that $\mathfrak {b}_1$ is closed. In fact, let $(h_n)$ be a sequence in $\mathrm {dom}\,\mathfrak {b}_1$ and let $h \in L^2(\Omega \cap B(0, R))$ such that

$$\begin{aligned} \mathfrak {b}_1[h_n - h_m] \rightarrow 0 \quad \text {and} \quad \Vert h_n - h \Vert _{\Omega \cap B(0, R)} \rightarrow 0, \quad \text {as }m, n \rightarrow \infty . \end{aligned}$$

By the definition of $\mathfrak {b}_1$, this implies that $\mathfrak {a}[h_n - h_m] \rightarrow 0$ and $\Vert h_n - h \Vert _\Omega \rightarrow 0$, as $m, n \rightarrow \infty$. As $\mathfrak {a}$ is closed we have $h \in \mathrm {dom}\,\mathfrak {a}= \mathrm {dom}\,A_\vartheta$ and $\mathfrak a[h-h_n]\rightarrow 0$ as $n\rightarrow \infty$. Moreover, it follows from $\Vert h_n - h \Vert _\Omega \rightarrow 0$ that $\text {supp}\, h \subset \overline{B(0, R)}$. Hence, $h \in \mathrm {dom}\,\mathfrak {b}_1$ and $\mathfrak b_1[h-h_n]\rightarrow 0$ as $n\rightarrow \infty$; thus, $\mathfrak {b}_1$ is closed. The semibounded self-adjoint operator $B_1$ associated with $\mathfrak {b}_1$ defined on $\mathrm {dom}\,B_1 \subset \mathrm {dom}\,\mathfrak {b}_1 \subset H^1(\Omega \cap B(0, R); \mathbb {C}^4)$ has a compact resolvent in $L^2(\Omega \cap B(0, R); \mathbb {C}^4)$, which implies that the spectrum of $B_1$ is purely discrete and accumulates only to $\infty$.

By combining the above considerations, we are now prepared to show the claim of assertion (ii). First, we have by (5.5) for $f \in \mathrm {dom}\,\mathfrak {a}$

$$\begin{aligned} \frac{\mathfrak {a}[f]}{\Vert f \Vert _\Omega ^2} = \frac{(\mathfrak {b}_1 \oplus \mathfrak {b}_2)[U f]}{\Vert U f \Vert _{L^2(\Omega \cap B(0, R); \mathbb {C}^4) \oplus L^2(\mathbb {R}^3 {\setminus } \overline{B(0, r)}; \mathbb {C}^4)}^2}, \end{aligned}$$

where it was used that *U* is an isometry, and $U (\mathrm {dom}\,\mathfrak {a}) \subset \mathrm {dom}\,(\mathfrak {b}_1 \oplus \mathfrak {b}_2)$. Hence, it follows from the min–max principle that the number of eigenvalues of $A_\vartheta ^2$ below $m^2$ is less or equal to the number of eigenvalues of the operator $B_1 \oplus B_2$ associated with $\mathfrak {b}_1 \oplus \mathfrak {b}_2$ below $m^2$. As we have seen above, the number of eigenvalues of $B_1$ and $B_2$ below $m^2$ is finite. Hence, also the number of eigenvalues of $B_1 \oplus B_2$ below $m^2$ is finite. This shows that the number of eigenvalues of $A_\vartheta ^2$ below $m^2$ is finite, which yields the claimed result.

Finally, item (iii) is an immediate consequence of Theorem 2.3 (i). $\square$

If $\Omega$ is a bounded $C^2$-domain, then $\mathrm {dom}\,A_\vartheta \subset H^1(\Omega ; \mathbb {C}^4)$ is compactly embedded in $L^2(\Omega ; \mathbb {C}^4)$ and hence the spectrum of $A_\vartheta$ is purely discrete. It is clear that the Birman–Schwinger principle from Theorem 2.3 can be used to detect discrete eigenvalues of $A_\vartheta$ that belong to $\rho (T_{\mathrm{MIT}})$. The next result, which is a direct consequence of Propositions 2.4 and 3.2, goes beyond the standard Birman–Schwinger principle in two ways: First, it allows to detect eigenvalues of $A_\vartheta$ that may be eigenvalues of $T_{\mathrm{MIT}}$ at the same time, and second, it enables to use the explicit expression for the values $M(\lambda )$ of the Weyl function in Proposition 4.2 in terms of integral operators (which we have available only for $\lambda \in \mathbb {C} {\setminus } ( (-\infty , -m] \cup [m, \infty ) )$).

#### Proposition 5.5

Let $\Omega$ be a bounded $C^2$-smooth domain, let $a \in (\frac{1}{2}, 1 ]$, let $\vartheta \in {\mathrm {Lip}}_a({\partial \Omega })$ be a real-valued function such that $|\vartheta (x)| \ne 1$ for all $x \in \partial \Omega$, and let $A_\vartheta$ be defined by (5.2). Then, $\sigma (A_\vartheta ) = \sigma _{\mathrm{disc}}(A_\vartheta )$ and $\lambda$ is an eigenvalue of $A_\vartheta$ if and only if there exists $\varphi \in \mathcal {G}_\Omega ^{1/2}$ such that

$$\begin{aligned} \lim _{\varepsilon \searrow 0} i \varepsilon \big ( M(\lambda + i \varepsilon ) - \vartheta \big )^{-1} \varphi \ne 0, \end{aligned}$$

where *M* is the Weyl function corresponding to the quasi boundary triple $\{ \mathcal {G}_\Omega , \Gamma _0, \Gamma _1 \}$ from Proposition 4.2.

#### Remark 5.6

In Theorem 5.4 we discuss the spectral properties of $A_\vartheta$ for unbounded domains $\Omega$, but we do not address the question of eigenvalues which are embedded in $\sigma _{\mathrm{ess}}(A_\vartheta ) = (-\infty , -m] \cup [m, \infty )$. In fact, if the domain $\Omega$ is connected, then it is not difficult to show that $A_\vartheta$ has no embedded eigenvalues in $\mathbb {R} {\setminus } [-m, m]$; this can be done in the same way as in [5, Theorem 3.7], see also the discussion of this result. If $\Omega$ is not connected, then there exist a bounded set $\Omega _1$ and an unbounded connected domain $\Omega _2$ such that $\Omega = \Omega _1 \cup \Omega _2$. This implies that also $A_\vartheta$ decomposes as $A_\vartheta = A_{\vartheta , 1} \oplus A_{\vartheta , 2}$, where $A_{\vartheta , j}$ is a self-adjoint operator of the form (5.2) in $L^2(\Omega _j; \mathbb {C}^4)$, $j \in \{ 1, 2 \}$. By the same reasoning as above $A_{\vartheta , 2}$ has no eigenvalues in $\mathbb {R} {\setminus } [-m, m]$. Therefore, the embedded eigenvalues of $A_\vartheta$ are those of $A_{\vartheta , 1}$, which can be found with the help of Proposition 5.5.

Next, we compare the differences of powers of the resolvents of $A_\vartheta$ and $T_{\mathrm{MIT}}$ and show that these operators belong to certain weak Schatten–von Neumann ideals. In the proof of this result, we will make several times use of

5.6 $$\begin{aligned} S T \in \mathfrak {S}_{r, \infty } \quad \text {for} \quad S \in \mathfrak {S}_{p, \infty }, \quad T \in \mathfrak {S}_{q, \infty }, \quad \text {and} \quad \frac{1}{r} = \frac{1}{p} + \frac{1}{q}. \end{aligned}$$

Moreover, for holomorphic operator functions $A(\cdot ), B(\cdot ), C(\cdot )$ the formula

5.7 $$\begin{aligned} \frac{\text {d}^m}{\text {d} \lambda ^m} \big ( A(\lambda ) B(\lambda ) C(\lambda ) \big ) = \sum _{p+q+r = m} \frac{m!}{p! q! r!} \frac{\text {d}^p}{\text {d} \lambda ^p} A(\lambda ) \frac{\text {d}^q}{\text {d} \lambda ^q} B(\lambda ) \frac{\text {d}^r}{\text {d} \lambda ^r} C(\lambda ) \end{aligned}$$

(see, e.g., [19, equation (2.7)]) will be employed several times. Furthermore, if the operator function $A(\cdot )$ is holomorphic and invertible with bounded everywhere defined inverses, then also $A(\cdot )^{-1}$ is holomorphic and one has

5.8 $$\begin{aligned} \frac{\text {d}}{\text {d} \lambda } \big ( A(\lambda )^{-1} \big ) = -A(\lambda )^{-1} \left( \frac{\text {d}}{\text {d} \lambda } A(\lambda ) \right) A(\lambda )^{-1}; \end{aligned}$$

cf. [19, equation (2.8)]. The proof of the following theorem is based on the result of Lemma 3.14 and on the same strategy as in [19] or in [10, Theorem 4.6]. Hence, we have to assume some additional smoothness of $\partial \Omega$.

#### Theorem 5.7

Let $\Omega$ be a $C^2$-domain with compact boundary, let $T_{\mathrm{MIT}}$ be the MIT bag operator in (3.4), let $a \in (\frac{1}{2}, 1]$, let $\vartheta \in {\mathrm {Lip}}_a({\partial \Omega })$ be a real-valued function such that $|\vartheta (x)| \ne 1$ for all $x \in \partial \Omega$, and let $A_\vartheta$ be defined by (5.2). Moreover, let $l \in \mathbb {N}$ and, if $l>2$, assume that $\Omega$ has a $C^l$-smooth boundary. Then,

$$\begin{aligned} (A_\vartheta - \lambda )^{-l} - (T_{\mathrm{MIT}} - \lambda )^{-l} \in \mathfrak {S}_{2/l, \infty }\bigl (L^2(\Omega ; \mathbb {C}^4)\bigr ) \end{aligned}$$

holds for all $\lambda \in \mathbb {C} {\setminus } \mathbb {R}$.

#### Proof

Let $\lambda \in \mathbb {C} {\setminus } \mathbb {R}$ be fixed. By Proposition 4.2, we have

$$\begin{aligned} \gamma (\lambda ) = \Phi _{\lambda , 1/2} \left( \frac{1}{2} \beta + \mathcal {C}_{\lambda , 1/2} \right) ^{-1}. \end{aligned}$$

Hence, using Propositions 3.6 and 3.12 we find

5.9 $$\begin{aligned} \overline{\gamma (\lambda )} = \Phi _\lambda \left( \frac{1}{2} \beta + \mathcal {C}_\lambda \right) ^{-1}. \end{aligned}$$

In a similar way, one gets

5.10 $$\begin{aligned} \overline{M(\lambda )} = - P_+ \left( \frac{1}{2} \beta + \mathcal {C}_\lambda \right) ^{-1} P_+. \end{aligned}$$

With the resolvent formula from Theorem 5.3 and (5.7), we obtain

5.11 $$\begin{aligned} (A_\vartheta - \lambda )^{-l} - (&T_{\mathrm{MIT}} - \lambda )^{-l} = \frac{1}{(l-1)!} \frac{\text {d}^{l-1}}{\text {d} \lambda ^{l-1}} \big ( (A_\vartheta - \lambda )^{-1} - (T_{\mathrm{MIT}} - \lambda )^{-1} \big ) \nonumber \\&= \frac{1}{(l-1)!} \frac{\text {d}^{l-1}}{\text {d} \lambda ^{l-1}} \big [ \overline{\gamma (\lambda )} \big ( \vartheta - \overline{M(\lambda )} \big )^{-1} \gamma (\overline{\lambda })^* \big ] \nonumber \\&= \sum _{p + q + r = l-1} \frac{1}{p! q! r!} \frac{\text {d}^p }{\text {d} \lambda ^p} \overline{\gamma (\lambda )} \frac{\text {d}^q}{\text {d} \lambda ^q} \big ( \vartheta - \overline{M(\lambda )} \big )^{-1} \frac{\text {d}^r}{\text {d} \lambda ^r} \gamma (\overline{\lambda })^*. \end{aligned}$$

We are going to study now all the terms on the right-hand side of (5.11) and show that they belong to certain Schatten–von Neumann ideals. For this purpose, we claim that

5.12 $$\begin{aligned} \frac{\text {d}^k}{\text {d} \lambda ^k} \left( \frac{1}{2} \beta + \mathcal {C}_\lambda \right) ^{-1} \in \mathfrak {S}_{2/k, \infty }\bigl (L^2(\partial \Omega ; \mathbb {C}^4)\bigr ) \end{aligned}$$

for $k \in \{ 1, \dots , l-1 \}$. This will be shown by induction. First, for $k = 1$ we have by (5.8)

$$\begin{aligned} \frac{\text {d}}{\text {d} \lambda } \left( \frac{1}{2} \beta + \mathcal {C}_\lambda \right) ^{-1} = -\left( \frac{1}{2} \beta + \mathcal {C}_\lambda \right) ^{-1} \frac{\text {d}}{\text {d} \lambda } \mathcal {C}_\lambda \left( \frac{1}{2} \beta + \mathcal {C}_\lambda \right) ^{-1}. \end{aligned}$$

Hence, the statement for $k=1$ holds by Lemma 3.14 and Proposition 3.12. Let us assume now that the statement holds for $k=1, \dots , q$ with $q<l-1$. With the aid of (5.7), we get

$$\begin{aligned}&\frac{\text {d}^{q+1}}{\text {d} \lambda ^{q+1}} \left( \frac{1}{2} \beta + \mathcal {C}_\lambda \right) ^{-1} = \frac{\text {d}^q}{\text {d} \lambda ^q} \left[ \frac{\text {d}}{\text {d} \lambda } \left( \frac{1}{2} \beta + \mathcal {C}_\lambda \right) ^{-1} \right] \\&\quad = -\,\frac{\text {d}^q}{\text {d} \lambda ^q} \left[ \left( \frac{1}{2} \beta + \mathcal {C}_\lambda \right) ^{-1} \frac{\text {d}}{\text {d} \lambda } \mathcal {C}_\lambda \left( \frac{1}{2} \beta + \mathcal {C}_\lambda \right) ^{-1} \right] \\&\quad = -\,\sum _{k+m+n = q} \frac{q!}{k! m! n!} \frac{\text {d}^k }{\text {d} \lambda ^k} \left( \frac{1}{2} \beta + \mathcal {C}_\lambda \right) ^{-1} \frac{\text {d}^{m+1}}{\text {d} \lambda ^{m+1}} \mathcal {C}_\lambda \frac{\text {d}^n}{\text {d} \lambda ^n} \left( \frac{1}{2} \beta + \mathcal {C}_\lambda \right) ^{-1}. \end{aligned}$$

Now Lemma 3.14, the induction hypothesis, and (5.6) show that the operator $\frac{\text {d}^{q+1}}{\text {d} \lambda ^{q+1}} \left( \frac{1}{2} \beta + \mathcal {C}_\lambda \right) ^{-1}$ belongs to $\mathfrak {S}_{2/(q+1), \infty }$, and (5.12) is proved.

Thanks to (5.10), it is now easy to see that (5.12) implies

5.13 $$\begin{aligned} \frac{\text {d}^k}{\text {d} \lambda ^k} \overline{M(\lambda )} \in \mathfrak {S}_{2/k, \infty }(\mathcal {G}_\Omega ). \end{aligned}$$

Similarly, using (5.9), (5.7), Lemma 3.14, and (5.6) we obtain

$$\begin{aligned} \frac{\text {d}^k}{\text {d} \lambda ^k} \overline{\gamma (\lambda )} = \sum _{s+t=k} \frac{k!}{s! t!} \frac{\text {d}^s}{\text {d} \lambda ^s} \Phi _{\lambda } \frac{\text {d}^t}{\text {d} \lambda ^t} \left( \frac{1}{2} \beta + \mathcal {C}_\lambda \right) ^{-1} \end{aligned}$$

and hence

5.14 $$\begin{aligned} \frac{\text {d}^k}{\text {d} \lambda ^k} \overline{\gamma (\lambda )} \in \mathfrak {S}_{4/(2 k+1), \infty }\bigl (\mathcal {G}_\Omega ,L^2(\partial \Omega ; \mathbb {C}^4)\bigr ). \end{aligned}$$

By taking adjoints, this implies that also

5.15 $$\begin{aligned} \frac{\text {d}^k}{\text {d} \lambda ^k} \gamma (\overline{\lambda })^* \in \mathfrak {S}_{4/(2 k+1), \infty }\bigl (L^2(\partial \Omega ; \mathbb {C}^4),\mathcal G_\Omega \bigr ). \end{aligned}$$

Note that (5.13) yields

5.16 $$\begin{aligned} \frac{\text {d}^k}{\text {d} \lambda ^k} \big ( \vartheta - \overline{M(\lambda )} \big )^{-1} \in \mathfrak {S}_{2/k, \infty }(\mathcal {G}_\Omega ); \end{aligned}$$

this can be shown in the same way as (5.12). Thus, using (5.11), (5.14), (5.15), (5.16), and  (5.6), we finally get that

$$\begin{aligned} (A_\vartheta - \lambda )^{-l} - (T_{\mathrm{MIT}} - \lambda )^{-l} \in \mathfrak {S}_{2/l, \infty }\bigl (L^2(\partial \Omega ; \mathbb {C}^4)\bigr ), \end{aligned}$$

which is the claimed result. $\square$

In the following corollary, we discuss the special case $l=3$ in Theorem 5.7. Then, the difference of the third powers of the resolvents of $A_\vartheta$ and $T_\text {MIT}$ belongs to the trace class ideal. By [64, Chapter 0, Theorem 8.2] or [60, Problem 25], this implies that the wave operators for the scattering pair $\{ A_\vartheta , T_{\mathrm{MIT}} \}$ exist and are complete, and hence the absolutely continuous parts of $A_\vartheta$ and $T_{\mathrm{MIT}}$ are unitarily equivalent. Moreover, we state an explicit formula for the trace of $(A_\vartheta - \lambda )^{-3} - (T_{\mathrm{MIT}} - \lambda )^{-3}$ in terms of the Weyl function *M*; this formula can be shown in exactly the same way as in [10, Theorem 4.6].

#### Corollary 5.8

Assume that $\Omega$ has a $C^3$-smooth boundary, let $a \in (\frac{1}{2}, 1 ]$, and let $\vartheta \in {\mathrm {Lip}}_a({\partial \Omega })$ be a real-valued function such that $|\vartheta (x)| \ne 1$ for all $x \in \partial \Omega$. Let $A_\vartheta$ be defined by (5.2) and let $T_{\mathrm{MIT}}$ be the MIT bag operator in (3.4). Then, the operator $(A_\vartheta - \lambda )^{-3} - (T_{\mathrm{MIT}} - \lambda )^{-3}$ belongs to the trace class ideal and

$$\begin{aligned} \text{ tr } \big [ (A_\vartheta - \lambda )^{-3} - (T_{\mathrm {MIT}} - \lambda )^{-3} \big ] = \frac{1}{2} \mathrm {tr} \left[ \frac{\text{ d }^2}{\text{ d } \lambda ^2} \left( \big ( \vartheta - \overline{M(\lambda )} \big )^{-1} \frac{\text{ d }}{\text{ d } \lambda } \overline{M(\lambda )} \right) \right] \end{aligned}$$

holds for all $\lambda \in \mathbb {C} {\setminus } \mathbb {R}$. Moreover, the wave operators for the scattering system $\{ A_\vartheta , T_{\mathrm{MIT}} \}$ exist and are complete, and the absolutely continuous parts of $A_\vartheta$ and $T_{\mathrm{MIT}}$ are unitarily equivalent.

### Self-Adjointness and Spectral Properties of $A_{[\omega ]}$

To complement the class of boundary conditions (5.1) discussed in the previous section, now boundary conditions of the form

5.17 $$\begin{aligned} P_+ f|_{\partial \Omega } = \omega P_+ \beta f|_{\partial \Omega } \end{aligned}$$

will be treated; here $\omega : \partial \Omega \rightarrow \mathbb {R}$ is Hölder continuous of order $a > \frac{1}{2}$, as before. In particular, (5.17) for $\omega \equiv 0$ leads to the MIT bag operator $T_{\mathrm{MIT}}$ introduced in (3.4). Of course, if $\omega$ is invertible, then (5.1) and (5.17) are equivalent by setting $\vartheta =\omega ^{-1}$, but if $\omega = 0$ on some parts of $\partial \Omega$, then this correspondence is only formal.

More precisely, let $\{ \mathcal {G}_\Omega , \Gamma _0, \Gamma _1 \}$ be the quasi boundary triple from Theorem 4.1 and let $\omega : \partial \Omega \rightarrow \mathbb {R}$ be Hölder continuous of order $a > \frac{1}{2}$. Then, the Dirac operator $A_{[\omega ]}$ acting in $L^2(\Omega ; \mathbb {C}^4)$ with boundary conditions (5.17) is defined by

5.18 $$\begin{aligned} A_{[\omega ]} f&:= (-i \alpha \cdot \nabla + m \beta ) f,\nonumber \\ \mathrm {dom}\,A_{[\omega ]}&:= \{ f \in H^1(\Omega ; \mathbb {C}^4): \Gamma _0 f = \omega \Gamma _1 f \}, \end{aligned}$$

i.e., (5.17) corresponds to the abstract boundary conditions $\Gamma _0 f - \omega \Gamma _1 f = 0$, see also (2.3). Employing Theorem 2.5 instead of Theorem 2.3 one can show in a similar manner as in Theorem 5.3 the following result on the self-adjointness of $A_{[\omega ]}$:

#### Theorem 5.9

Let $a \in (\frac{1}{2}, 1 ]$ and let $\omega \in {\mathrm {Lip}}_a({\partial \Omega })$ be a real-valued function such that $|\omega (x)| \ne 1$ for all $x \in \partial \Omega$. Moreover, let $\gamma$ and *M* be as in Proposition 4.2. Then, the operator $A_{[\omega ]}$ in (5.18) is self-adjoint in $L^2(\Omega ; \mathbb {C}^4)$ and the resolvent formula

$$\begin{aligned} (A_{[\omega ]}-\lambda )^{-1} = \big (T_{\mathrm{MIT}} - \lambda \big )^{-1} + \gamma (\lambda ) \big ( I_4 - \omega M(\lambda ) \big )^{-1} \omega \gamma (\overline{\lambda })^* \end{aligned}$$

holds for all $\lambda \in \rho (A_{\vartheta })\cap \rho (T_{\mathrm{MIT}})$.

Similarly to Sect. 5.1, one can prove now several results about the spectral properties of $A_{[\omega ]}$. The following assertions follow from Theorem 2.5 and the Krein-type resolvent formula from Theorem 5.9 in the same way as in Theorem 5.4.

#### Theorem 5.10

Let $\Omega$ be the complement of a bounded $C^2$-domain, let $a \in (\frac{1}{2}, 1]$, let $\omega \in {\mathrm {Lip}}_a({\partial \Omega })$ be a real-valued function such that $|\omega (x)| \ne 1$ for all $x \in \partial \Omega$, and let $A_{[\omega ]}$ be defined by (5.18). Then, the following statements hold:

(i) $$\sigma _{\mathrm{ess}}(A_{[\omega ]}) = (-\infty , - m] \cup [m, \infty )$$.
(ii) The number of discrete eigenvalues of $A_{[\omega ]}$ is finite.
(iii) $\lambda \in \sigma _{\mathrm{p}}(A_{[\omega ]}) \cap (-m, m)$ if and only if $1 \in \sigma _\mathrm{p}(\omega M(\lambda ))$.

Furthermore, like in Proposition 5.5, one can use the Weyl function *M* also to detect all eigenvalues of $A_{[\omega ]}$ in the case that $\Omega$ is a bounded domain. Here one has to use Proposition 2.6 instead of Proposition 2.4 to obtain the following result:

#### Proposition 5.11

Let $\Omega$ be a bounded $C^2$-smooth domain, let $a \in (\frac{1}{2}, 1 ]$, let $\omega \in {\mathrm {Lip}}_a({\partial \Omega })$ be real-valued such that $|\omega (x)| \ne 1$ for all $x \in \partial \Omega$, and let $A_{[\omega ]}$ be defined by (5.18). Then $\sigma (A_{[\omega ]}) = \sigma _{\mathrm{disc}}(A_{[\omega ]})$ and $\lambda$ is an eigenvalue of $A_{[\omega ]}$ if and only if there exists $\varphi \in \mathcal {G}_\Omega ^{1/2}$ such that

$$\begin{aligned} \lim _{\varepsilon \searrow 0} i \varepsilon M(\lambda + i \varepsilon ) \big ( I_4 - \omega M(\lambda + i \varepsilon ) \big )^{-1} \varphi \ne 0. \end{aligned}$$

Finally, also the proof of Theorem 5.7 can be adapted in a straightforward way to obtain a similar result for $A_{[\omega ]}$. A summary of the counterpart of Theorem 5.7 and Corollary 5.8 reads as follows:

#### Theorem 5.12

Let $\Omega$ be a $C^2$-domain with compact boundary, let $T_{\mathrm{MIT}}$ be the MIT bag operator in (3.4), let $a \in (\frac{1}{2}, 1 ]$, let $\omega \in {\mathrm {Lip}}_a({\partial \Omega })$ be a real-valued function such that $|\omega (x)| \ne 1$ for all $x \in \partial \Omega$, and let $A_{[\omega ]}$ be defined by (5.18). Moreover, let $l \in \mathbb {N}$ and, if $l>2$, assume that $\Omega$ has a $C^l$-smooth boundary. Then

5.19 $$\begin{aligned} (A_{[\omega ]} - \lambda )^{-l} - (T_{\mathrm{MIT}} - \lambda )^{-l} \in \mathfrak {S}_{2/l, \infty }\bigl (L^2(\Omega ; \mathbb {C}^4)\bigr ) \end{aligned}$$

holds for all $\lambda \in \mathbb {C} {\setminus } \mathbb {R}$. In particular, for $l=3$ the operator in (5.19) belongs to the trace class ideal and

$$\begin{aligned} {\mathrm {tr}} \big [ (A_{[\omega ]} - \lambda )^{-3} - (T_{\mathrm {MIT}} - \lambda )^{-3} \big ] = \frac{1}{2} \text{ tr } \left[ \frac{\text{ d }^2}{\text{ d } \lambda ^2} \left( \big ( I_4 - \omega M(\lambda ) \big )^{-1} \omega \frac{\text{ d }}{\text{ d } \lambda } M(\lambda ) \right) \right] \end{aligned}$$

holds for all $\lambda \in \mathbb {C} {\setminus } \mathbb {R}$. Moreover, the wave operators for the scattering system $\{ A_{[\omega ]}, T_{\mathrm{MIT}} \}$ exist and are complete, and the absolutely continuous parts of $A_{[\omega ]}$ and $T_{\mathrm{MIT}}$ are unitarily equivalent.

### On the Connection of $A_\vartheta$, $A_{[\omega ]}$, and Dirac Operators with $\delta$–Shell Interactions

In this section we assume that $\Omega _+ \subset \mathbb {R}^3$ is a bounded $C^2$-domain, we set $\Omega _- := \mathbb {R}^3 {\setminus } \overline{\Omega _+}$, and $\Sigma := \partial \Omega _\pm$. By $\nu _\pm$, we denote the unit normal vector field pointing outwards of $\Omega _\pm$ and for functions $f \in L^2(\mathbb {R}^3; \mathbb {C}^4)$ we will use the notation $f_\pm := f \upharpoonright \Omega _\pm$. Assume that $a \in (\frac{1}{2}, 1 ]$, let $\eta , \tau \in {\mathrm {Lip}}_a({\Sigma })$ be real-valued functions on $\Sigma$, and consider the formal differential expression

$$\begin{aligned} -i \alpha \cdot \nabla + m \beta + (\eta I_4 + \tau \beta ) \delta _\Sigma , \end{aligned}$$

where $\delta _\Sigma$ stands for the $\delta$-distribution supported on the interface $\Sigma$. In analogy to the case of constant interaction strengths in [12, Section 3], we introduce the associated Dirac operator in $L^2(\mathbb {R}^3; \mathbb {C}^4)$ as

5.20 $$\begin{aligned} B_{\eta , \tau } f&:= (-i \alpha \cdot \nabla + m \beta ) f_+ \oplus (-i \alpha \cdot \nabla + m \beta ) f_-, \nonumber \\ \mathrm {dom}\,B_{\eta , \tau }&:= \bigg \{ f = f_+ \oplus f_- \in H^1(\Omega _+; \mathbb {C}^4) \oplus H^1(\Omega _-; \mathbb {C}^4): \nonumber \\&\qquad i (\alpha \cdot \nu _+) (f_+|_{\Sigma } - f_-|_{\Sigma }) = -\frac{1}{2} (\eta I_4 + \tau \beta ) (f_+|_{\Sigma } + f_-|_{\Sigma }) \bigg \}. \end{aligned}$$

Note that so far singularly perturbed Dirac operators of this form with electrostatic and Lorentz scalar $\delta$–shell interactions have been studied only for constant coefficients $\eta ,\tau \in \mathbb R$, see, e.g., [4–6, 10, 12, 15, 16, 42, 49–51, 55, 56]. In particular, it is known that for constant $\eta , \tau \in \mathbb {R}$ such that $\eta ^2 - \tau ^2 = -4$ the operator $B_{\eta , \tau }$ decouples into two self-adjoint operators acting in $L^2(\Omega _\pm ; \mathbb {C}^4)$ with certain boundary conditions; cf. [12, Lemma 3.1 (ii)] and [37, Section V], [5, Section 5]. This phenomenon is referred to as *confinement*, since a particle which is located in $\Omega _\pm$ will stay in $\Omega _\pm$ for all times; in other words, the $\delta$-potential is impenetrable.

Define the projections

$$\begin{aligned} P_\pm ^{\Omega _+} := \frac{1}{2} \big ( I_4 \pm i \beta (\alpha \cdot \nu _+)\big ) \quad \text {and} \quad P_\pm ^{\Omega _-} := \frac{1}{2} \big ( I_4 \pm i \beta (\alpha \cdot \nu _-)\big ). \end{aligned}$$

Moreover, for $a \in (\frac{1}{2}, 1 ]$ and real-valued $\vartheta \in {\mathrm {Lip}}_a({\Sigma })$ denote by $A_\vartheta ^{\Omega _\pm }$ the self-adjoint operators defined in (5.2) acting in $L^2(\Omega _\pm ; \mathbb {C}^4)$. Our aim is to show that

5.21 $$\begin{aligned} B_{\eta , \tau }=A_\vartheta ^{\Omega _+} \oplus A_\vartheta ^{\Omega _-} \end{aligned}$$

for suitable $\vartheta ,\eta ,\tau \in {\mathrm {Lip}}_a({\Sigma })$. With the help of this identity, one can translate (under the appropriate assumptions on the interaction strengths $\vartheta ,\eta ,\tau$) results for Dirac operators with $\delta$-interactions to the operators $A_\vartheta ^{\Omega _\pm }$ studied in this paper, and vice versa; cf. Lemma 5.16 and Theorem 5.17 for an illustration. The following preparatory lemma will be useful.

#### Lemma 5.13

Let $\eta$ and $\tau$ be real-valued functions on $\Sigma$, assume that the identity $\eta ^2(x) - \tau ^2(x) = -4$ holds for all $x\in \Sigma$, and let $f_\pm \in H^1(\Omega _\pm ; \mathbb {C}^4)$. Then, the jump condition

5.22 $$\begin{aligned} i (\alpha \cdot \nu _+) (f_+|_\Sigma - f_-|_\Sigma ) = -\frac{1}{2} (\eta I_4 + \tau \beta ) (f_+|_\Sigma + f_-|_\Sigma ) \end{aligned}$$

is equivalent to the boundary conditions

5.23 $$\begin{aligned} \big ( (2 + \tau ) \beta - \eta I_4 \big ) P_+^{\Omega _\pm } f_\pm |_{\Sigma } = -\big ( (2 - \tau ) I_4 + \eta \beta \big ) P_+^{\Omega _\pm } \beta f_\pm |_{\Sigma }. \end{aligned}$$

#### Proof

Observe first that separating terms with $f_\pm$ in (5.22) leads to

5.24 $$\begin{aligned} \left( i (\alpha \cdot \nu _+) + \frac{1}{2} (\eta I_4 + \tau \beta ) \right) f_+|_\Sigma = \left( i (\alpha \cdot \nu _+) -\frac{1}{2} (\eta I_4 + \tau \beta ) \right) f_-|_\Sigma . \end{aligned}$$

We multiply (5.24) by $\pm i (\alpha \cdot \nu _+) + \frac{1}{2}(\eta I_4 - \tau \beta )$ and use $\beta ^2 = I_4$, $\eta ^2 - \tau ^2 = -4$, (1.8), which implies

$$\begin{aligned} i (\alpha \cdot \nu _+) (\eta I_4 + \tau \beta )= (\eta I_4 - \tau \beta )i (\alpha \cdot \nu _+), \end{aligned}$$

and $\nu _- = - \nu _+$, and arrive at the following equivalent form of (5.22):

5.25 $$\begin{aligned} \big ( 2 I_4 - (\eta I_4 - \tau \beta ) i(\alpha \cdot \nu _\pm ) \big ) f_\pm |_\Sigma = 0. \end{aligned}$$

From $\beta ^2 = I_4$ and (4.2), we see that $I_4 = P_+^{\Omega _\pm } + P_-^{\Omega _\pm } = P_+^{\Omega _\pm } + \beta P_+^{\Omega _\pm } \beta$, and thus (5.25) is equivalent to

$$\begin{aligned} \big ( 2 I_4 - (\eta I_4 - \tau \beta ) i(\alpha \cdot \nu _\pm ) \big ) P_+^{\Omega _\pm } f_\pm |_{\Sigma } = -\big ( 2 I_4 - (\eta I_4 - \tau \beta ) i(\alpha \cdot \nu _\pm ) \big ) \beta P_+^{\Omega _\pm } \beta f_\pm |_{\Sigma }. \end{aligned}$$

Multiplying both sides by $\beta$ and using $i \beta (\alpha \cdot \nu _\pm ) P_+^{\Omega _\pm } = P_+^{\Omega _\pm }$, we conclude that the jump condition (5.22) is equivalent to the boundary condition (5.23). $\square$

The next proposition provides conditions on $\vartheta ,\eta ,\tau \in {\mathrm {Lip}}_a({\Sigma })$ such that (5.21) holds.

#### Proposition 5.14

Let $a \in (\frac{1}{2}, 1]$ and let $\eta , \tau , \vartheta$ be real-valued functions on $\Sigma$. Then, the following statements hold:

(i) Assume that $\eta ,\tau \in {\mathrm {Lip}}_a(\Sigma )$, $\eta (x)^2 - \tau (x)^2 = -4$, and $\tau (x)\ne 2$ for all $x \in \Sigma$, and define
  5.26 $$\begin{aligned} \vartheta = \frac{\eta }{2 - \tau }. \end{aligned}$$
  Then, $\vartheta \in {\mathrm {Lip}}_a(\Sigma )$, $\vert \vartheta (x)\vert \ne 1$ for all $x\in \Sigma$, and (5.21) holds.
(ii) Assume that $\vartheta \in {\mathrm {Lip}}_a(\Sigma )$, $\vert \vartheta (x)\vert \ne 1$ for all $x \in \Sigma$, and define
  5.27 $$\begin{aligned} \eta = \frac{4 \vartheta }{1 - \vartheta ^2} \quad \text {and} \quad \tau = \frac{2 (1 + \vartheta ^2)}{\vartheta ^2 - 1}. \end{aligned}$$
  Then $\eta ,\tau \in {\mathrm {Lip}}_a(\Sigma )$, $\eta ^2(x) - \tau ^2(x) = -4$ for all $x\in \Sigma$, and (5.21) holds.

In particular, in both situations (i) and (ii) the operator $B_{\eta ,\tau }$ in (5.20) is self-adjoint in $L^2(\mathbb {R}^3; \mathbb {C}^4)$.

#### Proof

Assertion (5.21) in (i) and (ii) follows from Lemma 5.13; the remaining assertions on $\eta , \tau , \vartheta$ in (i) and (ii) are easy to check. In fact, to verify (5.21) in item (i) we multiply (5.23) by the matrix $( (2 - \tau )I_4 - \eta \beta )$, which is invertible by our assumptions on the functions $\eta$ and $\tau$. Using $\eta ^2 - \tau ^2 = -4$, we obtain for the left-hand side of (5.23)

$$\begin{aligned} \big ( (2 - \tau )I_4 - \eta \beta \big ) \big ( (2 + \tau ) \beta - \eta I_4 \big )P_+^{\Omega _\pm } f_\pm |_{\Sigma } = -4 \eta P_+^{\Omega _\pm } f_\pm |_{\Sigma } \end{aligned}$$

and for the right-hand side of (5.23)

$$\begin{aligned} -\big ( (2 - \tau )I_4 - \eta \beta \big ) \big ( (2 - \tau )I_4 + \eta \beta \big )P_+^{\Omega _\pm } \beta f_\pm |_{\Sigma } = -(8-4 \tau ) P_+^{\Omega _\pm } \beta f_\pm |_{\Sigma }. \end{aligned}$$

Therefore, if $\{ \mathcal {G}_{\Omega _\pm }, \Gamma _0^{\Omega _\pm }, \Gamma _1^{\Omega _\pm } \}$ denote the quasi boundary triples from Theorem 4.1, then (5.23) is equivalent to

5.28 $$\begin{aligned} \frac{\eta }{2 - \tau } \Gamma _0^{\Omega _\pm } f_\pm = \Gamma _1^{\Omega _\pm } f_\pm . \end{aligned}$$

With $\vartheta$ in (5.26), we now conclude from Lemma 5.13 that $f=f_+\oplus f_-\in \mathrm {dom}\,B_{\eta ,\tau }$ if and only if $f_\pm \in \mathrm {dom}\,A_\vartheta ^{\Omega _\pm }$, that is, the identity (5.21) is valid.

To show (5.21) in item (ii), note first that (5.27) yields $\vartheta = \frac{\eta }{2 - \tau }$. The above observation that (5.28) is equivalent to (5.23), and hence equivalent to (5.22) by Lemma 5.13, implies that $f_\pm \in \mathrm {dom}\,A_\vartheta ^{\Omega _\pm }$ if and only if $f=f_+\oplus f_-\in \mathrm {dom}\,B_{\eta ,\tau }$. Hence, (5.21) holds.

Finally, note that under the assumptions on $\eta , \tau , \vartheta$ in (i) and (ii), the operators $A_\vartheta ^{\Omega _\pm }$ are both self-adjoint in $L^2(\Omega _\pm ; \mathbb {C}^4)$ by Theorem 5.3. Hence, it follows from (5.21) that the operator $B_{\eta ,\tau }$ in (5.20) is self-adjoint in $L^2(\mathbb {R}^3; \mathbb {C}^4)$.

$$\square $$

For $a \in (\frac{1}{2}, 1 ]$ and real-valued $\omega \in {\mathrm {Lip}}_a({\Sigma })$ denote by $A_{[\omega ]}^{\Omega _\pm }$ the self-adjoint operators defined in (5.18) acting in $L^2(\Omega _\pm ; \mathbb {C}^4)$. Now we verify

5.29 $$\begin{aligned} B_{\eta , \tau }=A_{[\omega ]}^{\Omega _+} \oplus A_{[\omega ]}^{\Omega _-} \end{aligned}$$

for suitable $\omega ,\eta ,\tau \in {\mathrm {Lip}}_a({\Sigma })$, which is the counterpart of the identity (5.21). As above, one may use (5.29) to translate results for Dirac operators with $\delta$-interactions to the operators $A_{[\omega ]}^{\Omega _\pm }$, and vice versa. The next proposition provides the necessary relations between the functions $\omega ,\eta ,\tau$. The proof follows the same strategy as the proof of Proposition 5.14.

#### Proposition 5.15

Let $a \in (\frac{1}{2}, 1]$ and let $\eta , \tau , \omega$ be real-valued functions on $\Sigma$. Then, the following statements hold:

(i) Assume that $\eta ,\tau \in {\mathrm {Lip}}_a(\Sigma )$, $\eta (x)^2 - \tau (x)^2 = -4$, and $\tau (x)\ne -2$ for all $x \in \Sigma$, and define
  5.30 $$\begin{aligned} \omega = - \frac{\eta }{2 + \tau }. \end{aligned}$$
  Then, $\omega \in {\mathrm {Lip}}_a(\Sigma )$, $\vert \omega (x)\vert \ne 1$ for all $x\in \Sigma$, and (5.29) holds.
(ii) Assume that $\omega \in {\mathrm {Lip}}_a(\Sigma )$, $\vert \omega (x)\vert \ne 1$ for all $x \in \Sigma$, and define
  5.31 $$\begin{aligned} \eta = \frac{4 \omega }{\omega ^2-1} \quad \text {and} \quad \tau = \frac{2 (1 + \omega ^2)}{1-\omega ^2}. \end{aligned}$$
  Then $\eta ,\tau \in {\mathrm {Lip}}_a(\Sigma )$, $\eta ^2(x) - \tau ^2(x) = -4$ for all $x\in \Sigma$, and (5.29) holds.

In particular, in both situations (i) and (ii) the operator $B_{\eta ,\tau }$ in (5.20) is self-adjoint in $L^2(\mathbb {R}^3; \mathbb {C}^4)$.

#### Proof

Assertion (5.29) in (i) and (ii) follows from Lemma 5.13; the remaining assertions on $\eta , \tau , \omega$ in (i) and (ii) are easy to check. In fact, to verify (5.29) in item (i) we shall multiply the identity (5.23) by

$$\begin{aligned} \big ( (2 + \tau ) \beta - \eta I_4 \big )^{-1} =\frac{1}{4(2 + \tau )}\big ( (2 + \tau ) \beta + \eta I_4 \big ), \end{aligned}$$

where $\eta ^2 - \tau ^2 = -4$ was used. It follows that (5.23) is equivalent to

$$\begin{aligned} P_+^{\Omega _\pm } f_\pm |_{\Sigma }&= -\,\frac{1}{4(2 + \tau )}\big ( (2 + \tau ) \beta + \eta I_4 \big )\big ( (2 - \tau ) I_4 + \eta \beta \big ) P_+^{\Omega _\pm } \beta f_\pm |_{\Sigma }\\&= -\frac{\eta }{2 + \tau } P_+^{\Omega _\pm } \beta f_\pm |_{\Sigma }. \end{aligned}$$

Using the quasi boundary triples $\{ \mathcal {G}_{\Omega _\pm }, \Gamma _0^{\Omega _\pm }, \Gamma _1^{\Omega _\pm } \}$ from Theorem 4.1, we find that (5.23) is equivalent to

$$\begin{aligned} \Gamma _0^{\Omega _\pm } f_\pm = -\frac{\eta }{2 + \tau } \Gamma _1^{\Omega _\pm } f_\pm . \end{aligned}$$

With $\omega$ in (5.30), we now conclude from Lemma 5.13 that $f=f_+\oplus f_-\in \mathrm {dom}\,B_{\eta ,\tau }$ if and only if $f_\pm \in \mathrm {dom}\,A_{[\omega ]}^{\Omega _\pm }$, that is, the identity (5.29) is valid. To show (5.29) in (ii), one argues in the same way as in the proof of Proposition 5.14. The details are left to the reader. $\square$

A particularly interesting case in Proposition 5.15 (ii) corresponds to the choice $\omega = 0$. Since $A_{[0]}^{\Omega _\pm }=T_{\mathrm{MIT}}^{\Omega _\pm }$ by (5.18), identity (5.29) reduces to

$$\begin{aligned} B_{0,2}=T_{\mathrm{MIT}}^{\Omega _+} \oplus T_{\mathrm{MIT}}^{\Omega _-}, \end{aligned}$$

and hence identifies the orthogonal sum of the MIT bag operators with a Dirac operator with a purely Lorentz scalar $\delta$–shell potential; cf. [42, Remark 2.1].

Now we return to identities (5.21) and (5.29), and illustrate how one can translate known results for Dirac operators with $\delta$-potentials to self-adjoint Dirac operators on domains studied in this paper. Some preparation is necessary to formulate Theorem 5.17. For a $C^2$-domain $\Omega \subset \mathbb {R}^3$ with compact boundary, we introduce the orthogonal projection

$$\begin{aligned} P_\Omega : L^2(\mathbb {R}^3; \mathbb {C}^4) \rightarrow L^2(\Omega ; \mathbb {C}^4), \quad P_\Omega f = f \upharpoonright \Omega , \end{aligned}$$

and the corresponding embedding

$$\begin{aligned} \iota _\Omega : L^2(\Omega ; \mathbb {C}^4) \rightarrow L^2(\mathbb {R}^3; \mathbb {C}^4), \quad \iota _\Omega g = {\left\{ \begin{array}{ll} g &{} \text { in } \Omega , \\ 0 &{} \text { in } \Omega ^c. \end{array}\right. } \end{aligned}$$

Moreover, for $\lambda \in \mathbb {C} {\setminus } ( (-\infty , -m] \cup [m, \infty ) )$ we consider the integral operator $R_\lambda : L^2(\Omega ; \mathbb {C}^4) \rightarrow L^2(\Omega ; \mathbb {C}^4)$,

5.32 $$\begin{aligned} R_\lambda f(x) = \int _{\Omega } G_\lambda (x-y) f(y) \text {d} y, \quad x \in \Omega ,\, f \in L^2(\Omega ; \mathbb {C}^4), \end{aligned}$$

with $G_\lambda$ defined by (3.7). Note that $R_\lambda$ is the compression of the resolvent of the free Dirac operator *A* in $\mathbb {R}^3$, that is,

$$\begin{aligned} R_\lambda = P_\Omega (A - \lambda )^{-1} \iota _\Omega ; \end{aligned}$$

cf. [63, Section 1.E]. The resolvent formulas in the next preparatory lemma are now an immediate consequence of [12, Theorem 3.4], Proposition 5.14 (ii), and Proposition 5.15 (ii). We emphasize that in contrast to the previous discussion, the coefficients are first assumed to be real constants since Dirac operators with electrostatic and Lorentz scalar $\delta$–shell interactions have been studied in this case only. To avoid confusion, we use a subindex here.

#### Lemma 5.16

Let $\Omega \subset \mathbb {R}^3$ be a $C^2$-domain with compact boundary and let $R_\lambda$, $\Phi _\lambda$, and $\mathcal {C}_\lambda$, $\lambda \in \mathbb {C} {\setminus } \mathbb {R}$, be the operators in (5.32), (3.8), and (3.9), respectively. Then, the following statements hold:

(i) Assume that $\vartheta _*\in \mathbb R {\setminus }\{\pm 1\}$ and let
  $$\begin{aligned} \eta _* = \frac{4 \vartheta _*}{1 - \vartheta _*^2} \quad \text {and} \quad \tau _* = \frac{2 (1 + \vartheta _*^2)}{\vartheta _*^2 - 1}. \end{aligned}$$
  Then, the resolvent formula
  $$\begin{aligned} (A_{\vartheta _*} - \lambda )^{-1} = R_\lambda - \Phi _\lambda \big ( I_4 + (\eta _* I_4 + \tau _* \beta ) \mathcal {C}_\lambda \big )^{-1} (\eta _* I_4 + \tau _* \beta ) \Phi _{\overline{\lambda }}^* \end{aligned}$$
  holds for all $\lambda \in \mathbb {C} {\setminus } \mathbb {R}$.
(ii) Assume that $\omega _*\in \mathbb R{\setminus }\{\pm 1\}$ and let
  $$\begin{aligned} \eta _* = \frac{4 \omega _*}{\omega _*^2 - 1} \quad \text {and} \quad \tau _* = \frac{2 (1 + \omega _*^2)}{1 - \omega _*^2}. \end{aligned}$$
  Then, the resolvent formula
  $$\begin{aligned} (A_{[\omega _*]} - \lambda )^{-1} = R_\lambda - \Phi _\lambda \big ( I_4 + (\eta _* I_4 + \tau _* \beta ) \mathcal {C}_\lambda \big )^{-1} (\eta _* I_4 + \tau _* \beta ) \Phi _{\overline{\lambda }}^* \end{aligned}$$
  holds for all $\lambda \in \mathbb {C} {\setminus } \mathbb {R}$.

For the special choice $\omega _*=0$, the resolvent formula in Lemma 5.16 (ii) has the form

5.33 $$\begin{aligned} (T_{\mathrm{MIT}} - \lambda )^{-1} = R_\lambda - \Phi _\lambda \big ( I_4 + 2 \beta \mathcal {C}_\lambda \big )^{-1} 2 \beta \Phi _{\overline{\lambda }}^*,\quad \lambda \in \mathbb {C} {\setminus } \mathbb {R}. \end{aligned}$$

Using (5.33), we now obtain a more explicit description of the resolvents of the self-adjoint operators $A_\vartheta$ and $A_{[\omega ]}$ for general real-valued $\vartheta ,\omega \in {\mathrm {Lip}}_a({\partial \Omega })$ in terms of the compressed resolvent of the free Dirac operator and the operators $\Phi _\lambda$ and $\mathcal {C}_\lambda$ in (3.8) and (3.9), respectively. The next theorem is an immediate consequence of the resolvent formulas in Theorem 5.3 and Theorem 5.9 and of (5.33).

#### Theorem 5.17

Let $\Omega \subset \mathbb {R}^3$ be a $C^2$-domain with compact boundary, let $R_\lambda$, $\Phi _\lambda$, and $\mathcal {C}_\lambda$, $\lambda \in \mathbb {C} {\setminus } \mathbb {R}$, be the operators in (5.32), (3.8), and (3.9), respectively, and let $\gamma$ and *M* be as in Proposition 4.2. Then the following assertions hold:

(i) If $a \in (\frac{1}{2}, 1]$ and $\vartheta \in {\mathrm {Lip}}_a({\partial \Omega })$ is a real-valued function which satisfies $\vert \vartheta (x)\vert \not =1$ for all $x\in \partial \Omega$, then the resolvent of the self-adjoint operator $A_\vartheta$ in (5.2) admits the representation
  $$\begin{aligned} (A_\vartheta -\lambda )^{-1} = R_\lambda - \Phi _\lambda \big ( I_4 + 2 \beta \mathcal {C}_\lambda \big )^{-1} 2 \beta \Phi _{\overline{\lambda }}^* + \gamma (\lambda ) \big ( \vartheta - M(\lambda ) \big )^{-1} \gamma (\overline{\lambda })^* \end{aligned}$$
  for all $$\lambda \in \mathbb {C} {\setminus } \mathbb {R}$$.
(ii) If $a \in (\frac{1}{2}, 1]$ and $\omega \in {\mathrm {Lip}}_a({\partial \Omega })$ is a real-valued function which satisfies $\vert \omega (x)\vert \not =1$ for all $x\in \partial \Omega$, then the resolvent of the self-adjoint operator $A_{[\omega ]}$ in (5.18) admits the representation
  $$\begin{aligned} (A_{[\omega ]}-\lambda )^{-1} = R_\lambda - \Phi _\lambda \big ( I_4 + 2 \beta \mathcal {C}_\lambda \big )^{-1} 2 \beta \Phi _{\overline{\lambda }}^* + \gamma (\lambda ) \big ( I_4 - \omega M(\lambda ) \big )^{-1} \omega \gamma (\overline{\lambda })^* \end{aligned}$$
  for all $$\lambda \in \mathbb {C} {\setminus } \mathbb {R}$$.

Finally, we remark that the resolvent formulas above also hold for certain $\lambda \in \mathbb R$ which belong to the resolvent sets of the involved Dirac operators. This straightforward, but slightly more technical, generalization is not pursued further here.

## Appendix A. Mapping Properties of Integral Operators Between Sobolev Spaces

Throughout this appendix, let $\Omega$ be a $C^2$-domain in $\mathbb {R}^3$ with compact boundary $\partial \Omega$. The aim of this section is to provide results on integral operators acting between Sobolev spaces on the boundary $\partial \Omega$ which are applied in the main part of the paper to prove Proposition 3.10. Recall that the norm in $H^s(\partial \Omega ; \mathbb {C})$ for $s \in (0,1)$ is given by

$$\begin{aligned} \Vert \varphi \Vert _s^2:=\int _{{\partial \Omega }}|\varphi (x)|^2 \text {d} \sigma (x) +\int _{{\partial \Omega }}\int _{{\partial \Omega }} \frac{|\varphi (x)-\varphi (y)|^2}{|x-y|^{2+2s}} \text {d} \sigma (y) \text {d} \sigma (x) \end{aligned}$$

for $\varphi \in H^s(\partial \Omega ; \mathbb {C})$; cf. (1.9). To prove the main results of this appendix, we need some preliminary considerations. First, we recall a standard result on the growth of the integral of $|x-y|^{b-2}$ with respect to the surface measure $\sigma$. A proof can be found, e.g., in [45, Lemma 3.2 (b)].

### Lemma A.1

Given $b>0$, there exists $C>0$ such that

$$\begin{aligned} \int _{|x-y|\le \rho }|x-y|^{b-2} \hbox {d} \sigma (y)\le C \rho ^{b} \end{aligned}$$

for all $x\in {\partial \Omega }$ and $\rho > 0$. In particular, $\int _{\partial \Omega } |x-y|^{b-2} \hbox {d} \sigma (y)\le C$ uniformly in $x\in {\partial \Omega }$.

Next, we show that the multiplication operator with a Hölder continuous function $\vartheta \in \text {Lip}_a({\partial \Omega })$ is bounded in $H^s({\partial \Omega }; \mathbb {C})$ for any $0 \le s < a$.

### Lemma A.2

Let $0\le s<a\le 1$ and $\vartheta \in {\mathrm {Lip}}_a({\partial \Omega })$. Then, the operator given by the multiplication with $\vartheta$ is bounded in $H^s(\partial \Omega ; \mathbb {C})$.

### Proof

Throughout the proof, let *C* be a generic constant, which changes its value several times, and let $\varphi \in H^s({\partial \Omega }; \mathbb {C})$ be fixed. In order to get the desired result, we estimate in

A.1 $$\begin{aligned} \Vert \vartheta \varphi \Vert _s^2 = \int _{{\partial \Omega }}|(\vartheta \varphi )(x)|^2 \text {d} \sigma (x) +\int _{{\partial \Omega }}\int _{{\partial \Omega }} \frac{|(\vartheta \varphi )(x) - (\vartheta \varphi )(y)|^2}{|x-y|^{2+2s}} \text {d} \sigma (y) \text {d} \sigma (x) \end{aligned}$$

both terms on the right-hand side separately. First, since $\vartheta \in \text {Lip}_a({\partial \Omega })$ and ${\partial \Omega }$ is bounded, we have $\Vert \vartheta \Vert _{L^\infty ({\partial \Omega })} < \infty$. Therefore,

A.2 $$\begin{aligned} \int _{\partial \Omega } |(\vartheta \varphi )(x)|^2 \hbox {d} \sigma (x) \le \Vert \vartheta \Vert _{L^\infty ({\partial \Omega })}^2\int _{\partial \Omega } |\varphi (x)|^2 \hbox {d} \sigma (x) \le \Vert \vartheta \Vert _{L^\infty ({\partial \Omega })}^2 \Vert \varphi \Vert _s^2. \end{aligned}$$

To find an upper bound for the second term in (A.1), we note first that by $\vartheta \in \text {Lip}_a({\partial \Omega })$

$$\begin{aligned} |\vartheta (x)\varphi (x)-\vartheta (y)\varphi (y)|&\le |\vartheta (x)||\varphi (x)-\varphi (y)|+|\vartheta (x)-\vartheta (y)||\varphi (y)|\\&\le \Vert \vartheta \Vert _{L^\infty ({\partial \Omega })}|\varphi (x)-\varphi (y)|+C|x-y|^a|\varphi (y)|. \end{aligned}$$

This, Fubini’s theorem, and Lemma A.1 applied with $b = 2 (a - s) > 0$ imply now

A.3 $$\begin{aligned} \int _{\partial \Omega } \int _{\partial \Omega }&\frac{|\vartheta (x)\varphi (x)-\vartheta (y)\varphi (y)|^2}{|x-y|^{2+2s}} \hbox {d} \sigma (y) \hbox {d} \sigma (x)\nonumber \\&\le C\int _{\partial \Omega } \int _{\partial \Omega } \frac{|\varphi (x)-\varphi (y)|^2}{|x-y|^{2+2s}} \hbox {d} \sigma (y) \hbox {d} \sigma (x) \nonumber \\&\qquad \qquad +C\int _{\partial \Omega }|\varphi (y)|^2\int _{\partial \Omega } |x-y|^{2(a-s)-2} \hbox {d} \sigma (x) \hbox {d} \sigma (y)\nonumber \\&\le C\Vert \varphi \Vert ^2_s. \end{aligned}$$

Using (A.2) and (A.3) in (A.1), we conclude that $\Vert \vartheta \varphi \Vert _s\le C\Vert \varphi \Vert _s$ for $s<a$.

$$\square $$

Given now $0<a\le 1$ and an integral kernel $k:{\partial \Omega }\times {\partial \Omega }\rightarrow \mathbb {C}$ such that

A.4 $$\begin{aligned}&|k(x,y)|\le \frac{C}{|x-y|^{2-a}}\quad \text {for all }x\ne y,\nonumber \\&|k(x,z)-k(y,z)|\le C\frac{|x-y|^a}{|x-z|^2} \quad \text {for all }|x-y|<\frac{1}{4}\,|x-z|, \end{aligned}$$

define

A.5 $$\begin{aligned} T\varphi (x):=\int _{{\partial \Omega }}k(x,y)\varphi (y) \hbox {d} \sigma (y)\quad \text {for }x\in {\partial \Omega }. \end{aligned}$$

It is well known that if the integral kernel satisfies (A.4), then *T* is bounded in $L^2(\partial \Omega ; \mathbb {C})$ and, in particular, the integral on the right-hand side of (A.5) exists a.e. on $\partial \Omega$, see, e.g., [38, Proposition 3.10]. In the following theorem, which is the main result of this appendix, we show that *T* has even better mapping properties between Sobolev spaces on $\partial \Omega$:

### Theorem A.3

Let $0<s<a\le 1$. Then, *T* defined by (A.5) gives rise to a bounded operator

$$\begin{aligned} T:L^2({\partial \Omega }; \mathbb {C})\rightarrow H^s({\partial \Omega }; \mathbb {C}). \end{aligned}$$

### Proof

Throughout the proof, let *C* be a generic constant, which changes its value several times, and let $\varphi \in L^2({\partial \Omega }; \mathbb {C})$ be fixed. Let us estimate the two terms in

A.6 $$\begin{aligned} \Vert T \varphi \Vert _s^2 = \int _{{\partial \Omega }}|T \varphi (x)|^2 \text {d} \sigma (x) +\int _{{\partial \Omega }}\int _{{\partial \Omega }} \frac{|T \varphi (x) - T \varphi (y)|^2}{|x-y|^{2+2 s}} \text {d} \sigma (y) \text {d} \sigma (x) \end{aligned}$$

separately. To control the first one, we use (A.4), the Cauchy–Schwarz inequality, Lemma A.1, and Fubini’s theorem and get

A.7 $$\begin{aligned} \int _{\partial \Omega }|T\varphi (x)|^2 \hbox {d} \sigma (x)&\le C\int _{\partial \Omega }\bigg (\int _{\partial \Omega }\frac{|\varphi (y)|}{|x-y|^{2-a}} \hbox {d} \sigma (y)\bigg )^2 \hbox {d} \sigma (x)\nonumber \\&\le C\int _{\partial \Omega }\bigg (\int _{\partial \Omega } \frac{\hbox {d} \sigma (y)}{|x-y|^{2-a}} \bigg )\bigg (\int _{\partial \Omega }\frac{|\varphi (y)|^2\hbox {d} \sigma (y)}{|x-y|^{2-a}} \bigg ) \hbox {d} \sigma (x)\nonumber \\&\le C\int _{\partial \Omega }|\varphi (y)|^2 \hbox {d} \sigma (y). \end{aligned}$$

Let us now focus on the second term in (A.6). We set

$$\begin{aligned} A_1&:=\{(x,y,z)\in {\partial \Omega }\times {\partial \Omega }\times {\partial \Omega }:\,4|x-y|<|x-z|\},\\ A_2&:=\{(x,y,z)\in {\partial \Omega }\times {\partial \Omega }\times {\partial \Omega }:\,4|x-y|<|y-z|\},\\ A_3&:=\{(x,y,z)\in {\partial \Omega }\times {\partial \Omega }\times {\partial \Omega }:\,4|x-y|\ge \max (|x-z|,|y-z|)\}, \end{aligned}$$

and

$$\begin{aligned} K_j(x,y,z):=\frac{k(x,z)-k(y,z)}{|x-y|^{1+s}}\,\chi _{A_j}(x,y,z)\quad \text {for }j=1,2,3. \end{aligned}$$

Then,

A.8 $$\begin{aligned} \int _{\partial \Omega } \int _{\partial \Omega }&\frac{|T\varphi (x)-T\varphi (y)|^2}{|x-y|^{2+2s}} \hbox {d} \sigma (y) \hbox {d} \sigma (x) \nonumber \\&=\int _{\partial \Omega } \int _{\partial \Omega }\bigg |\int _{\partial \Omega }\frac{k(x,z)-k(y,z)}{|x-y|^{1+s}}\,\varphi (z) \hbox {d} \sigma (z)\bigg |^2 \hbox {d} \sigma (y) \hbox {d} \sigma (x)\nonumber \\&\le C\sum _{j=1}^3\int _{\partial \Omega } \int _{\partial \Omega }\bigg (\int _{\partial \Omega } |K_j(x,y,z)\varphi (z)| \hbox {d} \sigma (z)\bigg )^2 \hbox {d} \sigma (y) \hbox {d} \sigma (x). \end{aligned}$$

The three terms in the sum in the right-hand side of (A.8) are also estimated separately. In order to deal with the first one, let $\epsilon \in (0, a - s)$ be fixed. By (A.4), the Cauchy–Schwarz inequality, and Lemma A.1, we get first

A.9 $$\begin{aligned} \int _{\partial \Omega } \int _{\partial \Omega }&\bigg (\int _{\partial \Omega } |K_1(x,y,z)\varphi (z)| \hbox {d} \sigma (z)\bigg )^2 \hbox {d} \sigma (y) \hbox {d} \sigma (x)\nonumber \\&\le C\int _{\partial \Omega } \int _{\partial \Omega }\bigg (\int _{\partial \Omega } \frac{\chi _{A_1}(x,y,z)|\varphi (z)|}{|x-z|^2|x-y|^{1+s-a}} \hbox {d} \sigma (z)\bigg )^2 \hbox {d} \sigma (y) \hbox {d} \sigma (x)\nonumber \\&\le C\int _{\partial \Omega } \int _{\partial \Omega }\bigg (\int _{\partial \Omega } \frac{\hbox {d}\sigma (z)}{|x-z|^{2(1-\epsilon )}}\bigg ) \nonumber \\&\qquad \cdot \bigg (\int _{\partial \Omega } \frac{\chi _{A_1}(x,y,z)|\varphi (z)|^2}{|x-z|^{2(1+\epsilon )}|x-y|^{2-2(a-s)}} \hbox {d} \sigma (z)\bigg ) \hbox {d} \sigma (y) \hbox {d} \sigma (x)\nonumber \\&\le C\int _{\partial \Omega } \int _{\partial \Omega } \int _{\partial \Omega }\frac{\chi _{A_1}(x,y,z)|\varphi (z)|^2}{|x-z|^{2(1+\epsilon )}|x-y|^{2-2(a-s)}} \hbox {d} \sigma (z) \hbox {d} \sigma (y) \hbox {d} \sigma (x). \end{aligned}$$

Using now twice Lemma A.1, first for the integral with respect to *y* with $b = 2 (a - s) > 0$ and $\rho = \frac{1}{4} |x-z|$ and then for the integral with respect to *x* with $b = 2 (a - s - \epsilon ) > 0$, we conclude that

$$\begin{aligned}&\int _{\partial \Omega } \int _{\partial \Omega }\frac{\chi _{A_1}(x,y,z)}{|x-z|^{2(1+\epsilon )}|x-y|^{2-2(a-s)}} \hbox {d} \sigma (y) \hbox {d} \sigma (x) \\&\quad =\int _{\partial \Omega } \frac{1}{|x-z|^{2(1+\epsilon )}} \int _{|x-y| < |x-z|/4} \frac{1}{|x-y|^{2-2(a-s)}} \hbox {d} \sigma (y) \hbox {d} \sigma (x) \\&\quad \le C\int _{\partial \Omega }|x-z|^{2(a-s-\epsilon )-2} \hbox {d} \sigma (x)\le C. \end{aligned}$$

Combining this with (A.9) and Fubini’s theorem, we finally obtain

A.10 $$\begin{aligned} \int _{\partial \Omega } \int _{\partial \Omega }\bigg (\int _{\partial \Omega } |K_1(x,y,z)\varphi (z)| \hbox {d} \sigma (z)\bigg )^2 \hbox {d} \sigma (y) \hbox {d} \sigma (x) \le C \int _{\partial \Omega }|\varphi (z)|^2 \hbox {d} \sigma (z). \end{aligned}$$

Regarding the term in (A.8) containing $K_2$, we simply note the symmetry relation $K_2(x,y,z)=-K_1(y,x,z)$ and thus, Fubini’s theorem and (A.10) give

A.11 $$\begin{aligned} \int _{\partial \Omega } \int _{\partial \Omega }\bigg (\int _{\partial \Omega } |K_2(x,y,z)\varphi (z)| \hbox {d} \sigma (z)\bigg )^2 \hbox {d} \sigma (y) \hbox {d} \sigma (x) \le C \int _{\partial \Omega }|\varphi (z)|^2 \hbox {d} \sigma (z). \end{aligned}$$

Let us finally estimate the term in (A.8) containing $K_3$. For this purpose, set

$$\begin{aligned} A_0:=\{(x,y,z)\in {\partial \Omega }\times {\partial \Omega }\times {\partial \Omega }:\,4|x-y|\ge |x-z|\} \end{aligned}$$

and

$$\begin{aligned} K_0(x,y,z):=\frac{k(x,z)}{|x-y|^{1+s}}\,\chi _{A_0}(x,y,z). \end{aligned}$$

Then, because of $A_3 \subset A_0$ we easily see that

A.12 $$\begin{aligned} |K_3(x,y,z)|\le |K_0(x,y,z)|+|K_0(y,x,z)|. \end{aligned}$$

Clearly, (A.12), the triangle inequality, and Fubini’s theorem imply that

A.13 $$\begin{aligned}&\int _{\partial \Omega } \int _{\partial \Omega }\bigg (\int _{\partial \Omega } |K_3(x,y,z)\varphi (z)|\text {d} \sigma (z)\bigg )^2 \hbox {d} \sigma (y) \hbox {d} \sigma (x)\nonumber \\&\quad \le C\int _{\partial \Omega } \int _{\partial \Omega }\bigg (\int _{\partial \Omega } |K_0(x,y,z)\varphi (z)| \hbox {d} \sigma (z)\bigg )^2 \hbox {d} \sigma (y) \hbox {d} \sigma (x). \end{aligned}$$

To estimate the right-hand side of (A.13), we perform similar estimates as in (A.9). Choose $\epsilon \in (0, a - s)$. Then, it follows from (A.4) and the Cauchy–Schwarz inequality that

A.14 $$\begin{aligned}&\int _{\partial \Omega } \int _{\partial \Omega }\bigg (\int _{\partial \Omega } |K_0(x,y,z)\varphi (z)| \hbox {d} \sigma (z)\bigg )^2 \hbox {d} \sigma (y) \hbox {d} \sigma (x)\nonumber \\&\quad \le C\int _{\partial \Omega } \int _{\partial \Omega }\bigg (\int _{\partial \Omega } \frac{\chi _{A_0}(x,y,z)}{|x-z|^{2-a}} \frac{|\varphi (z)|}{|x-y|^{1+s}} \hbox {d} \sigma (z)\bigg )^2 \hbox {d} \sigma (y) \hbox {d} \sigma (x)\nonumber \\&\quad \le C\int _{\partial \Omega } \int _{\partial \Omega } \bigg (\int _{\partial \Omega } \frac{|\varphi (z)|^2}{|x-y|^{2+2s} |x-z|^{2 (1 - \epsilon )}}\text {d} \sigma (z) \bigg ) \nonumber \\&\qquad \qquad \cdot \bigg (\int _{|x-z| \le 4|x-y|} \frac{1}{|x-z|^{2(1-a + \varepsilon )}} \hbox {d} \sigma (z) \bigg ) \hbox {d} \sigma (y) \hbox {d} \sigma (x) \nonumber \\&\quad \le C\int _{\partial \Omega } \int _{\partial \Omega } \int _{\partial \Omega } \frac{|\varphi (z)|^2}{|x-y|^{2 (1 + s - a + \epsilon )} |x-z|^{2 (1 - \epsilon )}}\text {d} \sigma (z) \hbox {d} \sigma (y) \hbox {d} \sigma (x), \end{aligned}$$

where Lemma A.1 with $b = 2(a-\epsilon ) > 0$ and $\rho = 4|x-y|$ was applied in the last step. Applying now two more times Lemma A.1, first for the integral with respect to *y* with $b = 2 (a - s - \epsilon ) > 0$ and then for the integral with respect to *x* with $b = 2 \epsilon$, we find that

$$\begin{aligned}&\int _{\partial \Omega } \int _{\partial \Omega } \frac{1}{|x-y|^{2 (1 + s - a + \epsilon )} |x-z|^{2 (1 - \epsilon )}} \hbox {d} \sigma (y) \hbox {d} \sigma (x) \\&\quad \le C \int _{\partial \Omega } \frac{1}{|x-z|^{2 (1 - \epsilon )}} \hbox {d} \sigma (x) \le C \end{aligned}$$

holds independently of *z*. Using this in (A.14), we conclude

A.15 $$\begin{aligned} \int _{\partial \Omega } \int _{\partial \Omega }\bigg (\int _{\partial \Omega } |K_0(x,y,z)\varphi (z)| \hbox {d} \sigma (z)\bigg )^2 \hbox {d} \sigma (y) \hbox {d} \sigma (x) \le C \int _{\partial \Omega } |\varphi (z)|^2 \text {d} \sigma (z). \end{aligned}$$

Hence, it follows from (A.13) and (A.15) that

A.16 $$\begin{aligned} \int _{\partial \Omega } \int _{\partial \Omega }\bigg (\int _{\partial \Omega } |K_3(x,y,z)\varphi (z)| \hbox {d} \sigma (z)\bigg )^2 \hbox {d} \sigma (y) \hbox {d} \sigma (x) \le C \int _{\partial \Omega } |\varphi (z)|^2 \text {d} \sigma (z). \end{aligned}$$

Finally, a combination of (A.8), (A.10), (A.11), and (A.16) shows that

$$\begin{aligned} \int _{\partial \Omega } \int _{\partial \Omega }\frac{|T\varphi (x)-T\varphi (y)|^2}{|x-y|^{2+2 s}} \hbox {d} \sigma (y) \hbox {d} \sigma (x) \le C \int _{\partial \Omega } |\varphi (z)|^2 \text {d} \sigma (z) \end{aligned}$$

which, together with (A.7), implies $\Vert T\varphi \Vert _s\le C\Vert \varphi \Vert _\Omega$. $\square$
